# Cobalt-Catalyzed Highly Regioselective Alkoxycarbonylation of Olefins Driven by Light

**DOI:** 10.1021/jacs.5c22182

**Published:** 2026-05-07

**Authors:** Yong Peng, Xinxin Tian, Zhusong Cao, Thanh Huyen Vuong, Norbert Steinfeldt, Baoxin Zhang, Haijun Jiao, Henrik Junge, Matthias Beller

**Affiliations:** † Leibniz-Institut für Katalyse, Rostock, 18059, Germany; ‡ Institute of Atomic Manufacturing, Beihang University, Beijing 100191, China; ⊥ Institute of Molecular Science, Shanxi University, Taiyuan 030006, China

## Abstract

Alkoxycarbonylation of olefins is a pivotal transformation in the production of esters; however, attaining high regioselectivity in this transformation, specifically using industrially relevant aliphatic olefins, remains a formidable challenge. To date, this objective has been achieved exclusively through the integration of precious Pd catalysts and costly phosphine ligands in a pressurized CO environment at high temperatures. In this study, we present a novel cobalt-catalyzed light-driven protocol for achieving excellent regioselectivity under mild conditions. Specifically, we demonstrate the efficacy of converting propylene, as well as terminal and internal linear olefins, to the corresponding terminal esters with over 90% regioselectivity, accompanied by a wide range of alcohols. Control experiments and DFT computations reveal that light promotes the formation and maintains the concentration of the key species HCo­(CO)_3_.

## Graphical abstract

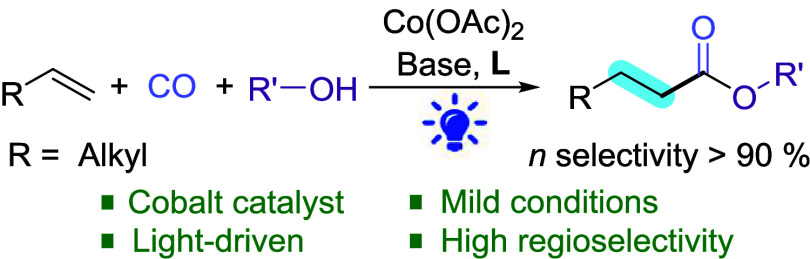

## Introduction

Carbonylation reactions enable the transformation of simple alkenes/alkynes with CO into value-added products such as esters, aldehydes, amides, and acids, which are fundamental building blocks in modern chemical industry.^1–3^ Among these transformations, alkoxycarbonylation represents a highly efficient and atom-economic route to esters and carbonylic acids, which serve as key intermediates in the production of polymers, lubricants, and fine chemicals.^4,5^ A prominent example is the palladium-catalyzed production of methyl methacrylate via the so-called Lucite Alpha process,^6^ achieving an annual output of approximately 370 000 tons in total with *Alpha 1* line in Singapore and *Alpha 2* in Saudi Arabia. Nonetheless, the selective carbonylation of higher aliphatic olefins to yield linear esters, which are generally sought after by industry, continues to be a long-standing challenge. Palladium-based catalysts, often in combination with phosphine ligands,^7^ have been the subject of extensive research. These catalysts have exhibited remarkable activity and high regioselectivity, a result of careful ligand design (Figure 1a).^8–11^ However, the utilization of costly phosphine ligands and the limited availability of precious palladium impose substantial constraints on the pursuit of sustainable processes that are in alignment with the objectives of carbon neutrality.

**Figure 1:**
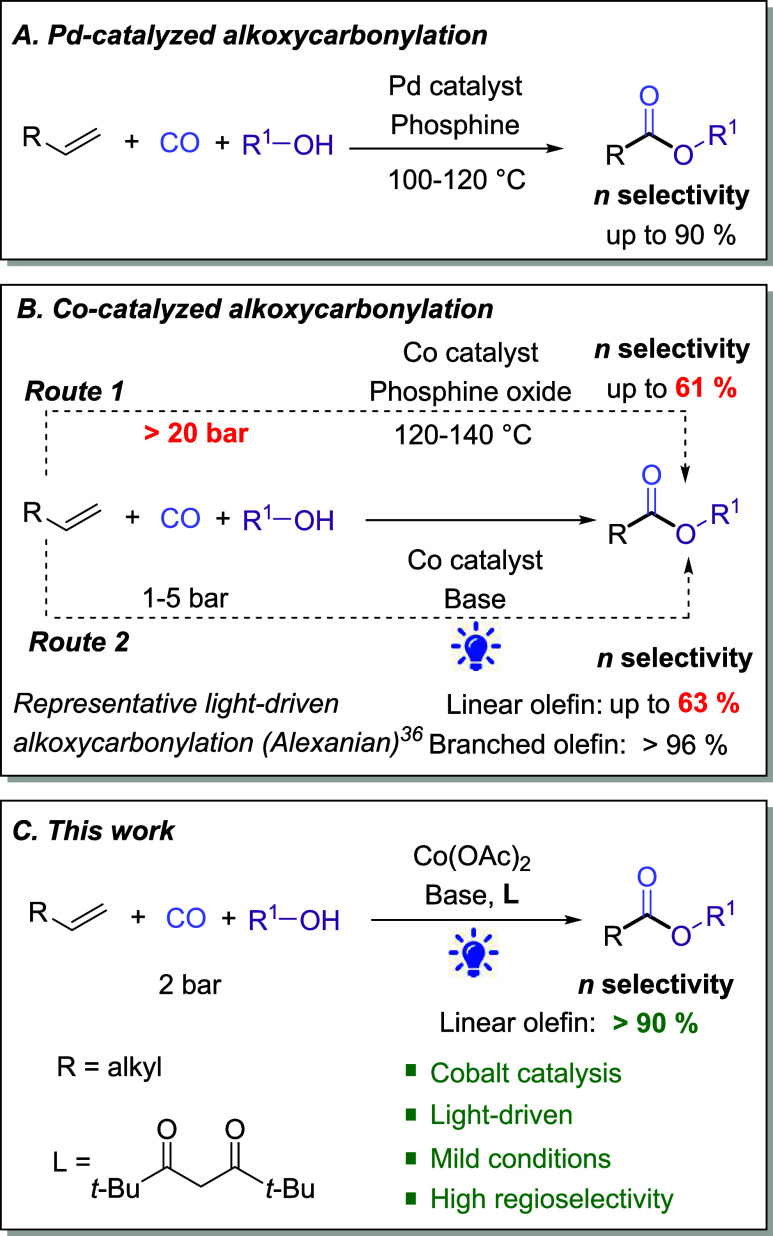
(a–c) Selected transition metal-catalyzed alkoxycarbonylation of aliphatic alkenes.

Besides palladium,^12–17^ other transition metals such as rhodium,^18^ iridium,^19^ ruthenium,^20^ platinum,^21^ nickel,^22–24^ and cobalt^25,26^ have also been explored for carbonylation reactions. Among these, Co is the most attractive candidate due to its earth abundance and notable catalytic activity. Cobalt carbonyl complexes are among the earliest known catalysts for olefin carbonylation and hydroformylation,^27^ a process historically referred to as the “Oxo synthesis,” for the industrial production of aldehydes.

However, they were gradually replaced by Rh complex catalysts over time. The latter offers the advantages of milder reaction conditions and higher selectivity. Nonetheless, in view of the mounting demand for sustainable chemical processes, there is compelling rationale for the ongoing investigation of cobalt complexes in relation to alkoxycarbonylation reactions.

Studies have demonstrated that the incorporation of phosphine oxide ligands can significantly enhance the reactivity of cobalt catalysts. However, the operation of these systems necessitates pressurized CO (>20 bar) and an elevated reaction temperature (120–140 °C) to ensure optimal performance. It is imperative to note that the regioselectivity to linear ester products remains limited (Figure 1b, Route 1), e.g., less than 61% for 1-octene.^26^

In recent years, light-driven organic synthesis has attracted considerable attention, as it enables a wide range of transformations under milder conditions with potentially reduced energy input.^28–33^ Notably, light-promoted and Co-catalyzed olefin carbonylation was reported as early as the 1990s, demonstrating high catalytic efficiency at 1 bar CO pressure, albeit using high-power mercury lamps.^34,35^ Based on their recent seminal work on light-promoted amide synthesis, a significant contribution by Alexanian et al. was published during the preparation of this manuscript. In their work, they reported the high activity and selectivity of a photoinduced and cobalt-catalyzed alkoxycarbonylation of branched/cyclic olefins (Figure 1b, Route 2).^36^ They also demonstrated a scalable protocol with low environmental impact. These results further highlighted the potential of photoassisted carbonylation strategies. Notwithstanding these advances, achieving high regioselectivity for nonactivated aliphatic olefins under mild conditions remains a significant challenge. While the pioneering work by Alexanian and co-workers established photodriven cobalt-catalyzed alkoxycarbonylation, their system was primarily optimized for branched or cyclic olefins. When applied to linear aliphatic feedstocks like propylene, the regioselectivity for the sought-after linear esters remained limited (e.g., 63%). In contrast, we report here a general and practical system using readily available, bench-stable cobalt­(II) salts and substoichiometric 1,3-dione additives. This catalytic manifold specifically addresses the challenge of “chain-walking” control, enabling unprecedented terminal selectivity (>90%) for a broad scope of both terminal and internal nonactivated alkenes under only 2 bar of CO pressure and at 60 °C, a level of performance unprecedented in cobalt catalysis under such mild conditions (Figure 1c). Such control over regioselectivity is crucial, as the formation of isomeric mixtures reduces synthetic efficiency and complicates downstream applications.

## Results

Considering the lower cost and easier handling compared to Co_2_(CO)_8_, we initiated our investigation using Co­(OAc)_2_ as cobalt precursor, also as it was used previously by Chow et al.^34^ Under 2 bar CO atmosphere, 0.2 mmol of 1-octene and 2 equiv of benzyl alcohol (0.4 mmol) were selected as model substrates in 2 mL of toluene. In the absence of a base, the substrates remained unchanged, and no ester was detected after 20 h of irradiation with 390 nm light (Table 1, entry 1). Inspired by the proposed mechanism in a recent publication on photoassisted and cobalt-catalyzed aminocarbonylation, where amines are believed to promote the formation of cobalt active species,^30^ we added one equivalent of a tertiary amine, *N*-methylpyrrolidine (NMPD), and obtained 40% yield of **3** with 84% linear selectivity (Figure S2). Further reduction of the amount of base (20 mol % NMPD) enhanced both the reactivity (47%) and linear selectivity (92%). However, reduction in the amount of base even more proved to be detrimental to the efficiency (Figure S2).

**Table 1:**
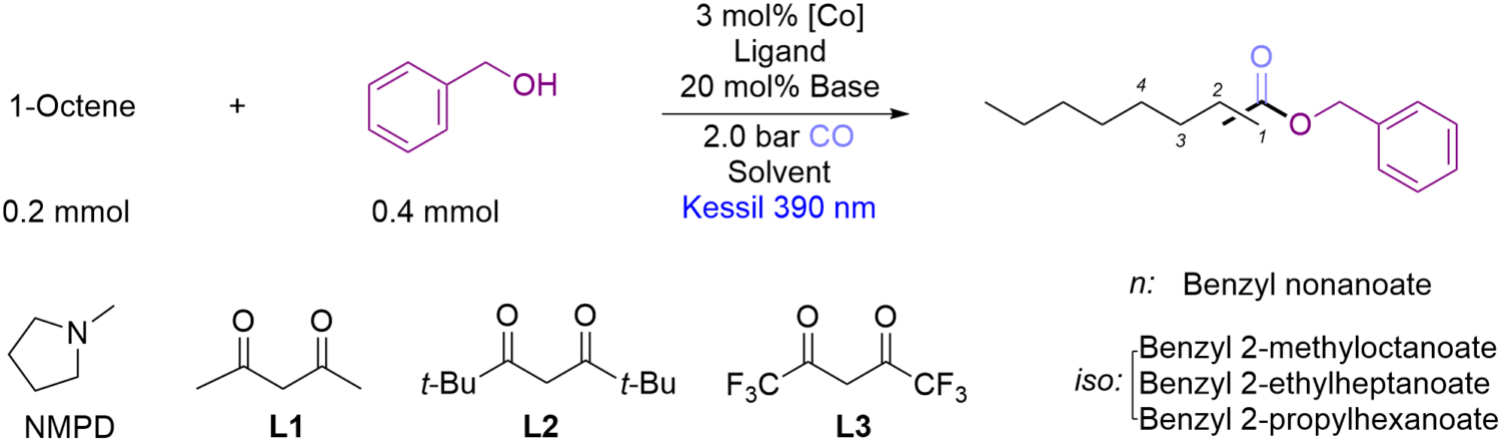
Cobalt-Catalyzed and Light-Driven Regioselective Alkoxycarbonylation of 1-Octene: Critical Reaction Parameters^a^

entry	Co catalyst (3 mol %)	base (20 mol %)	solvent	ligand (2 mol %)	yield (%)^b^	*n*/*iso* ^c^
1	Co(OAc)_2_		toluene		0	
2	Co(OAc)_2_	NMPD	toluene		62	94/6
3	Co_2_(CO)_8_	NMPD	toluene		38	95/5
4	Co(acac)_2_	NMPD	toluene		72	76/24
5	CoI_2_	NMPD	toluene		2	>99/1
6	CoCl_2_	NMPD	toluene		22	92/8
7	Co(BF_4_)_2_	NMPD	toluene		15	90/10
8	Co(OAc)_2_	TEA	toluene		46	94/6
9	Co(OAc)_2_	DMEA	toluene		56	91/9
10	Co(OAc)_2_	NaOAc	toluene		0	
11	Co(OAc)_2_	N-MePip	toluene		36	95/5
12	Co(OAc)_2_	DABCO	toluene		53	91/9
13	Co(OAc)_2_	CsOPiv	toluene		43	95/5
14	Co(OAc)_2_	DMAP	toluene		25	74/26
15	Co(OAc)_2_	Pyridine	toluene		≪ 1	>99/1
16	Co(OAc)_2_	DBU	toluene		25	72/28
17	Co(OAc)_2_	KO^t^Bu	toluene		0	
18	Co(OAc)_2_	NMPD	toluene	**L1**	79	81/19
19	Co(OAc)_2_	NMPD	toluene	**L2**	80	84/16
20	Co(OAc)_2_	NMPD	toluene	**L3**	65	92/8
21^d^	Co(OAc)_2_	NMPD	toluene	**L2**	75(72)	90/10
22^e^	Co(OAc)_2_	NMPD	toluene	**L2**	0	
23		NMPD	toluene	**L2**	0	
24^d^,^f^	Co(OAc)_2_	NMPD	toluene	**L2**	60	92/8

a Reaction conditions: 1-octene (0.2 mmol, 1 equiv), benzyl alcohol (0.4 mmol, 2 equiv), [Co] (3.0 mol %), base (20.0 mol %), solvent (2.0 mL), ligand (2.0 mol %), CO (2.0 bar), light (LED 390 nm), reaction time (20 h), reaction temperature (60 °C).

b Yields were determined by GC with hexadecane as an internal standard.

c *n*/*iso* selectivity was determined by GC.

d Using 0.5 mol % of **L2**, the isolated yield is given in parentheses.

e In the absence of light.

f 1 equiv of benzyl alcohol (0.2 mmol) was used.

Having the influence of the base concentration investigated, we next examined the effect of the cobalt precursor concentration (Figure S3) at constant base addition of 20 mol % and found high dependence of the ester formation on Co­(OAc)_2_ concentration. At a lower Co­(OAc)_2_ loading of 0.5 mol %, only 17% yield of **3** was obtained. Increasing the Co­(OAc)_2_ loading gradually improved the ester production, with a maximum yield of 62% **3** at 3 mol % of Co­(OAc)_2_ (Table 1, entry 2 and Figure S3). However, a further increase in Co concentration led to a decline in ester production. Next, various cobalt precursors were evaluated. Co_2_(CO)_8_ afforded a moderate yield of **3** (38%), albeit with excellent linear selectivity of 95% (Table 1, entry 3). In contrast, Co­(acac)_2_ led to the highest ester yield (72%), but the linear selectivity decreased to 76% (Table 1, entry 4). Other cobalt salts, including CoCl_2_, CoI_2_, and CO­(BF_4_)_2_·4H_2_O, exhibited poor activities under the applied conditions (Table 1, entries 5–7). In addition, a range of bases, including organic and inorganic ones, were tested (Table 1 entries 8–17), and NMPD was found most efficient for this transformation. Further base screening revealed that NMPD is probably not responsible for the high regioselectivity but rather increases the efficiency of the carbonylation reaction. Different solvents (*e.g*., MTBE, THF, Mesitylene, DMF, and acetonitrile) were also screened (Table S1); however, none surpassed toluene with respect to the yield of ester. Since the color of the solutions that yielded the ester product changed from violet to brown after the reaction, while the original violet color was retained until the end of the reaction in solvents in which no activity was detected, we speculated that Co^2+^ was not reduced to the active species in latter systems.

Given that Co­(acac)_2_ exhibited the highest activity and lower selectivity than Co­(OAc)_2_ (Figure S4), one could propose that combining both could achieve a high yield and high selectivity. It is found that adjusting the ratio of Co­(OAc)_2_ and Co­(acac)_2_ can increase the yield with good selectivity (Table S2). Interestingly, a comparable positive effect on the reaction outcome was obtained by the addition of a substoichiometric amount of acetylacetone (**L1**) to Co­(OAc)_2_, for example, incorporating 2 mol % **L1** resulted in 79% yield of **3** with a linear selectivity of 81% (Table 1, entry 18).

Testing other related ligands revealed that **L2** with an electron-donating group increases slightly the activity and selectivity (80% and 84%, respectively), while **L3** with electron-withdrawing groups hardly influences the activity or the selectivity (Table 1, **entries 19**–**20**). Other 1,3-dione ligands with electron-withdrawing or -donating groups and an imine-ketone ligand were tested, and none surpassed ligand **L2** in yield (). Optimizing **L2** loading ultimately afforded a 75% yield of **3** with an excellent linear selectivity of 90% (Table 1, entry 21 and Figure S5). Further control experiments showed that no reaction occurred in the absence of either light irradiation or cobalt (see Table 1, entries 22 and 23). The trial using a stoichiometric amount of benzyl alcohol was also performed, and a slightly lower yield of **3** (60%, Table 1, entry 24) was obtained. Finally, experiments at varied wavelengths revealed that **3** was not formed at 427 nm, while it was achieved in 38 and 75% yield at 370 and 390 nm with 96 and 90% linear selectivity, respectively (Table S4).

Having established the optimized conditions, we next evaluated the substrate scope of this transformation (Figure 2). In general, all tested linear α-olefins, including 1-hexane, 1-undecene, 1-tridecene and 1-tetradecene, afforded the desired products **4–8** in good yields (70–81%) with excellent linear selectivity (90–92%). In particular, propylene, a very important industrial olefin, delivered **4** in 81% yield with 91% linear selectivity, higher than the recently reported similar transformation (63%).^36^ Besides, 3,3-dimethyl-1-butene delivered product **9** in 85% yield with over 99% linear selectivity. For disubstituted terminal olefins, 2,4,4-trimethyl-1-pentene was transformed into **10** in 93% yield with up to 99% linear selectivity. In addition, (−)-β-pinene, a naturally occurring bicyclic olefin, was successfully converted to the terminal ester **11** in 45% yield with 99% linear selectivity.

**Figure 2:**
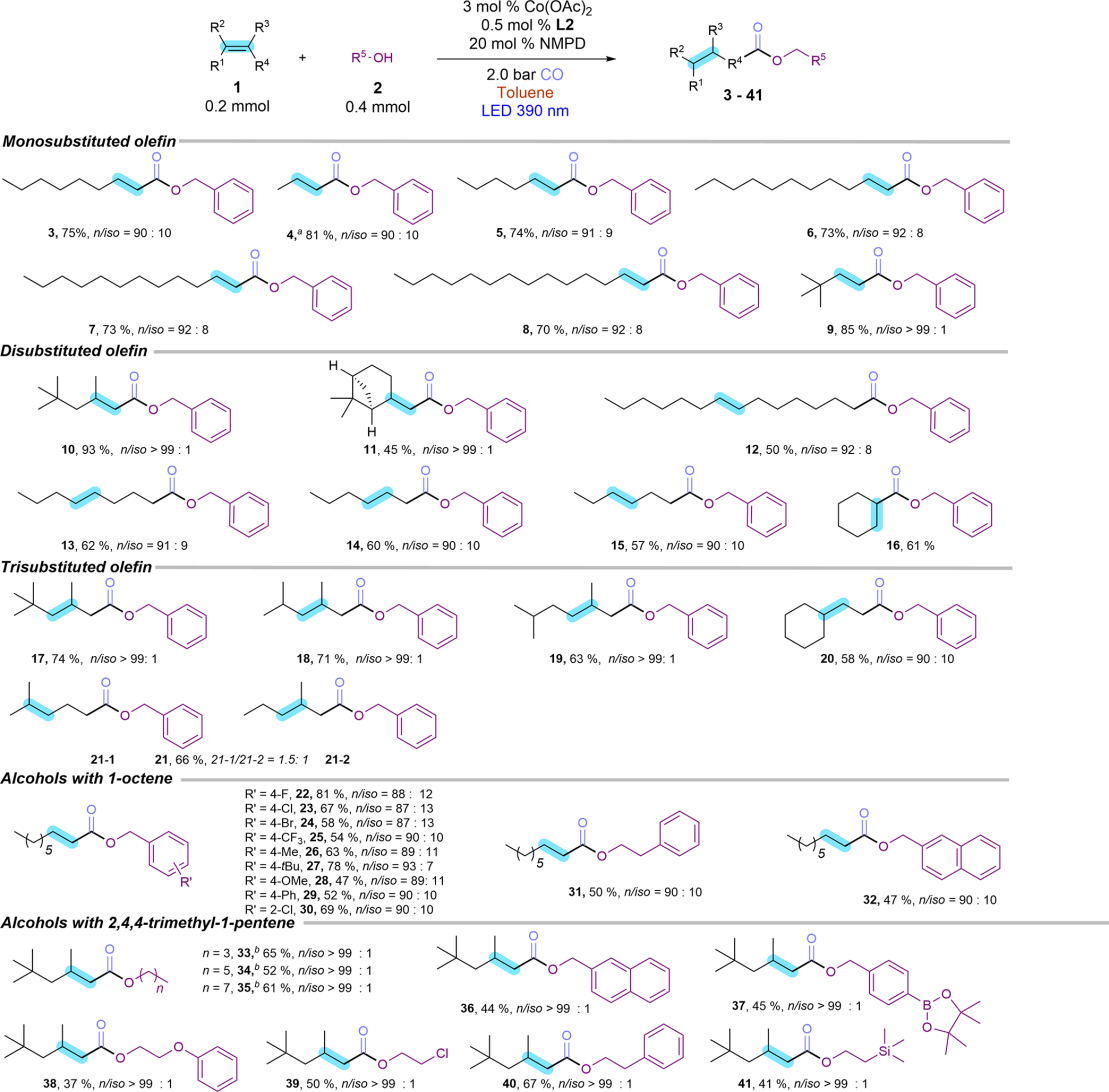
Scope of alkenes and alcohols for the regioselective alkoxycarbonylation. Reaction condition: olefins (0.2 mmol, 1 equiv), alcohols (0.4 mmol, 2 equiv), Co­(OAc)_2_ (3 mol %), NMPD (20 mol %), toluene (2 mL), **L2** (0.5 mol %), CO (2.0 bar), light (LED 390 nm), reaction time (20 h), reaction temperature (60 °C). [a] No ligand was employed, propylene (1 mmol). [b] Reaction time 40 h. Isolated yields, except **3** (GC yield, hexadecane as internal standard). [c] *n*/*iso* selectivity was determined by GC.

Furthermore, disubstituted internal olefins were smoothly converted into the corresponding terminal esters (**12–15**) in good yield (50–62%) with excellent regioselectivity. Additionally, cyclohexene afforded **16** in 61% isolated yield. Importantly, even sterically hindered trisubstituted olefins were transformed into the corresponding esters **17–21** in good yields (58–74%) with excellent regioselectivity. It should be mentioned that two terminal esters **21–1** and **21–2** (ratio of 60/40%) were obtained from 2-methyl-2-pentene, slightly favoring functionalization at the sterically less hindered terminal position.

The direct employment of internal and terminal olefin mixtures as feedstock for selective transformations is of importance for the industrial manufacture of bulk chemicals, as these olefins are inherently produced as mixtures during large-scale processes, such as steam cracking and catalytic cracking. Consequently, we tested a mixture of internal and terminal hexenes and obtained preferably the terminal ester with excellent regioselectivity (90%, Figure 3a). Notably, the diisobutene mixture results in ester product **10** in 94% yield and >99% regioselectivity (Figure 3b). Furthermore, a gram-scale experiment using 10 mmol of 1-octene afforded **3** in 67% yield (1.66 g) and 91% linear selectivity, demonstrating the practicability of this developed strategy.

**Figure 3:**
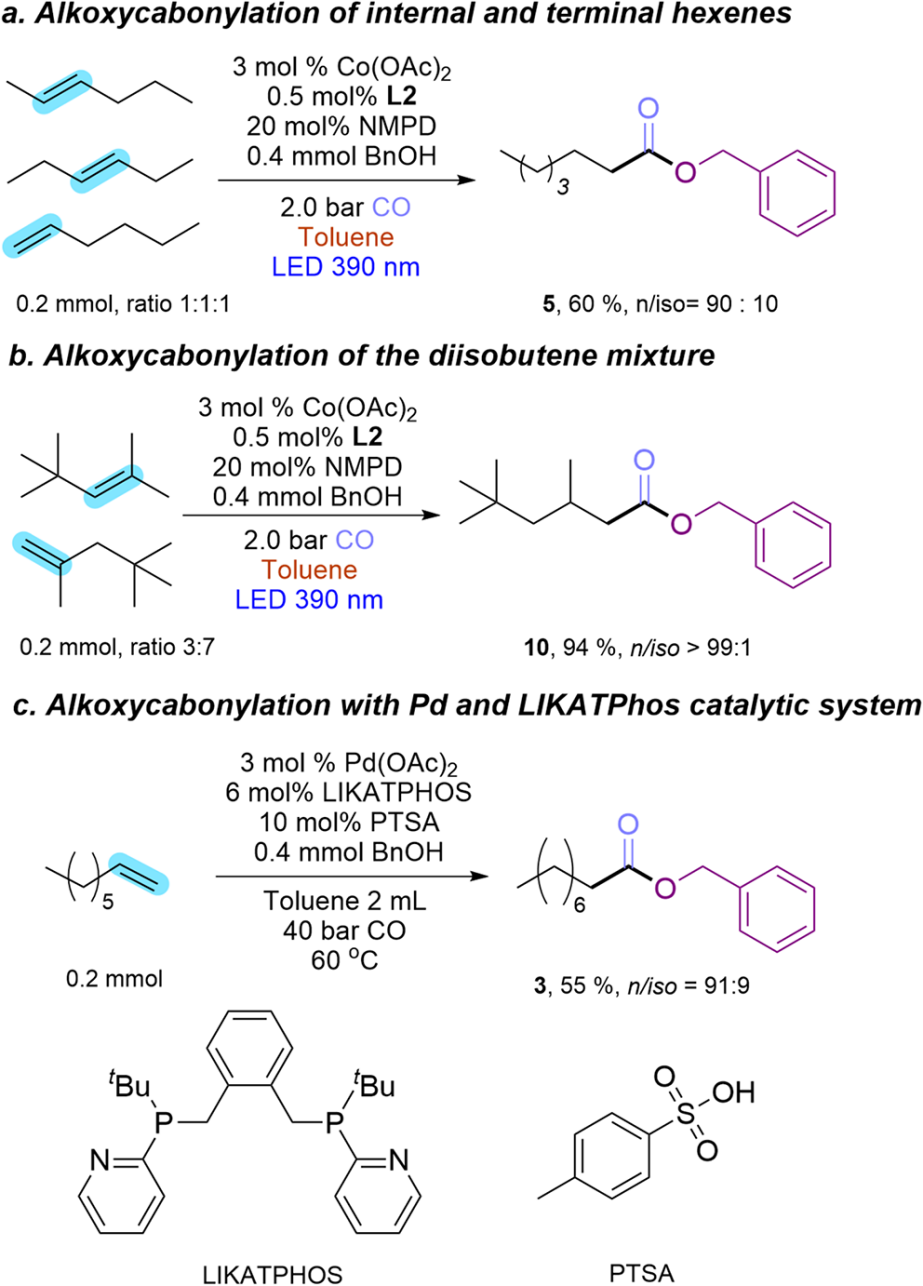
Highly selective terminal ester products from (a) terminal and internal hexene mixture, and (b) diisobutene mixture. (c) Yield and regioselectivity with Pd-Likatphos system at 60 °C and 40 bar CO pressure.

Next, we evaluated the scope of the alcohol component, employing aliphatic linear (1-octene) and branched (2,4,4-trimethyl-1-pentene) olefins as standard coupling partner. Specifically, with 1-octene as the coupling partner, several benzyl alcohol derivatives bearing either electron-withdrawing or electron-donating groups were efficiently converted into the corresponding esters **22–28** in good yields (54–85%) with very good linear selectivity (87 to 93%). Notably, 2-chlorobenzyl alcohol also furnished the corresponding ester **30** in 69% yield with a linear selectivity of 91%, despite the increased steric hindrance compared to 4-chlorobenzyl alcohol. Furthermore, an aryl substituted benzyl alcohol, biphenyl-4-methanol afforded the ester **29** in 52% yield with 90% regioselectivity. Additionally, 2-phenylethanol and 1-naphthalenemethanol were successfully converted into the corresponding esters **31** and **32** in fair yields (47–50%) with linear regioselectivity of 90%. For 2,4,4-trimethyl-1-pentene, all resulted ester products exhibited over 99% linear regioselectivity. Specifically, aliphatic alcohols furnished the ester product in good yield (**33–35**, 52–65%). Ethyl alcohols bearing phenyl, chloro, phenoxyl, and trimethylsilyl substituents afforded the corresponding esters **38–41** in 41–67% yields. Finally, 1-naphthalenemethanol and pinacol boronic ester-functionalized benzyl alcohol delivered the esters **36** and **37** in 44 and 45% yield, respectively.

In addition to alcohol, we also tested water as a nucleophile; however, no corresponding acid product was detected. Since a base is required for achieving high activity in alkoxycarbonylation, the formation of an acid might neutralize the base and consequently deactivate the system.

Noteworthy, comparing with previously reported results for similar light-activated cobalt catalysts applied in carbonylation reactions, our obtained regioselectivities for alkoxycarbonylation of 1-octene, propylene, and 3,3-dimethyl-1-butene are close to those achieved in aminocarbonylation (90/10 vs 94/6; 90/10 vs 88/12; and >99/1 vs 96/4).^30^ However, our regioselectivities are significantly higher for the alkoxy carbonylation of 4-octene (43/57) and propylene (63/37), while they are only slightly better for 3,3-dimethyl-1-butene­(96/4).^36^ The origin of the pronounced differences in product regioselectivity between these catalytic systems remains a key question. A compelling explanation, supported by recent computational studies by Bistoni et al.,^37^ is that DMAP serves as a nucleophilic catalyst in the previous system. DMAP likely intercepts the acylcobalt intermediates derived from linear olefins, thus effectively trapping them before full isomerization can take place.

We also compared our novel protocol with a state-of-the-art palladium-catalyzed alkoxycarbonylation using 1-octene as the substrate.^38^ Using the same amount of palladium loading in the presence of the Likatphos ligand (1,2-bis­((*tert*-butyl­(pyridin-2-yl)-phosphanyl)-methyl)­benzene) under 40 bar CO pressure, only 55% of **3** with 91% linear selectivity was observed (Figure 3c and the Supporting Information for the detailed reaction conditions of the Pd-based system). Conversely, our protocol operates under significantly milder conditions and avoids the use of precious metals and expensive ligands.

## Discussion

To elucidate the reaction mechanism, with particular attention to the role of light, ligands, and base, a series of control experiments were conducted, complemented by *in situ* FTIR measurements and DFT calculation. The role of light, either merely preactivating cobalt catalysts or being indispensable throughout the entire catalytic process, was first examined. For this purpose, the time course of the formation of product **3** was monitored under our standard conditions (Figure S7). An induction period of around 2 h was observed, followed by a steady ester formation, indicating the active species to be generated within the first 2 to 3 h via the reduction of Co^2+^ by benzyl alcohol. The resulting benzaldehyde, detected by GC-MS and quantified by GC, was about 8 μmol which is comparable to the amount of Co­(II) precursors (6 μmol). The underlying formal reduction reaction, 2Co­(OAc)_2_ + 8CO + 3PhCH_2_OH =2HCo­(CO)_4_ + 4HOAc + 3PhCHO, leads to the thermodynamically favored (exergonic by 24.31 kcal/mol) formation of HCo­(CO)_4_ as one of the key intermediates.

On the basis of the above-mentioned results, three control experiments were carried out (Scheme S1). First, the reaction mixture was preirradiated for 3 h, before 1-octene was added to the reaction mixture at 60 °C in the dark. No product formation was observed after 20 h. Second, the reaction mixture containing 1-octene was preirradiated for 3 h, forming **3** in only 0.8% yield, which did not further increase at 60 °C in the dark for 20 h. Third, the reaction mixture was preirradiated for 3 h. Then, 1-octene was added to the reaction mixture at 60 °C under light irradiation. Keeping light irradiation for 20 h, product **3** was formed in 56% yield with 91% linear selectivity.

In addition, a light on/off experiment was performed at constant external heating to 60 °C (Figure S8). During the first 4 h of reaction under irradiation, **3** was formed in approximately 8% yield, which increased by only 0.2% during a subsequent 2 h reaction under dark conditions. The total yield of **3** increased to 20% and 29% upon the second and third 2 h irradiation, respectively, whereas the corresponding dark intervals again resulted in only 0.2% increased yield, indicating the preactivation of precatalyst in the first run.

All of these results showed that light is indispensable for efficiently producing the ester product, while after switching the light off, the system shows only little residual activity toward product formation at constant temperature of 60 °C. This residual activity can be rationalized by the low stability of the highly active species HCo­(CO)_3_, which can turn back to HCo­(CO)_4_ under dark conditions (*vide infra*) in the absence of 1-octene or react with available 1-octene to form **3** in a rather limited yield. This shows that light irradiation is not only indispensable for producing the catalytically active species via the reduction of Co^2+^ (preactivation) but is also essential for maintaining the necessary concentration of the active species during the whole reaction cycle, as discussed in the following IR experiments.

The role of light irradiation in producing the catalytically active species via reduction of Co^2+^ was further verified by a ^1^H NMR experiment conducted in a sealed Young NMR tube (Figure S9). Under 427 nm LED light irradiation, the ^1^H NMR spectra for 1-octene and benzyl alcohol remained unchanged after 5 and 23 h, respectively, and no reaction occurred. Furthermore, the pronounced peak broadening observed in the spectra confirms the presence of paramagnetic Co^2+^ in the postreaction solution. However, when a 390 nm LED light source was applied, the steady formation of product **3** was observed after 5 as well as after 23 h. In parallel, the formation of benzaldehyde was observed, indicating the formation of the catalytically active species by Co^2+^ reduction. The same species was found before in Co^2+^ reduction tests using benzyl alcohol as a reductant (Table S1).

We were also interested in determining the proton source and the occurrence of isomerization (chain walking) that results in the terminal ester products. To this end, we employed deuterated benzyl alcohol-D_1_ (BnOD) with two olefin substrates: 1-octene, which is characterized by the mobility of its double bond, and 3,3-dimethylbutene, which possesses a nonmigratory double bond (Figure S10). The deuterium incorporation at different positions clearly demonstrates that the protons originate from alcohol and the existence of isomerization enabled by cobalt-hydride species.

Next, *in situ* FTIR spectroscopy was utilized to elucidate the process of catalyst activation. The initial investigations focused on the transformation of Co_2_(CO)_8_ as precatalyst under typical reaction conditions. As shown in Figure S11, the reaction mixture exhibited roughly the characteristic CO stretching bands (2070, 2040, and 1858 cm^–1^) of Co_2_(CO)_8_ after the sequential addition of benzyl alcohol, 1-octene, and base alongside with a reduced intensity of the bridged CO band, indicating the formation of intermediate cobalt complexes. Significant changes were observed replacing the argon by 1.3 bar of CO; three new bands in 2065, 2054, and 1898 cm^–1^ appeared immediately while the bridged CO band disappeared. This indicates the formation of monometallic complexes with reduced CO stretching frequencies.^39,40^

Under 390 nm light irradiation (Figure 4a), the vibration bands of these monometallic complexes diminished steadily with increasing reaction time up to 20 h, and simultaneously, three new vibration bands in 2020, 1934, and 1898 cm^–1^ appeared with increasing intensity. DFT calculations of IR spectra for all possible active species and catalytic intermediates were conducted and compared with experimental data (Figures S12–S17 vs Figure 4). The absorption band at 1898 cm^–1^ is primarily attributed to the [Co­(CO)_4_]^−^ anion^41,42^ (calc. 1886 cm^–1^), with a possible contribution from the first band (calc. 1881 cm^–1^) of the catalytic intermediate [Co­(CO)_2_(COC_8_H_17_)­(BnO)]^−^. The band at 1934 cm^–1^ likely results from the contributions of both [Co­(CO)_3_(COC_8_H_17_)­(BnO)]^−^ (calc. 1917 and 1960 cm^–1^) and [Co­(CO)_2_(COC_8_H_17_)­(BnO)]^−^ (the second band, calc. 1950 cm^–1^). The absorption band at 2020 cm^–1^ might come from three different potential key species, including HCo­(CO)_4_ (calc. 2036 and 2057 cm^–1^), HCo­(CO)_3_ (calc. 2031 and 2038 cm^–1^), and the catalytic intermediate [Co­(CO)_3_(COC_8_H_17_)­(BnO)]^−^ (calc. 2018 cm^–1^). Since HCo­(CO)_4_, HCo­(CO)_3_, and [Co­(CO)_3_(COC_8_H_17_)­(BnO)]^−^ are intermediates in the proposed catalytic cycle, we assume they are directly related to the catalytic activity. In addition, the ester product, with the characteristic C=O vibration band at ∼1740 cm^–1^, was observed.

**Figure 4:**
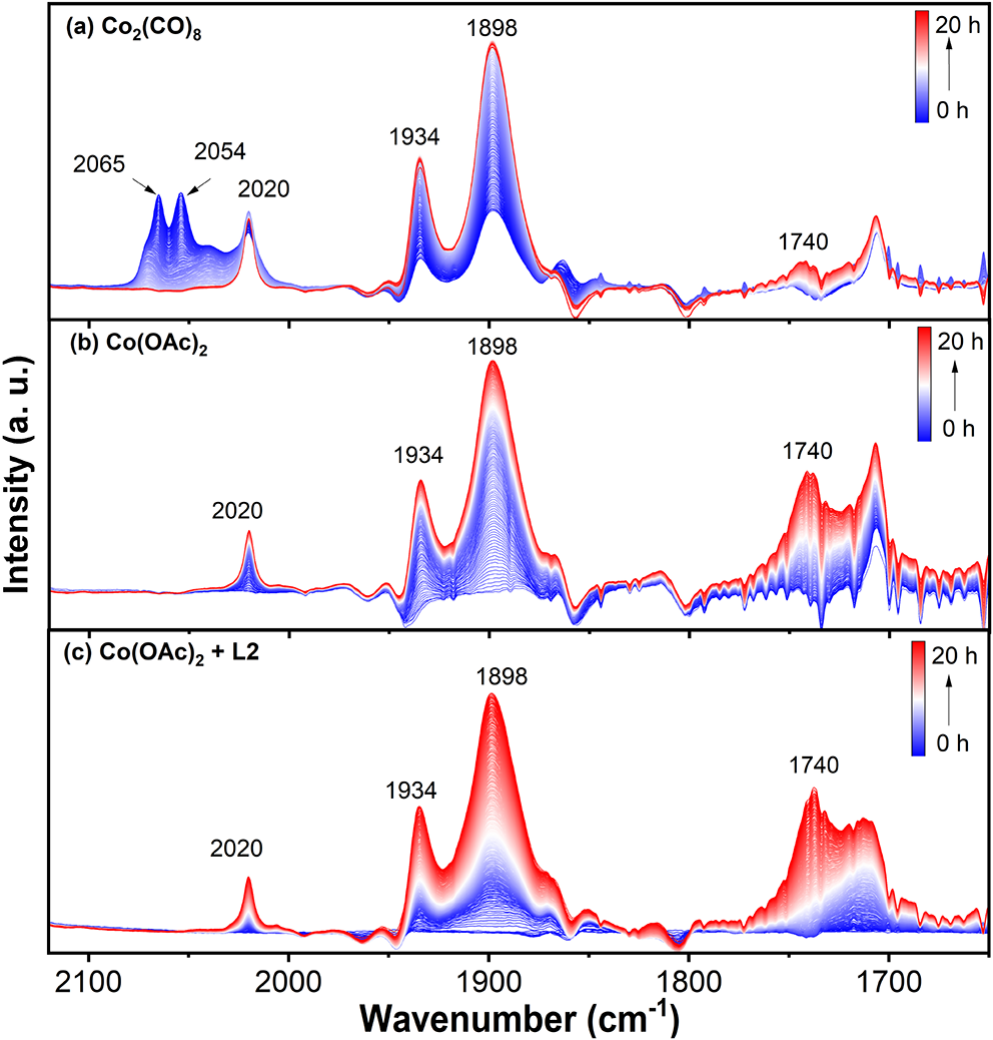
Time-resolved *in situ* FTIR spectra of catalyst activation derived from (a) Co_2_(CO)_8_, (b) Co­(OAc)_2_, and (c) Co­(OAc)_2_ with 0.5 mol % **L2**. The reaction solution was circulated between the FTIR spectrometer and a customized photoreactor via a microgear pump. Reaction conditions: toluene (10 mL), Co precursor (3 mol %), NMPD (20 mol %), **1a** (1 mmol, 1 equiv), and **1b** (2 mmol, 2 equiv) under a CO atmosphere (1.3 bar) with 390 nm LED irradiation. Spectra were collected every 5 min for 20 h.

Notably, the same reaction intermediates were observed for Co­(OAc)_2_ as precursor (Figure 4b). However, no bands corresponding to the Co(0) dimer were observed, indicating that the Co(0) dimer was not an intermediate for the active species formation. The concentration of these active species increased during the reaction (Figure 4b). In parallel, a rise in product concentration was observed. The addition of 0.5 mol % of **L2** did not result in the formation of new bands, indicating that **L2** was not involved in the active Co species (Figure 4c). The noncoordination of **L2** was confirmed by the calculated endergonic reaction Gibbs free energy [HCo­(CO)_4_ + **L2** = HCo­(CO)_2_ **L2** + 2CO, Δ*G* = 11.11 kcal/mol]. Since **L1** and **L2** can enhance the activity for ester production (Table 1, entries, 23–26) and **L3** does not, we computed the ligand exchange reaction [Co­(OAc)_2_ + 2L^–^ = CoL_2_ + 2OAc^–^], and found the exchange is exergonic for **L1** and **L2** (−31.14 and −31.95 kcal/mol, respectively) and endergonic for **L3** (24.23 kcal/mol), indicating the easy formation of complex with **L1** and **L2**. Since Co­(acac)_2_ is readily soluble in toluene whereas Co­(OAc)_2_ is not, it is reasonable to conclude that adding **L1** and **L2** allow for increased solubility of the complex in toluene, and **L3** does not. This agrees with the experimentally observed activity for Co­(OAc)_2_ using **L1**, **L2** and **L3**.

UV–vis absorption spectra of potential intermediates were recorded and complemented by TDDFT calculations. As shown in Figure S18, before irradiation, the solution exhibited absorption centered at 300 nm along with a weak absorption near 400 nm. A gradual redshift of the absorption onset was observed with increasing reaction time, indicating the formation of intermediate cobalt complexes. Further TDDFT calculation reveals that HCo­(CO)_4_ exhibits weak absorption at 389.9 nm, as reported previously,^35,43^ while [Co­(CO)_4_]^−^ has even weaker absorption at 369.5 and 378.7 nm (Figures S19 and S20). This explains the experimentally observed absorption profiles. The excitation of cobalt tetracarbonyl species by light potentially leads to the dissociation of one CO ligand, and the formation of the catalytically active species HCo­(CO)_3_ or [Co­(CO)_3_]^−^, as proposed previously.^30^ Since theoretical HCo­(CO)_4_ absorption fits better than that of [Co­(CO)_4_]^−^ to the experimental data (LED 390 nm), the former might more strongly contribute to the observed activity than the latter. This is indeed confirmed by the control experiment using NaCo­(CO)_4_ as the catalyst precursor under otherwise identical conditions. Only 16% yield of product **3** were obtained after 20 h reaction compared to 62% starting from Co­(OAc)_2_ (see the detailed reaction conditions in Section 3.3 of the Supporting Information). A simple explanation for the lower yield of **3** using NaCo­(CO)_4_ as a precatalyst is the lack of an initially generated proton source for the active species formation under these conditions. In contrast, the reduction process of Co­(OAc)_2_ (equation in Scheme 1) is computed to be exergonic by 24.31 kcal/mol and therewith thermodynamically favored. Based on the experimental and spectroscopic results, we propose that the catalytic cycle is initiated by HCo­(CO)_4_ and/or Co­(CO)_4_^–^, formed from the reduction of Co^2+^ by benzyl alcohol in the presence of light, base, and CO atmosphere.

**Scheme 1:**
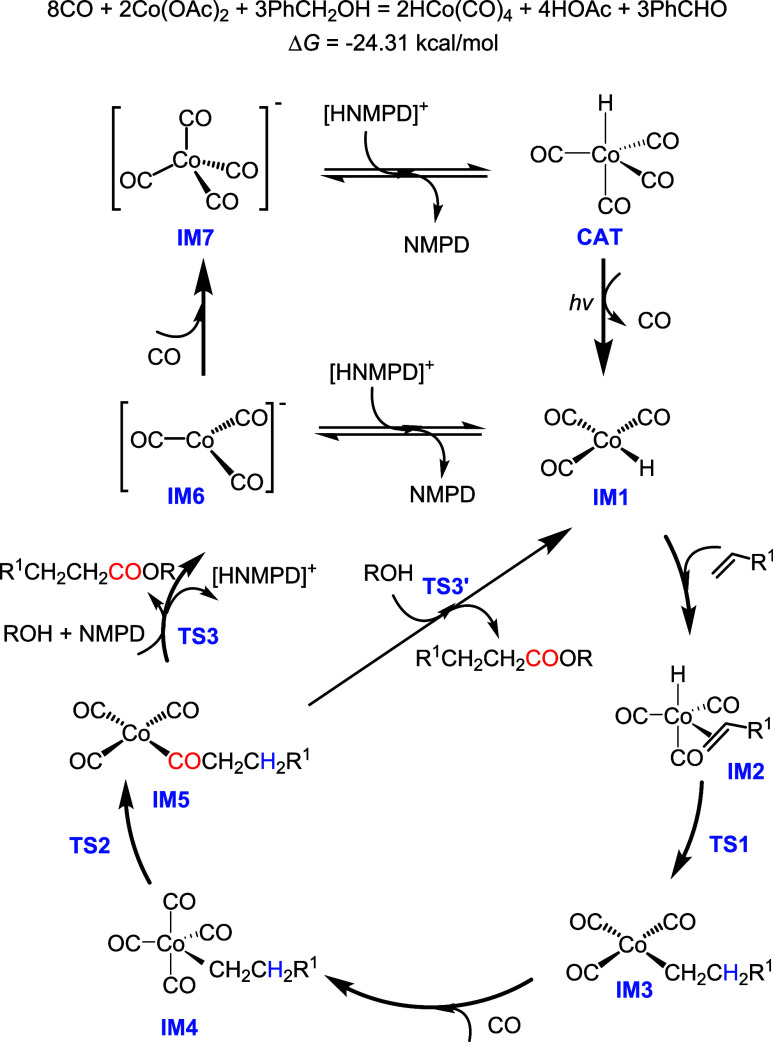
Proposed Catalytic Cycle for the Light-Driven Co-Catalyzed Alkoxycarbonylation

Having these results in hand, we propose a catalytic cycle with HCo­(CO)_3_ (**IM1**) as the active species, formed from the light-driven CO dissociation from HCo­(CO)_4_ (**CAT**) as depictured in Scheme 1, similar to the process proposed by Bistoni et al.^37^ Then, the reaction follows the well-accepted successive elementary steps, i.e., olefin coordination (**IM2**) and hydride insertion (**IM3**) resulting in two different alkyl complexes, and this is also the step for differentiating the regioselectivity, followed by further CO coordination (**IM4**) and insertion forming the corresponding acyl complexes (**IM5**). Based on **IM5**, there are two possible routes to close the cycle: one is the traditional route with oxidative addition of alcohol (**TS3′**) and subsequent reductive elimination; the other proceeds via nucleophilic substitution (**TS3**) by an alkoxide.

Based on this mechanism, we computed the catalytic cycle for 1-octene. As shown in Figure 5, the hydride insertion (**TS1**) represents the highest point on the potential energy profiles and differentiates the regioselectivity, and the computed energy difference of 1.17 kcal/mol gives a *n*/*iso* selectivity of 84/16, close to the experimentally determined 90/10 (Table 1, entry 26). For comparison (Table S5), we computed the regioselectivity of propene (**4**, ΔΔ*G* = 1.13 kcal/mol, *n*/*iso* = 83/17) and 3,3-dimethyl-1-butene (**9**, ΔΔ*G* = 2.04 kcal/mol, *n*/*iso* = 95/5) and found well agreement with the experimental values (Figure 2, 90/10 and >99/1, respectively). For the final step, the oxidative addition of benzyl alcohol (**TS3**′) needs a Gibbs free energy barrier of more than 44 kcal/mol, which is not accessible under mild reaction conditions. On the contrary, benzyloxide can bind to **IM5** and form intermediate **IM5-R1**, which can further lose one CO ligand to generate **IM**5-**R**2 (Figure 5). Based on **IM5-R1**, the reductive elimination (**TS3**) has a rather lower Gibbs free energy barrier (23.13 kcal/mol) and is exergonic by 8.13 kcal/mol, leading to the product and [Co­(CO)_3_]^−^ (**IM6**). Indeed, **TS3** can also be considered as a direct nucleophilic substitution process without coordination of benzyloxide, as suggested previously.^37,44^ Although **IM5-R2** is more stable than **IM5-R1**, the alternative step leading to the product and [Co­(CO)_2_]^−^ (**IM8**) is endergonic by 30.91 kcal/mol, and not thermodynamically competitive.

**Figure 5:**
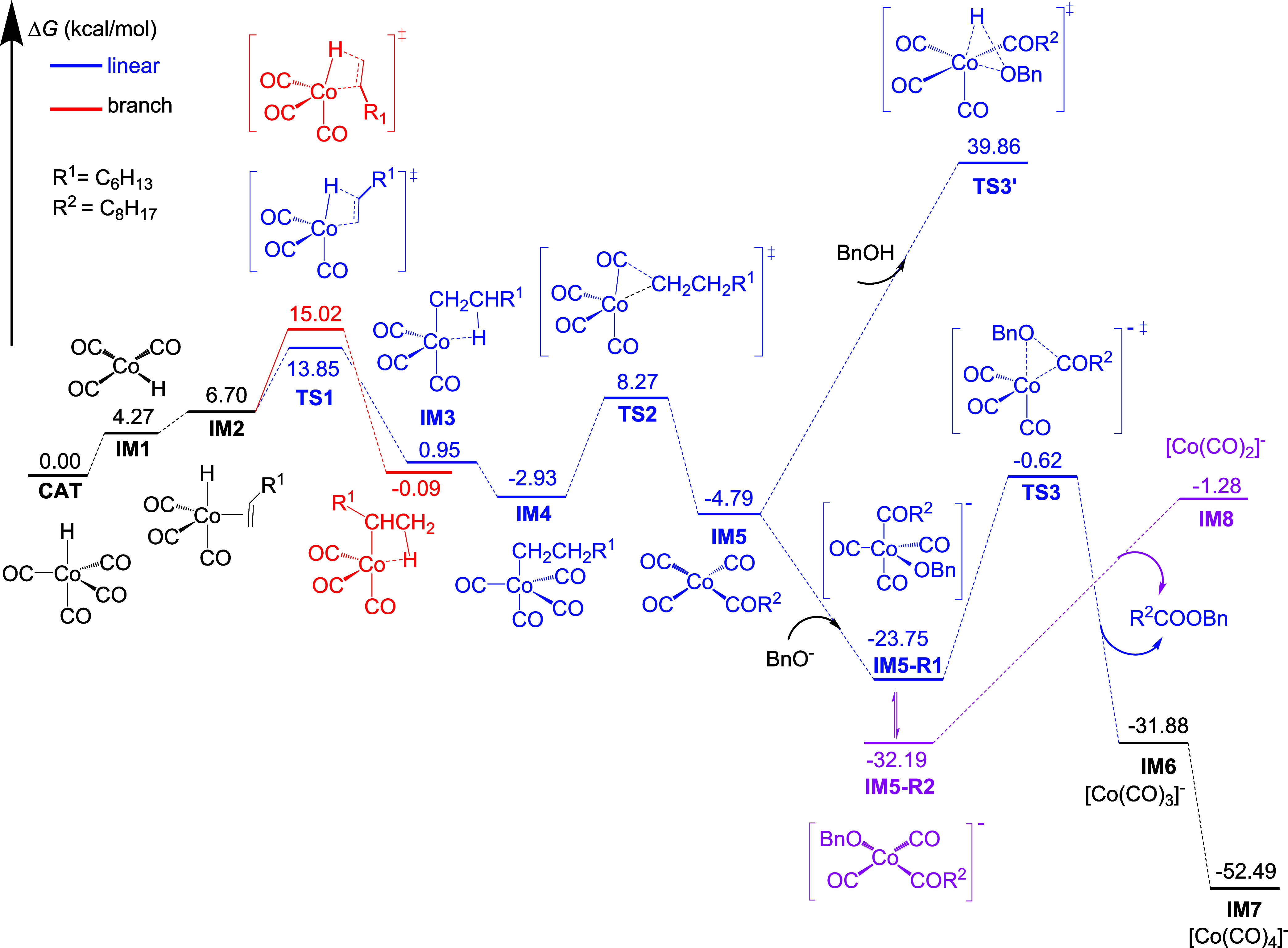
Gibbs free energy profiles of the catalytic cycle for 1-octene calculated at the M062X-SMD/6–311+G­(d) level with toluene as solvent based on the M062*X*/6–311+G­(d) optimized structures in gas phase along with thermal correction to Gibbs free energy at 358.15 K (**CAT** represents the corresponding catalyst, **IM** represents the intermediate during the reaction, and **TS** represents the transition state. The blue and red lines represent the pathways toward linear and branched esters, respectively).

It should be noted that energetic changes among the conversion and protonation equilibrium for the anionic and neutral cobalt species [Co­(CO)_4_]^−^/HCo­(CO)_4_ and [Co­(CO)_3_]^−^/HCo­(CO)_3_, proposed in Scheme 1, are not discussed, as current computational methods are not able to correctly estimate the reaction energy for systems involving separation of positive and negative charges. To demonstrate such limitation, we intentionally considered the experimentally established reaction between HCo­(CO)_4_ and pyridine (Py) in THF solution as a benchmark [HCo­(CO)_4_ + Py = [Co­(CO)_4_]^−^ + HPy^+^],^45^ where only [Co­(CO)_4_]^−^ ion was observed, revealing the deprotonation of HCo­(CO)_4_ by pyridine and therefore the thermodynamic accessibility. However, this reaction is computed to be endergonic (Table S6), on the contrary to the experimental observation of free [Co­(CO)_4_]^−^ ion in THF solution.

The Gibbs free energy surface in Figure 5 shows that the most favored reaction from HCo­(CO)_3_ (**IM1)** to [Co­(CO)_3_]^−^ (**IM6)** is exergonic by 36.15 kcal/mol. However, this energy difference does not solely reflect their interconversion but also includes the reaction energy of alkoxycarbonylation. Indeed, the conversion from **IM6** to **IM1** should undergo the proposed protonation process in Scheme 1; however, we could not discuss this energetic change due to the problem of the current computational methods as discussed above (Table S6). The even larger energy difference for the conversion from [Co­(CO)_4_]^−^ (**IM7**) to HCo­(CO)_4_ (**CAT**) or [Co­(CO)_3_]^−^ (**IM6**), and then HCo­(CO)_3_ (**IM1)** should be driven by light, as proposed in Scheme 1.

Overall, our DFT computations (Figure 5) reveal that light not only activates the catalyst but also is essential for maintaining the steady-state concentration of the coordinatively unsaturated HCo­(CO)_3_ (**IM1**) species. This species facilitates rapid “chain-walking” via successive hydride insertion/elimination steps (**TS1**). Crucially, our energy profile shows that the hydride insertion (**TS1**) represents the regioselectivity-determining step, with a calculated energy difference (1.17, 1.13, and 2.04 kcal/mol, respectively, Table S5) favoring the linear pathway for 1-octene, propene, and 3,3-dimethyl-1-butene. This energetic preference, combined with the continuous photoregeneration of **IM1**, allows for the efficient conversion of both terminal and internal olefins into a single terminal ester (>90%), a level of control over the isomerization-carbonylation sequence not observed in the DMAP-mediated manifold (Table S7, 63%), which can be rationalized by the fast interception of DMAP nucleophilic additives to acylcobalt intermediates prior to full isomerization in previous systems.

## Conclusions

In conclusion, a light-promoted cobalt-catalyzed strategy for the highly regioselective alkoxycarbonylation of olefins was developed. The synthetic protocol that has been presented herein utilizes Co­(OAc)_2_, a catalyst that is both simple and convenient to handle, operating under light irradiation and mild reaction conditions (60 °C and 2 bar CO). The substrate scope demonstrated the broad applicability of this method to both terminal and internal aliphatic olefins, affording terminal ester products with >90% regioselectivity. Furthermore, moderate to good ester yields were achieved by using a variety of alcohol nucleophiles. *In situ* FTIR analysis and DFT computations have revealed that the presence of light facilitates the reduction of Co­(OAc)_2_, resulting in the formation of HCo­(CO)_4_ and Co­(CO)_4_^–^ species. This process has also been observed to promote the subsequent formation of HCo­(CO)_3_, which has been identified as an active catalyst for the alkoxycarbonylative process. The results demonstrate that precise control over regioselectivity in cobalt-catalyzed alkoxycarbonylation of nonactivated aliphatic olefins can be achieved under mild conditions using bench-stable cobalt­(II) salts as catalyst precursor, addressing a key limitation in previously reported systems.

## Supplementary Material





## Notes

DFT-optimized coordinates (XYZ)
Experimental details, spectroscopic data, and copies of NMR spectra for all products as well as DFT computed results (PDF)

## Abbreviations

NMPD: *N*-methyl pyrrolidine
TEA: triethylamine
DMEA: dimethyl ethylamine
N-MePip: *N*-methylpiperidine
DABCO: 1,4-diazabicyclo­[2.2.2]­octane
CsOPiv: Cesium pivalate
DMAP: 4-dimethylaminopyridine
DBU: 1,8-diazabicyclo­[5.4.0]­undec-7-ene
MTBE: methyl *tert*-butyl ether
THF: tetrahydrofuran
