# Supplementary material for: Cobalt-Catalyzed Highly Regioselective Alkoxycarbonylation of Olefins Driven by Light
Source: J Am Chem Soc. 2026 May 7;148(19):19578–87. doi: 10.1021/jacs.5c22182 (PMC13195649; doi:10.1021/jacs.5c22182)
Supplement: Supplementary file 2 [file ja5c22182_si_002.pdf]

Supporting Information

Cobalt-Catalyzed Highly Regioselective Alkoxycarbonylation of Olefins Driven by Light

Yong Peng,[#*] Xinxin Tian,[#] Zhusong Cao, Thanh Huyen Vuong, Norbert Steinfeldt, Baoxin Zhang,* Haijun Jiao,* Henrik Junge,* and Matthias Beller*

*YongPeng@buaa.edu.cn, *Baoxin.Zhang@catalysis.de, *Haijun.Jiao@catalysis.de, *Henrik.Junge@catalysis.de, *Matthias.Beller@catalysis.de.

[#] These authors contributed equally to this work.

Contents

1. General information S3

2. Procedure for Photocatalytic Co-catalyzed alkoxycarbonylation S3

2.1 General procedure for alkoxycarbonylation of 1-octene with benzyl alcohol S3

2.2 Condition optimization for alkoxycarbonylation of 1-octene with benzyl alcohol ...S5

2.3 General procedure for alkoxycarbonylation of propylene with benzyl alcohol.....S12

2.4 Pd-Likatphos catalyzed alkoxycarbonylation reaction..S13

3 Mechanistic studies...S14

3.1 Synthesis of benzyl alcohol-D_1...S14

3.2 Details for DFT calculations S14

3.3 Control experiment using $NaCo(CO)_4$ as the catalyst precursor..........................S36

4. Characterizations of ester products S37

NMR spectra…………………………………………………………………….……..S40-76

Reference………………………………………………………………….……..........S77

1. General information

Chemicals were purchased from TCI, ABCR, BLD, Thermo Fisher Scientific, and Sigma Aldrich. Solvents were degassed by freeze-pump-thaw method and dried with molecular sieves or collected from SPS system. $NaCo(CO)_4$ was prepared according to the literature report.[1]

GC chromatography (Agilent technologies 6890N GC system, HP5 column/FID) was used to analyze the liquid products. Heating ramp: initial temperature 50 ^oC, heating to 260 ^oC by 8 minutes, and holding 5 minutes.

GC/MS (Agilent technologies 5973 Series insert Mass Selective Detector system, HP5 column) was used to analyze the liquid products.

NMR spectra were recorded on Bruker Avance (300 MHz/400 MHz). The ^1H NMR were calibrated by the peak of $CDCl_3$ at 7.26 ppm, DMSO-D_6 at 2.50 ppm. The ^{13}C NMR were calibrated by the peak of $CDCl_3$ at 77.16 ppm. The signals of NMR spectra are assigned as follows: s (singlet), d (doublet), t (triplet), q (quartet), and m (multiplet).

FTIR spectra were performed on a Bruker Tensor 27 FTIR spectrometer with a liquid-nitrogen-cooled MCT-A detector. A heatable transmission flow-through IR cell (Dr. Bastian Feinwerktechnik GmbH, Wuppertal, Germany) with a CaF_2 window (Korth Kristalle GmbH, Kiel, Germany) was connected to a 30 mL customized glass reactor bearing a digital pressure gauge. Reaction solution was circulated through the IR cell and back to the glass reactor by a micro gear pump (mzr-7255, HNP Mikrosysteme GmbH, Parchim, Germany). All the apparatus were connected by Swagelok quick-connects technique. OPUS v7.0 was used to control the measurement and process the acquired data. FTIR spectra ware recorded between wavenumbers of 400 and 4000 cm^{-1} with a spectral resolution of 2 cm^{-1}. Per spectrum ten scans were collected with a mirror speed set to 40 kHz

EI (electron ionization) mass spectra were recorded on an MAT 95XP spectrometer (70 eV, Thermo ELECTRON CORPORATION). ESI (electrospray ionization) high resolution mass spectra were recorded on either an Agilent Technologies 6210 TOF LC/MS or a Bruker timsTOF fleX mass spectrometer equipped with an Apollo II ESI source (Bruker Daltonics, Bremen, Germany).

UV-vis spectra were collected on a Specord 50 (Analytic Jena) UV-vis spectrometer.

Automated Combi*flash*® *Rf* Teledyne with RediSep® Rf Silver Silica Gel (12 g) disposable flash columns (40-60 μm particle size) were used for the products isolation.

2. Procedure for Photocatalytic Co-catalyzed alkoxycarbonylation

2.1 General procedure for alkoxycarbonylation of 1-octene with benzyl alcohol

A 4 mL screw-cap vial containing stirring bar was dried with heating gun under vacuum and flashed 3 times with Ar. Solvent (2 mL), 1-octene (0.2 mmol), benzyl alcohol (0.4 mmol), [Co] (3 mol %), base (20 mol %), and ligand (0.5 mol %) were added to the capped dried vial. Then the vial was fixed by a Teflon holder and transferred into autoclave type photoreactor (**Figure. S1**, note: the head and the glass reactor were purchased from *C3 PROZESS-UND ANALYSENTECHNIK*, and the bottom glass reactor holder was built by

the mechanical workshop in LIKAT). The autoclave reactor was then carefully flashed with CO for several times and then charged with 2 bars of CO. Next, the autoclave photoreactor was irradiated with Kessil® LED lamp (390 nm) for 20 h. After the reaction, the CO was carefully released to the gas line. Hexadecane was added into the after-reaction mixture, and the yield was determined by GC. Afterwards, 50 μL of DBU was added to the mixture to quench/complex the Co species and product was isolated by flash chromatograph (Column Combi*flash*®).

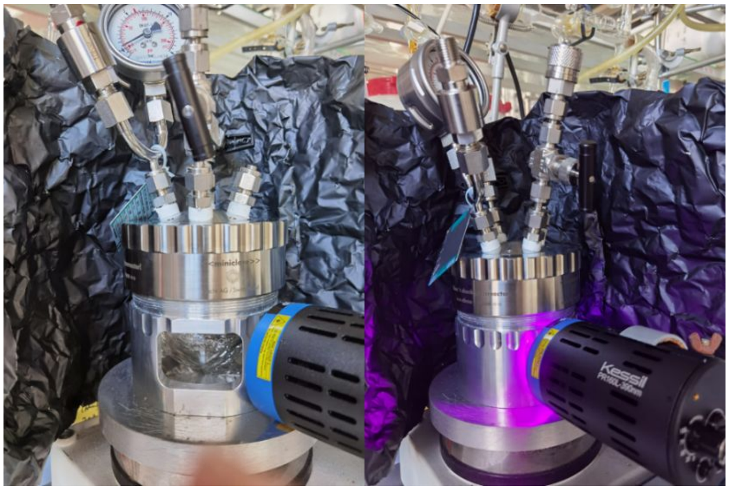

Figure S1. Photos of the autoclave type photoreactor. Up the irradiation with full power, the temperature was 60 ± 2 ^{o}C measured by IR thermometer and thermocouple. During the reaction, the reactor was covered with black foil that shows in the back of the reactor.

2.2 Condition optimization for alkoxycarbonylation of 1-octene with benzyl alcohol

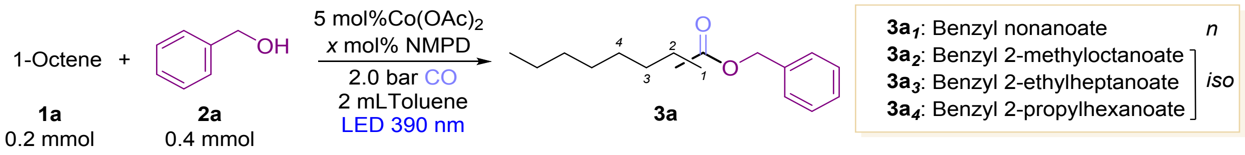

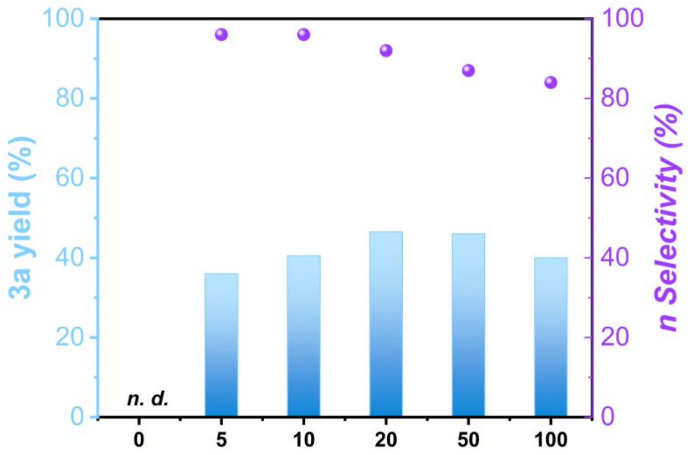

Figure S2. The influence of NMPD base concentration on the 3a yield and *n* selectivity. Reaction conditions: **1a** (0.2 mmol), **2a** (0.4 mmol), $Co(OAc)_2$ (5 mol%), NMPD (*x* mol%), toluene (2 mL), CO (2.0 bar), light (LED 390 nm), reaction time (20 h). n.d., not detected.

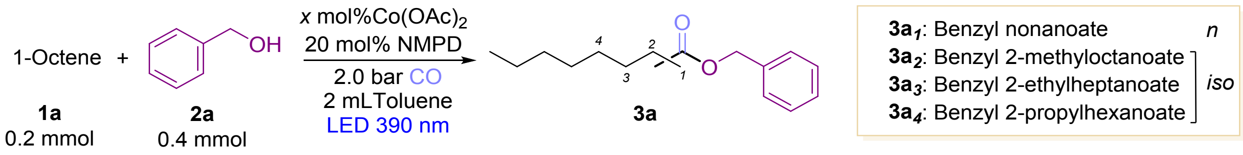

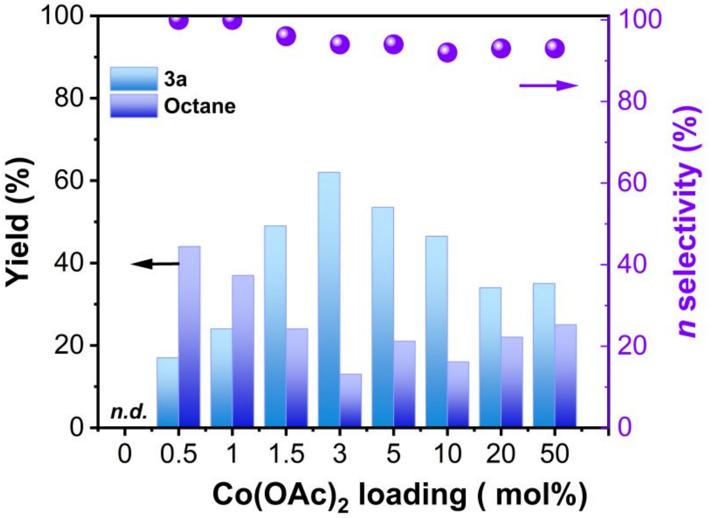

Figure S3. The influence of $Co(OAc)_2$ concentration on the 3a yield and *n* selectivity. Reaction conditions: **1a** (0.2 mmol), **2a** (0.4 mmol), $Co(OAc)_2$ (x mol%), NMPD (20 mol%), toluene (2 mL), CO (2.0 bar), light (LED 390 nm), reaction time (20 h). *n.d.,* not detected. *Yield and* n/iso selectivity determined by GC with hexadecane as the internal standard.

Table S1. Yield of **3** and *n* selectivity by photocatalytic alkoxylcarbonylation in different solvent.

Entry	Solvent	Yield (%)[b]	*n/iso*[c]
1	Toluene	62	94/6
2	MTBE	0	-
3	Acetonitrile	0	-
4	THF	0	-
5	Acetone	0	-
6	Mesitylene	34	91/9
7	1, 2-Dichloroethane	0	-
8	DMF	29	> 99/1
9	Heptane	Trace	-

[a] Reaction conditions: 1-Octene (0.2 mmol), benzyl alcohol (0.4 mmol), $Co(OAc)_2$ (3 mol%), NMPD (20 mol%), solvent (2 mL), CO (2.0 bar), light (LED 390 nm), reaction time (20 h), reaction temperature (60 ^{o}C). [b] GC yield with hexadecane as the internal standard. [c] *n/iso* determined by GC.

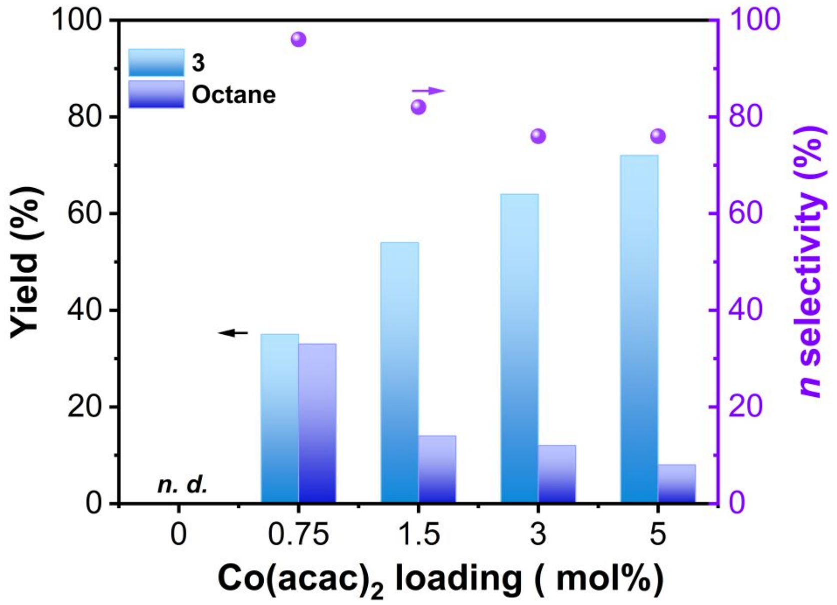

Figure S4. The influence of $Co(acac)_2$ concentration on the **3** yield and *n* selectivity. Reaction conditions: 1-Octene (0.2 mmol), benzyl alcohol (0.4 mmol), $Co(acac)_2$ (x mol%), NMPD (20 mol%), toluene (2 mL), CO (2.0 bar), light (LED 390 nm), reaction time (20 h), reaction temperature (60 ^{o}C). *n.d.,* not detected. *Yield and* n/iso selectivity determined by GC with hexadecane as the internal standard.

Table S2. Yield of **3** and *n* selectivity by photocatalytic alkoxylcarbonylation with $Co(OAc)_2$ by combination of $Co(acac)_2$ or acetylacetone.

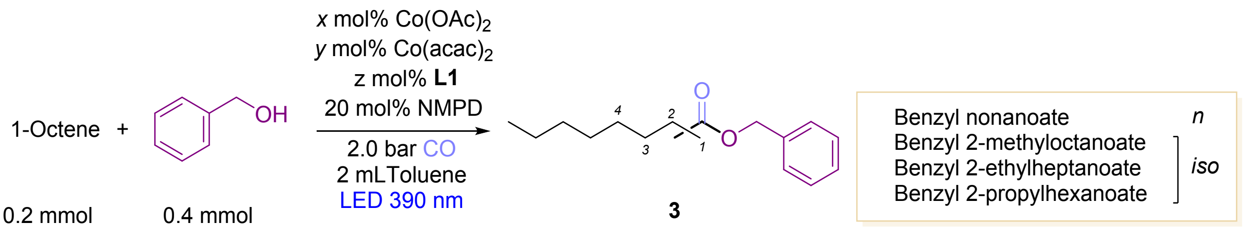

Entry	$Co(OAc)_2$ (mol%)	$Co(acac)_2$ (mol%)	Acetylacetone (mol%)	Yield (%)[b]	*n/iso*[c]
1	3	0	0	62	94/6
2	0	3	0	64	76/24
3	3	0.25	0	69	91/9
4	3	0.5	0	74	86/14
5	3	1	0	80	83/17
6	3	1.5	0	79	80/20
7	3	3	0	80	75/25
8	1.5	3	0	78	77/23
9	3	0	2	79	81/19
10	4	0	2	78	80/20
11	3	0	0.5	72	87/13

[a] Reaction conditions: 1-Octene (0.2 mmol), benzyl alcohol (0.4 mmol), $Co(OAc)_2$ (*x* mol%), $Co(acac)_2$ (*y* mol%), **L1** (*z* mol%), NMPD (20 mol%), toluene (2 mL), CO (2.0 bar), light (LED 390 nm), reaction time (20 h), reaction temperature (60 °C). [b] GC yield with hexadecane as the internal standard. [c] *n/iso* determined by GC.

Table S3. Yield of **3** and *n* selectivity in the presence of different diketone ligands.

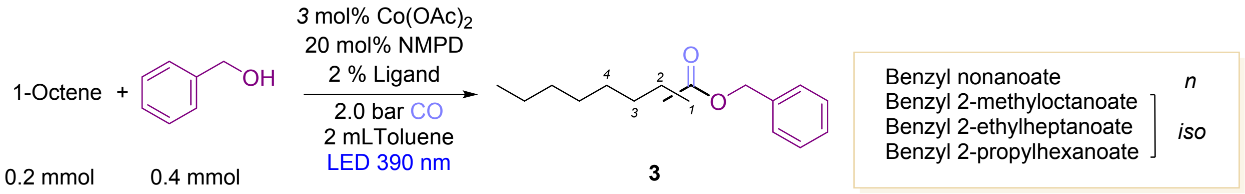

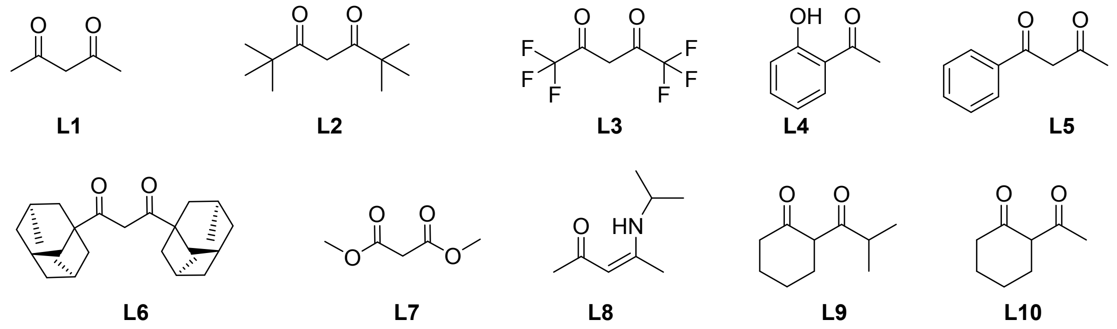

Entry	Ligand (2 mol%)	Yield (%)[b]	*n/iso*[c]
1	0	62	94/6
2	**L1**	79	81/19
3	**L2**	80	84/16
4	**L3**	65	92/8
5	**L4**	64	91/9
6	**L5**	48	77/23
7	**L6**	76	82/18
8	**L7**	67	91/9
9	**L8**	58	93/7
10	**L9**	59	93/7
11	**L10**	71	86/14

[a] Reaction conditions: 1-Octene (0.2 mmol), benzyl alcohol (0.4 mmol), $Co(OAc)_2$ (2 mol%), ligand (2 mol%), NMPD (20 mol%), toluene (2 mL), CO (2.0 bar), light (LED 390 nm), reaction temperature (60 ^{o}C), reaction time (20 h). [b] GC yield with hexadecane as the internal standard. [c] *n/iso* determined by GC.

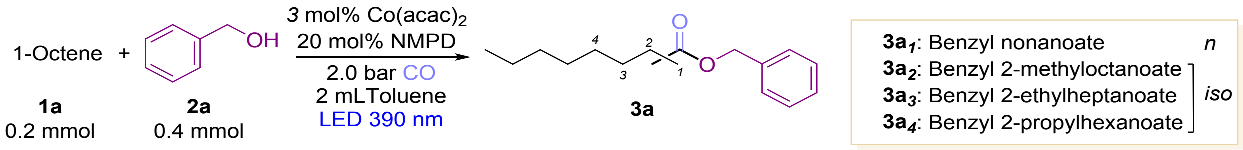

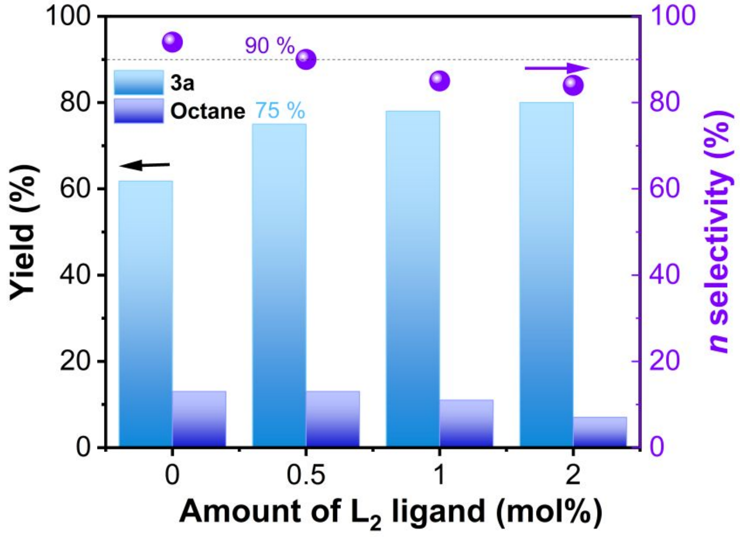

Figure S5. Influence of the **L2**concentration on the yield of **3a** and the n selectivity. Reaction conditions: **1a** (0.2 mmol), **2a** (0.4 mmol), $Co(OAc)_2$ (2 mol%), **L2** (x mol %), NMPD (20 mol%), toluene (2 mL), CO (2.0 bar), light (LED 390 nm), reaction temperature (60 ^oC), reaction time (20 h). Yield and *n/iso* selectivity determined by GC with hexadecane as the internal standard.

Table S4. The influence of light wavelength on yield of **3** and *n* selectivity with $Co(OAc)_2$ as the catalyst.

Entry	Wavelength (nm)	Yield (%)[b]	*n/iso*[c]
1	370	38	96/4
2	390	75	90/10
3	427	0	-

Reaction conditions: 1-octene (0.2 mmol), benzyl alcohol (0.4 mmol), $Co(CO)_2$ (3 mol%), NMPD (20 mol%), toluene (2 mL), CO (2.0 bar), reaction temperature (60 ^{o}C), reaction time (20 h).

2.3 General procedure for alkoxycarbonylation of propylene with benzyl alcohol

In a dried customized Schlenk tube bearing a manometer and Teflon septa (**Figure S6**), 1mmol propylene gas was charged. Followed by the injection of Toluene (10 mL), BnOH (2 mmol), $Co(OAc)_2$ (3 mol%) and base (20 mol%). Noted that for propylene substrate, ligand is not needed to add. Afterwards, 2 bar of CO was charged carefully to the reactor. Then the reaction was irradiated with Kessil lamp (390 nm) for 20 h. After the reaction, the CO was carefully released to the gas line and product **4** was obtained in 81% isolated yield by flash chromatograph (Column Combi*flash*®).

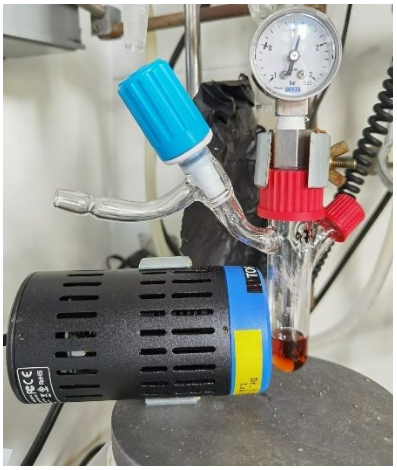

Figure S6. Photo of the Schlenk tube photoreactor setup. Up the irradiation with full power, the temperature was 60 ^{o}C measured by IR thermometer and thermocouple. During the reaction, the reactor was covered with black foil.

2.4 Pd-Likatphos catalyzed alkoxycarbonylation reaction

A screw-cap vial was charged with 3 mol% $Pd(OAc)_2$, 6 mol% Likatphos, 0.2 mmol 1-octene, 0.4 mmol benzyl alcohol, and 10 mol% *p*-toluenesulfonic acid and 2 mL toluene in a glovebox. The vial was then closed by PTFE/white rubber septum. Then the closed vial was inserted a needle from the cap and transferred into Parr 4560 series autoclave (300 mL). At room temperature, the autoclave was flushed three times with carbon monoxide and then pressurized to 40 bar with carbon monoxide. The reaction mixture was stirred at 60 °C for 20 h. After the reaction, the autoclave was cooled to room temperature, and the pressure was carefully released. The reaction mixture was analyzed by gas chromatography using hexadecane as the internal standard.

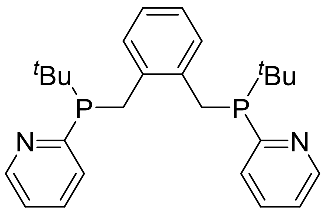

Likatphos

3 Mechanistic studies

3.1 Synthesis of benzyl alcohol-D_1

Benzyl alcohol-D_1 (BnOD) was synthesized following literature reports.[2, 3] Specifically, in a dry flask, 18 mmol of benzyl alcohol was added slowly to 36 mmol of NaH under stirring and an argon flow. After the gas evolution ceased (3 h), 2 mL of D_2O was slowly added to the flask to quench the reaction. Then BnOD was extracted of using dry diethyl ether. After drying with Na_2SO_4, the diethyl ether was removed and the crude BnOD was further deuterated by mixing with 2 mL of methanol-D_4 for 20 hours under stirring. The final BnOD was obtained by removing the methanol-D_4. The BnOD was confirmed to be 92 % pure by ^1H NMR.

3.2 Details for DFT calculations

(3.2.1) Models and methods: All calculations were performed with Gaussian 16 program.[4] Based on previous theoretical studies by Jiao et al., for $[Co(CO)_n]^{+/-}$ ionic complexes,[5] energy calculations at the B3LYP[6]/6-311+G(d) level yield results that are closely aligned with experimental CO dissociation enthalpies. Therefore, the B3LYP/6-311+G(d) method was employed for calculations.

All structures were optimized at first in gas phase and then characterized either as authentic transition states with only one imaginary frequency or energy minimums with real frequencies. Single-point energy calculations including solvation effect of toluene were then carried out by using the SMD model[7] at the same level on the gas phase optimized geometries (B3LYP-SMD/6-311+G(d)//B3LYP/6-311+G(d)).

For discussing the thermal stability, the solvated total electronic energies include the thermal correction to Gibbs free energy at 298.15 K and 1 atm were used

$HCo(CO)_4$ + **L2** = $HCo(CO)_2$**L2** + 2CO,

ΔG = 11.11 kcal/mol

$Co(OAc)_2$ +**2L**$^-$ = Co**L**$_2$ + 2OAc$^-$,

ΔG = -31.14, 31.95 and 24.23 kcal/mol for **L1**, **L2** and **L3** respectively.

$8CO + 2Co(OAc)_2 + 3PhCH_2OH = 2HCo(CO)_4 + 4HOAc + 3PhCHO$,

ΔG = -24.31 kcal/mol

(3.2.2) Calculated IR spectra:

Although B3LYP has been reported to reproduce the experimentally recorded IR of cobalt carbonyl complexes very well[5], we also tried to calculate IR using M062X. However, M062X showed larger deviation than B3LYP from the experimental results. Therefore, we used B3LYP results for discussion.

Exp.	B3LYP	M062X

$HCo(CO)_4$	2034.1,	2099.43,	2182.20,
	2058.9,	2120.73,	2215.88,
	2120.8[a]	2177.30	2261.08
$[Co(CO)_4]^-$	1890.0[b]	1944.12	1993.12

[a] Sweany, R. L. *Inorg. Chem.* **1980**, *19*, 3512
[b] Zhou, M.-F. ; Andrews, L. *J. Phys. Chem. A* **1998**, *102*, 10250

To better compare the computational results with experimental findings, we scaled the calculated IR wavenumbers by multiplying them with an empirical factor of 0.97. The corresponding obtained IR spectra are shown in **Figure S12-S17**.

The CO vibrational band of the $[Co(CO)_4]^-$ appears at 1886 cm^{-1}, which is close to the experimentally observed value of 1898 cm^{-1} in toluene solution. After desorbing one CO, the main CO vibrational peak of $[Co(CO)_3]^-$ appears at 1835 cm^{-1}.

$HCo(CO)_4$ exhibits three CO vibrational bands, located at 2036, 2057 and 2112 cm^{-1}, respectively. Among these, the peak at 2036 cm^{-1} has the highest intensity, the one at position 2057 cm^{-1} is weak, while the peak at 2112 cm^{-1} is ignorable. $HCo(CO)_3$ also shows three CO vibrational bands. The band at 2110 cm^{-1} is barely visible, while the other two are located very close to each other: 2031 and 2038 cm^{-1}.

During the catalytic cycle, the Co center in intermediate **IM5** $Co(CO)_3(COC_8H_{17})$ can coordinate with the BnO^- anion to form $[Co(CO)_3(COC_8H_{17})(BnO)]^-$ **(IM5-R1).** $[Co(CO)_3(COC_8H_{17})$ $(BnO)]^-$ has several isomeric configurations, with the most stable one featuring the acyl group occupying the axial position and the BnO^- anion located in the equatorial position. Furthermore, $[Co(CO)_3(COC_8H_{17})(BnO)]^-$ can eliminate one CO to from $[Co(CO)_2(COC_8H_{17})(BnO)]^-$ **(IM5-R2)**, the CO decoordination energy is only 0.60 kcal/mol (-0.43 kcal/mol without solvation effect). This indicates a thermodynamic equilibrium between $[Co(CO)_3(COC_8H_{17})(BnO)]^-$ and $[Co(CO)_2(COC_8H_{17})(BnO)]^-$. Three CO vibrational bands of $[Co(CO)_3(COC_8H_{17})(BnO)]^-$ locate at 1917, 1960 and 2018 cm^{-1}, respectively. And two CO vibrational bands of $[Co(CO)_3(COC_8H_{17})(BnO)]^-$ locate at 1881 and 1950 cm^{-1}, respectively.

(3.2.3) UV-vis absorption spectra:

For the calculation of the UV-Vis absorption spectra, the software ORCA[8] and B3LYP-D3(BJ)/ma-def2-QZVP theoretic level with spin-orbit coupling (SOC) and toluene solvation effect was used to directly compare with the spectra of $[Co(CO)_4]^-$ in the literature.[9] To ensure consistency with the calculation methods and structural treatments in the literature,[9] we also extended the Co-C bond length by 0.1 Å based on the optimized structure before calculating the absorption spectrum.

The obtained UV-vis absorption spectra of $HCo(CO)_4$ and $[Co(CO)_4]^-$ are shown in **Figure S19** and **Figure S20**, respectively.

TD-DFT results revealed that $HCo(CO)_4$ exhibits weak absorption at 360.9, 361.0, 363.4 and 389.9 nm, within the 360-400 nm wavelength range. The absorption at 389.9 nm is

mainly contributed by the excitation from degenerate HUMO-1 orbitals to the LUMO orbital. These excitations could potentially lead to the dissociation of a CO.

For $[Co(CO)_4]^-$ anion, it has weak absorption at 369.5, 373.1 and 378.7 nm, within the 360-400 nm wavelength range. The absorptions at 369.5 and 378.7 nm are mainly contributed by the excitation from degenerate HUMO orbitals to the LUMO orbitals, which also facilitate CO dissociation.

(3.2.4) Activity and selectivity:

For discussing the reactivity and selectivity, the thermal correction to Gibbs free energy at 358 K (60 °C) and 1 atm was included to fit the experimental temperature (B3LYP-SMD-358 K).

For the CO dissociation enthalpy of $HCo(CO)_4$, the experimental value is reported as 13.00 kcal/mol.[10] Our method testing calculations revealed that the CO dissociation enthalpy computed using M062X is 14.94 kcal/mol, which is more closer to the experimental value than the results of B3LYP method. Therefore, we also employed the M062X[11] method for calculations in the catalytic cycle section (M062X-SMD-358 K) which assumed $HCo(CO)_4$ as catalyst.

To understand the whole reaction mechanism, we systematically calculated the reaction pathways for the formation of linear and branched ester with 1-octane as substrate. As shown in **Scheme 1** in the manuscript, we assume that the reaction initiates from either $HCo(CO)_4$ or $[Co(CO)_4]^-$ anion. Under the combined action of the base NMPD and alcohol BnOH, an equilibrium exists between $HCo(CO)_4$ and $[Co(CO)_4]^-$. Similar to the situation supposed by Horvath *et al.* in their mechanism study by using IR.[12] Under light irradiation, the orbitals of $HCo(CO)_4$ or $[Co(CO)_4]^-$ are excited, promoting the dissociation of CO to form intermediates **IM1**($HCo(CO)_3$) or **IM6** ($[Co(CO)_3]^-$). With base and alcohol co-exist, **IM6** can also get a hydrogen form BnOH, realize the H transfer into **IM1**. **IM1** serves as the true active catalyst, enabling the catalytic cycle of the reaction through multiple steps, including olefine coordination, H-addition, CO insertion, and reductive elimination of the ester. The full potential energy surfaces obtained by the B3LYP and M062X methods are shown in **Figure S21** and **Figure S22**, respectively. The qualitative conclusions from both methods are consistent, but there are quantitative differences in the n/iso selectivity for different substrates. The relevant data are listed in **Table S5**.

Based on the results under M062X level, the apparent barrier of linear and branched ester formation is 13.85, 15.02 kcal/mol, respectively. The corresponding transition state is **TS1**, *i.e.* the H-addition of olefin. The barrier of CO insertion step (**TS2**) is 8.27. 8.14 kcal/mol, respectively, lower than **TS1**. In the reductive elimination step, if an BnO^- anion is used as the nucleophile, the reaction proceeds much more readily, with the free energy of **TS3** (from **IM5-R1**) being only -0.62/0.21 kcal/mol. From **IM5-R2**, the free energy of **TS3''** is slightly higher than that of **TS3** at B3LYP level, while they were not located at M062X level.

In contrast, when a neutral BnOH attacks the acyl group, the transition state **TS3'** for oxidative addition has a very high energy barrier of 46.32/46.13 kcal/mol. Such a high

barrier makes the reaction unfeasible under the current reaction temperature (60 °C). According to literature report[12], the presence of a base in the system ensures a certain concentration of BnO^- anion. Therefore, we propose that the reaction proceeds via the anionic pathway.

Based on the discussion above, we can deduce that the rate-determining step is the H-addition step, which mainly contributes to the selectivity of ester. For 1-octene substrate (Table S5), this barrier (**TS1**) difference leads to an n/iso selectivity of 84:16, very close to the experimental results (determined by GC) of 90:5 (Figure 2). In comparison, the calculated n/iso selectivity of 1-propylene and 3,3-dimethyl-1-butene is 83:17, 95:5, respectively. Also, very close to the experimental observation: 90:10, 99:1. The linear selectivity of different substrate follows the sequence of : 3,3-dimethyl-1-butene > 1-propylene ≈ 1-octane.

Table S5 The free energy difference in the **TS1** transition states (ΔΔ*G*, kcal/mol) for the formation of linear and branch products from different substrates under M06/6-311+G(d) and B3LYP/6-311+G(d) level with toluene solvation effect and temperature correction, as well as the corresponding n/iso ratio.

Substrate	M062X/6-311+G(d)		B3LYP/6-311+G(d)		n/iso (Exp.)
	ΔΔ*G*-358-toluene	n/iso	ΔΔ*G*-358-toluene	n/iso	
1-octene	1.17	84:16	0.84	77:23	90/10
1-propylene	1.13	83:17	0.16	56:44	90/10
3,3-dimethyl-1-butene	2.04	95:5	1.43	88:12	>99/1

(3.2.5) Regeneration of pre-catalyst and active catalyst:

Based on our proposed mechanism, the anion intermediate $[Co(CO)_3]^-$ should come from the last nucleophile attack of benzyloxide, which comes from the deprotonation of benzyl alcohol by base [BnOH + NMPD = BnO^- + $[HNMPD]^+$], and $[Co(CO)_4]^-$ comes from CO coordination to $[Co(CO)_3]^-$. Subsequently, $[Co(CO)_3]^-$ and $[Co(CO)_4]^-$ can be further protonated to $HCo(CO)_3$ or $HCo(CO)_4$ by the conjugated acid $[HNMPD]^+$. Therefore, the reaction energies of these transformations have been computed (Table S6).

Since the reaction between $HCo(CO)_4$ and pyridine (Py) in THF solution has been reported to form protonated pyridinium ion (PyH^+) and free $[Co(CO)_4]^-$ [Eq. 1], we computed this reaction Gibbs free energy at first as benchmark. However, this reaction is computed to be endergonic and unfavorable thermodynamically (Table S6), on the contrary to the experimental finding (Fachinetti, G., Funaioli, T., Marcucci, M. J. *Organomet. Chem*. **1988**, *353*, 393-404), indicating that all these computational methods cannot reproduce the experimental results quantitatively and qualitatively, and the reason for such problem comes from the charge separation for free cation and anion.

In addition, the deprotonation of benzyl alcohol by pyridine has also been computed to be endergonic [Eq. 2]. The same is also for the deprotonation of benzyl alcohol by NMPD [Eq. 3]. Finally, the deprotonation of $HCo(CO)_4$ and $HCo(CO)_3$ by NMDP is also

endergonic (Eq. 4 and Eq. 5). Therefore, all these results cannot be used for qualitative and quantitative discussion and comparison.

$HCo(CO)_4 + Py = [PyH]^+ + [Co(CO)_4]^-$ (Eq. 1)

$BnOH + Py = BnO^- + [PyH]^+$ (Eq. 2)

$BnOH + NMPD = BnO^- + [HNMPD]^+$ (Eq. 3)

$HCo(CO)_4 + NMPD = [Co(CO)_4]^- + [HNMPD]^+$ (Eq. 4)

$HCo(CO)_3 + NMPD = [Co(CO)_3]^- + [HNMPD]^+$ (Eq. 5)

Table S6. Computed protonation Gibbs free energies (Δ*G*, kcal/mol) for Eq. 1-5

Unit kcal/mol	Δ*G*(298K, B3LYP/ 6-311+g(d))	Δ*G*(358K, B3LYP/6-311+g(d), toluene)	Δ*G*(358K, M062X/6-311+g(d), toluene)
**Eq. 1**	86.59	3.27[a]	23.25[a]
**Eq. 2**	141.63	47.44[a]	51.23[a]
**Eq. 3**	134.09	79.23	72.50
**Eq 4**	79.06	10.26	41.37
**Eq. 5**	96.26	28.96	57.70

a) Solvation effect of THF is considered for these reactions due to the experimental used solvent is THF (Fachinetti, G., Funaioli, T., Marcucci, M. J. *Organomet. Chem*. **1988**, *353*, 393-404).

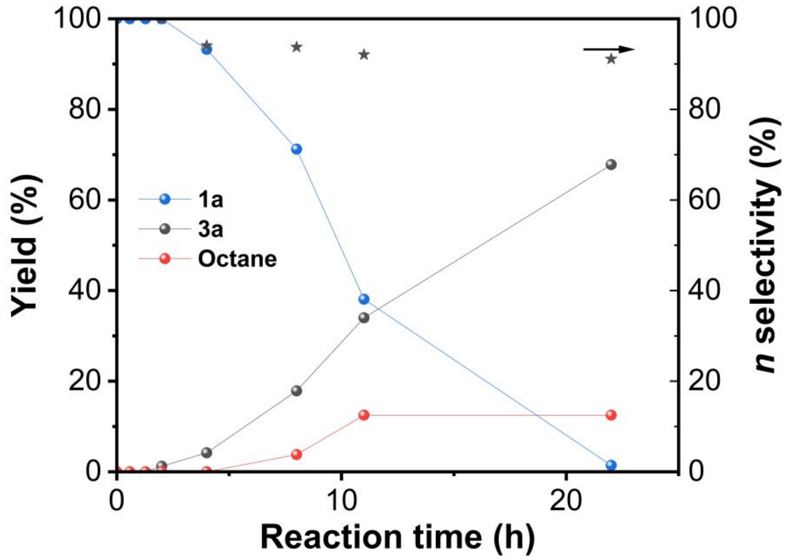

Figure S7. Time course profile showing the conversion of 1-octene, the yield of **3**, the yield of octane as a side product, and the *n* selectivity. Reaction conditions: 1-octene (0.2 mmol), benzyl alcohol (0.4 mmol), hexadecane (0.085 mmol), $Co(OAc)_2$ (3 mol%), **L2** (0.5 mol %), NMPD (20 mol%), toluene (2 mL), CO (2.0 bar), light (LED 390 nm), reaction temperature (60 ^oC).

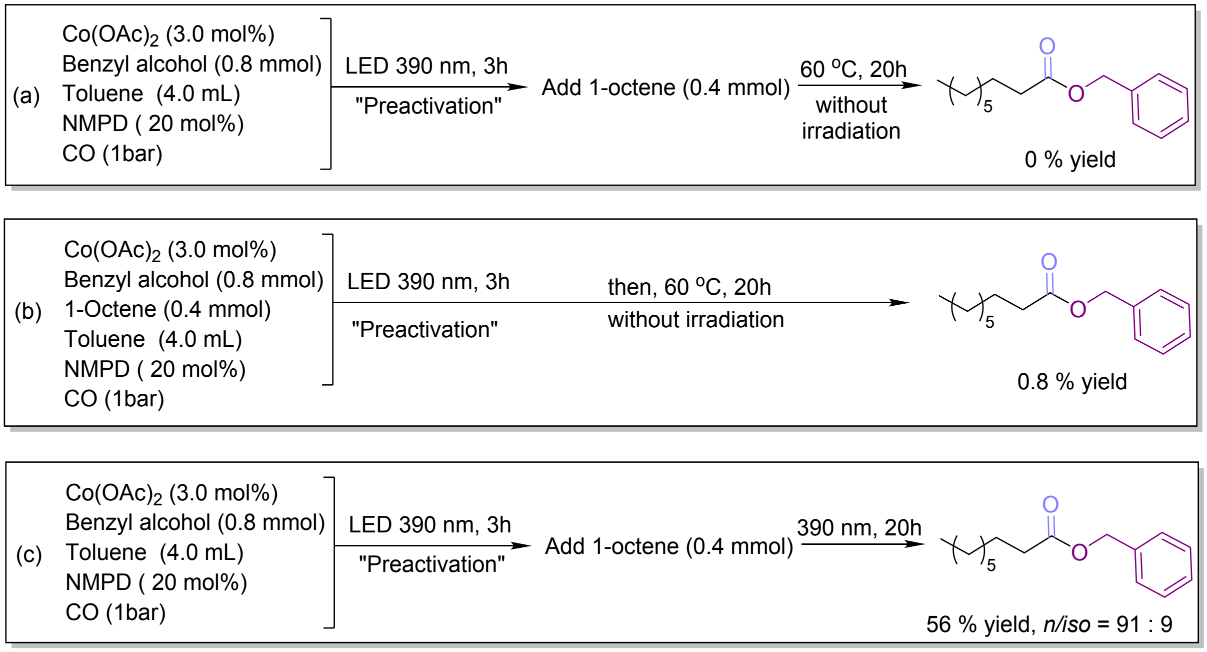

Scheme S1. (a) The reaction mixture in the absence of 1-octene was first irradiated for 3 h (catalyst pre-activation process), followed by the addition of 1-octene, and then reaction continued at 60 °C in the absence of light. No product was detected at the end of the reaction. (b) the reaction mixture was first irradiated for 3h (catalyst pre-activation process), and then reaction continued at 60 °C in the absence of light. 0.8 % yield of **3** during the first 3 h, and then no further increase in the absence of light. (c) The reaction mixture in the absence of 1-octene was first irradiated for 3 h (catalyst pre-activation process), followed by the addition of 1-octene, and then reaction continued with light irradiation. 56 % yield of **3** with *n/iso* ratio of 91 : 9.

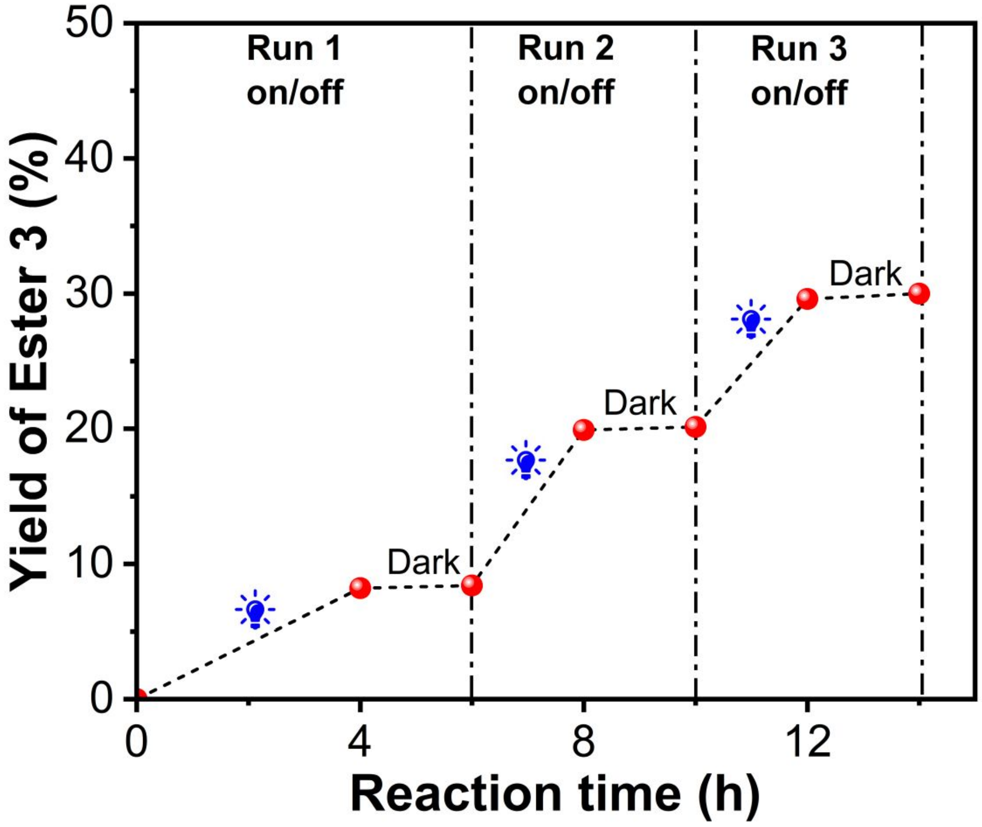

Figure S8. Light on/off experiment for the alkoxycarbonylation of 1-octene and benzyl alcohol. Reaction conditions: Reaction conditions: 1-octene (0.5 mmol), benzyl alcohol (1.0 mmol), hexadecane (0.17 mmol), $Co(OAc)_2$ (3 mol%), **L2** (0.5 mol %), NMPD (20 mol%), toluene (5 mL), CO (2.0 bar), light (LED 390 nm), reaction temperature (60 ^oC).

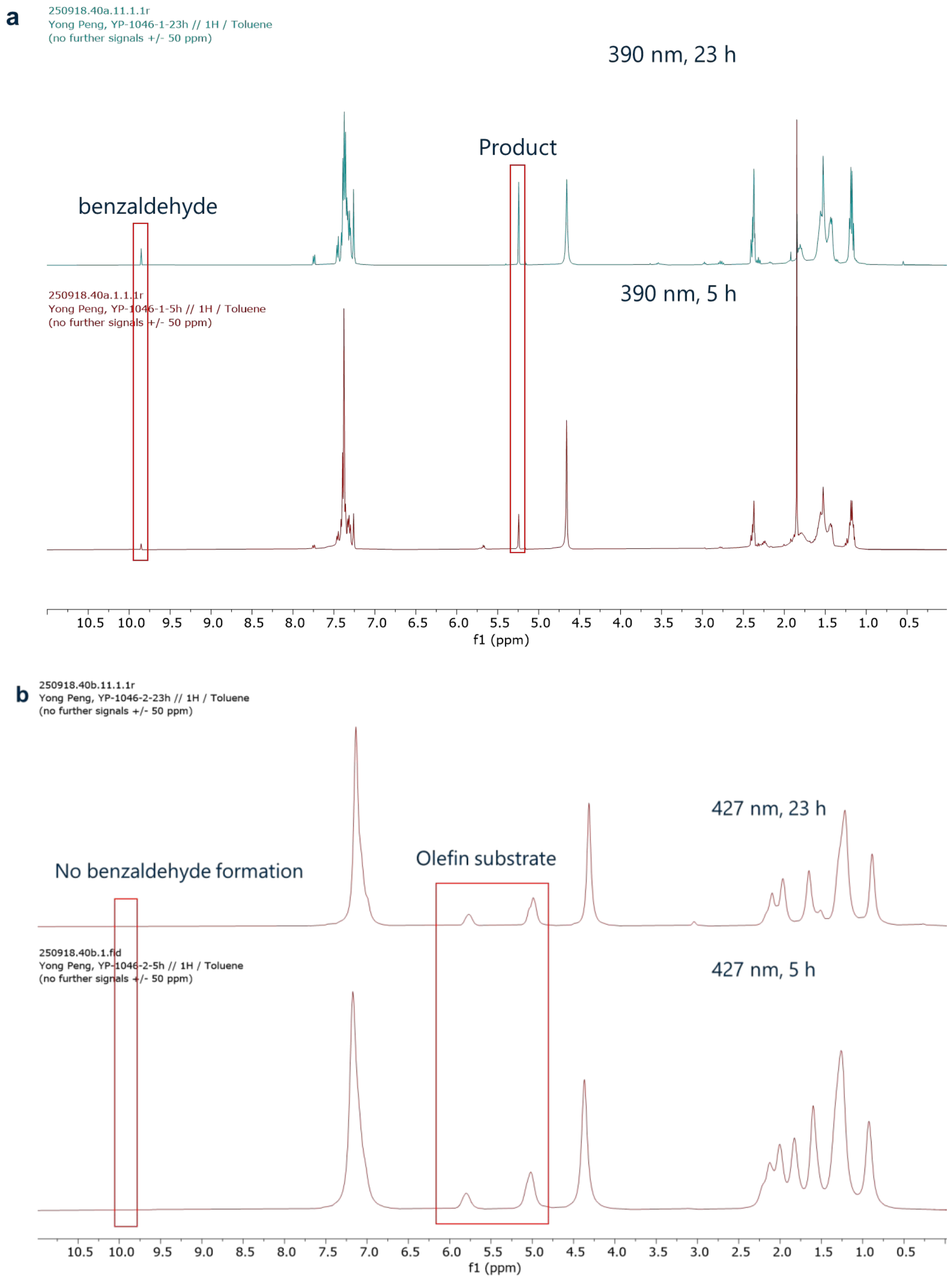

Figure S9. (a) ^{1}H NMR spectrum of the reaction mixture after 5 and 23 h irradiations with the 390 nm LED shows well-resolved signals, consistent with the reduction of the initially paramagnetic Co^{2+} pre-catalyst to a diamagnetic species under these conditions, in addition, the product formation and olefin substrate consumption were also observed. (b) the spectrum obtained after irradiation with the 427 nm LED displays broad and poorly resolved resonances, indicating Co^{2+} has not been reduced under this condition. Also, the substrate was not consumed under this condition. Reaction conditions: Toluene-D_8 3 mL, 1-octene 0.3 mmol, benzyl alcohol 0.6 mmol, $Co(OAc)_2$ 3 mol %, NMPD 20 mol %, CO 2 bar, reaction temperature (60 ^oC),.

a

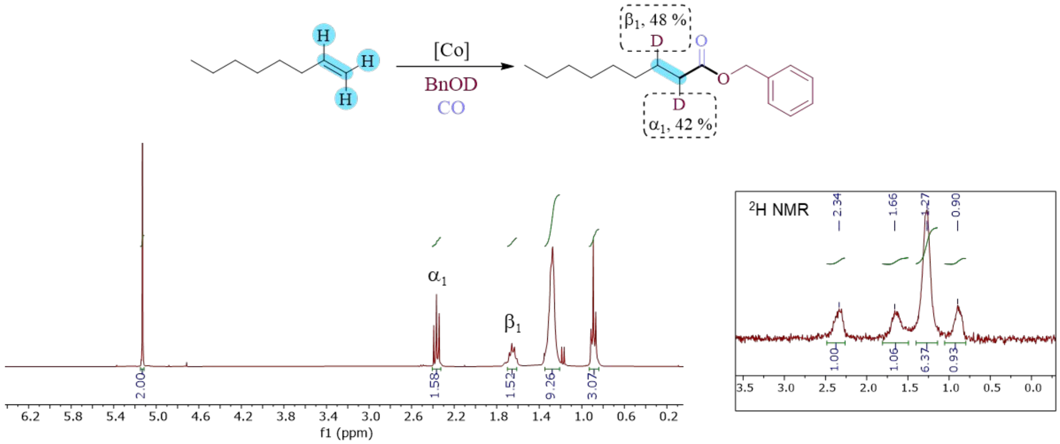

b

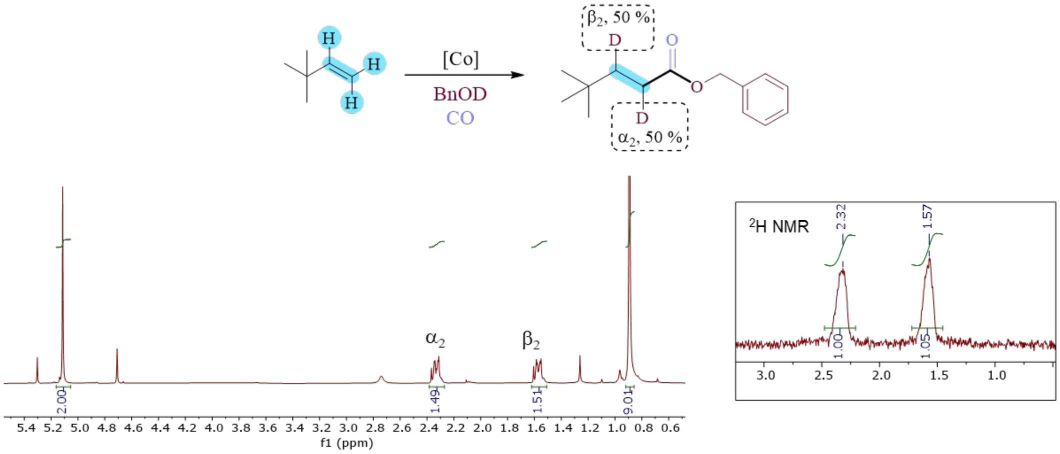

c

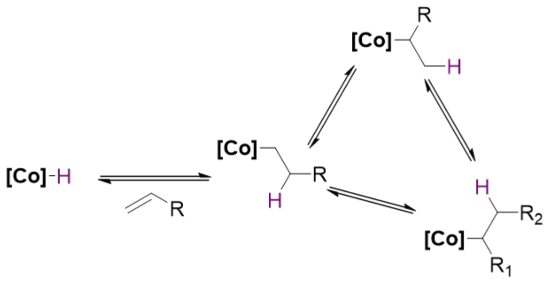

Figure S10. Isotope labeling experiment using (a) 1-octene and (b) 3,3-dimethyl-butene as the substrate. Reaction conditions: olefin (0.2 mmol), BnOD (0.4 mmol), $Co(OAc)_2$ (2 mol%), NMPD (20 mol%), toluene-D_8 (2 mL), CO (2.0 bar), light (LED 390 nm), reaction temperature (60 ^oC). It shows that the one deuterium atom from BnOD is incorporated into the double bond. Due to reversible β-hydride eliminating, the incorporated deuterium was scrambled between the α- and β- positions of the ester product, resulting in approximately 50 % deuteration at each site. The inset ^2H NMR figure clearly confirmed the deuteration of the final products, where 1-octene substrate shows deuterium signal across the alkyl chain, and the 3,3-dimethyl butene substrate shows deuterium signal only at the α and β positions relative to the ester carbonyl group. (c) demonstration the formation of different [Co]-alkyl intermediate species prior to the CO insertion step. The results indicate BnOD provide the protons, and the isomerization rate between olefin-[Co]-H and [Co]-alkyl is faster than the rate of CO insertion step.

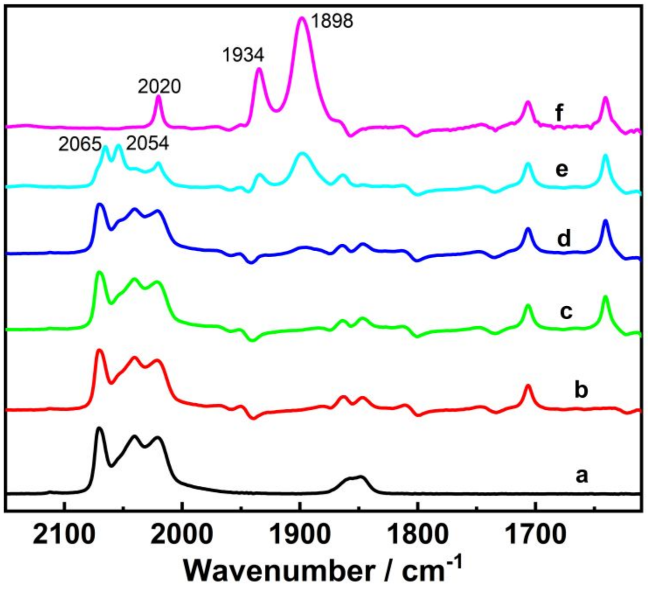

Figure S11. FTIR spectra of the catalyst system starting from $Co_2(CO)_8$. The spectra collected after the subsequential addition of the following reagent into 10 mL toluene: (a) the addition of 3 mol % of $Co_2(CO)_8$, (b) 2 mmol Benzyl alcohol (BnOH), (c) 1 mmol 1-octene and (d) 20 mol % of NMPD, (e) 1.3 bar of CO in the absence of light irradiation. (f) Spectrum collected after the reaction mixture irradiated for 20 hours using 390 nm LED lamp.

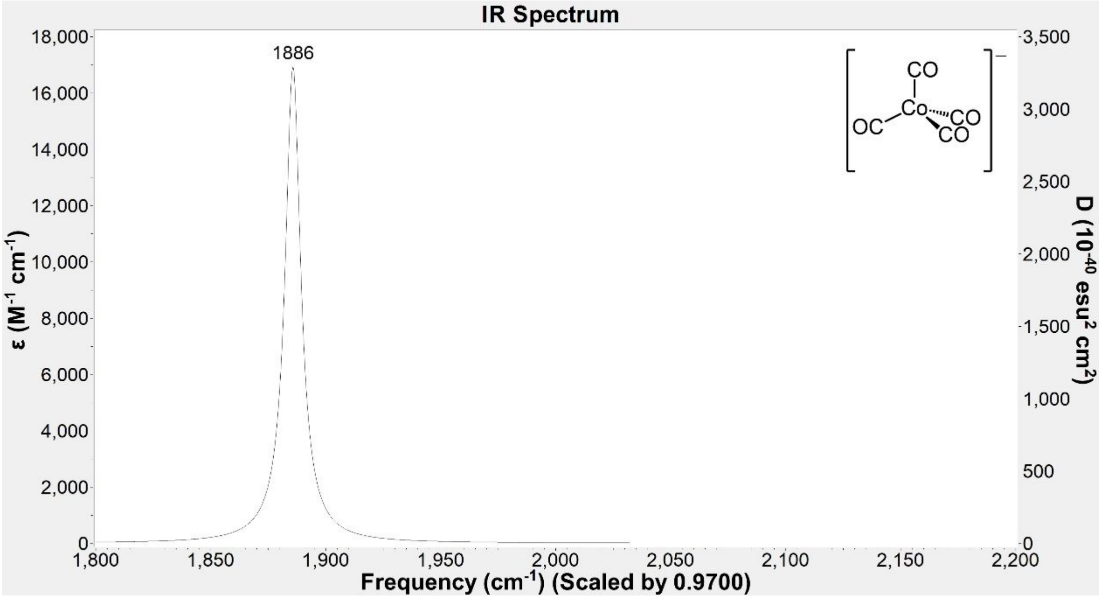

Figure S12. The IR spectra of $Co(CO)_4^-$ calculated under B3LYP/6-311+G(d) level, with molecular structures inserted in the upper right corner.

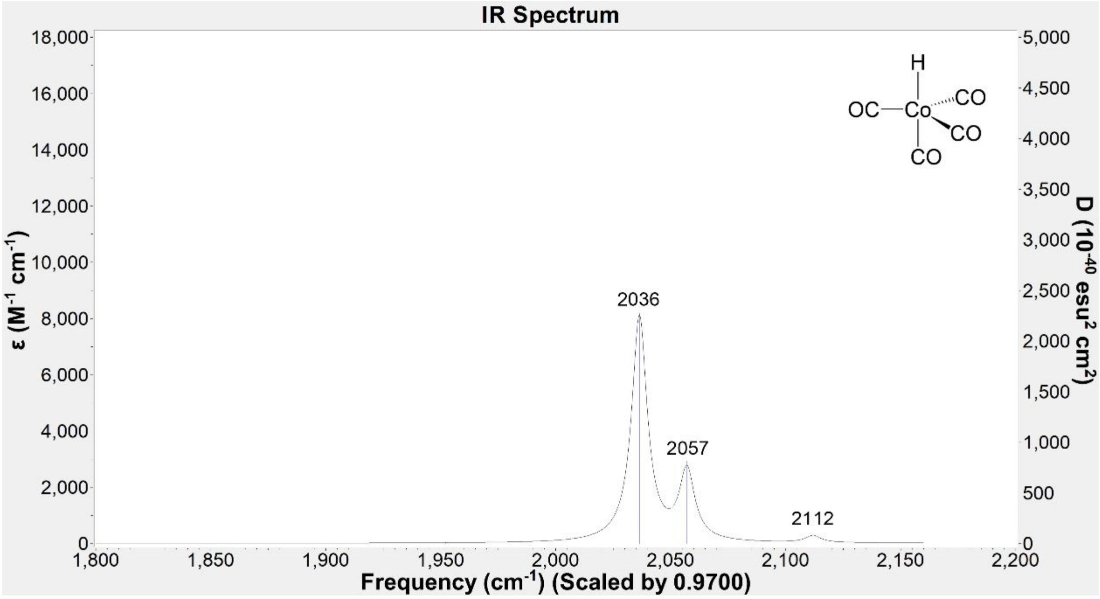

Figure S13. The IR spectra of $HCo(CO)_4$ calculated under B3LYP/6-311+G(d) level, with molecular structures inserted in the upper right corner.

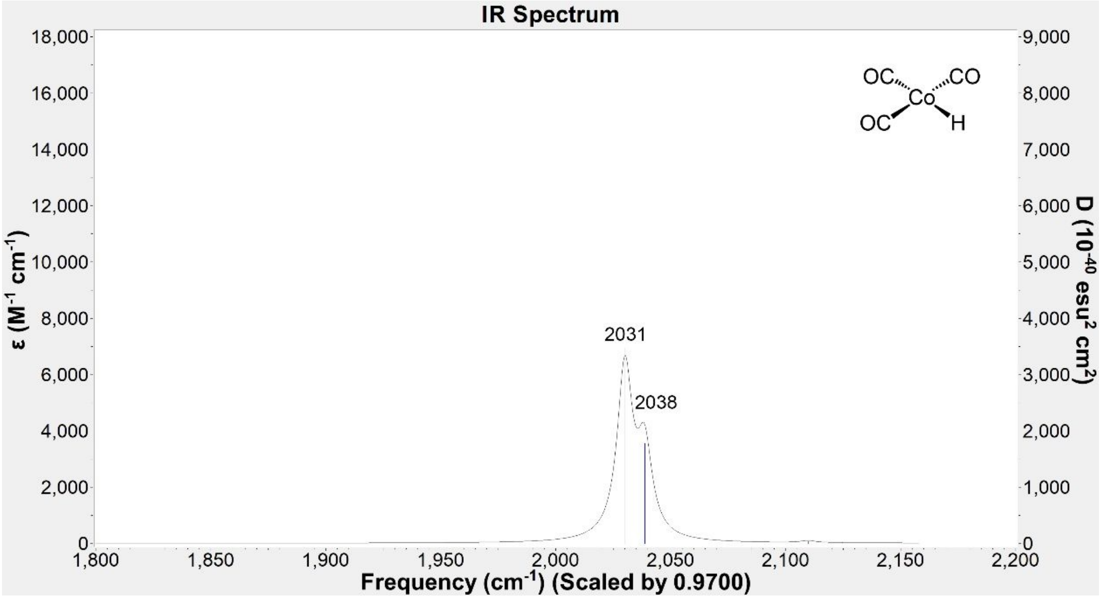

Figure S14. The IR spectra of $HCo(CO)_3$ calculated under B3LYP/6-311+G(d) level, with molecular structures inserted in the upper right corner.

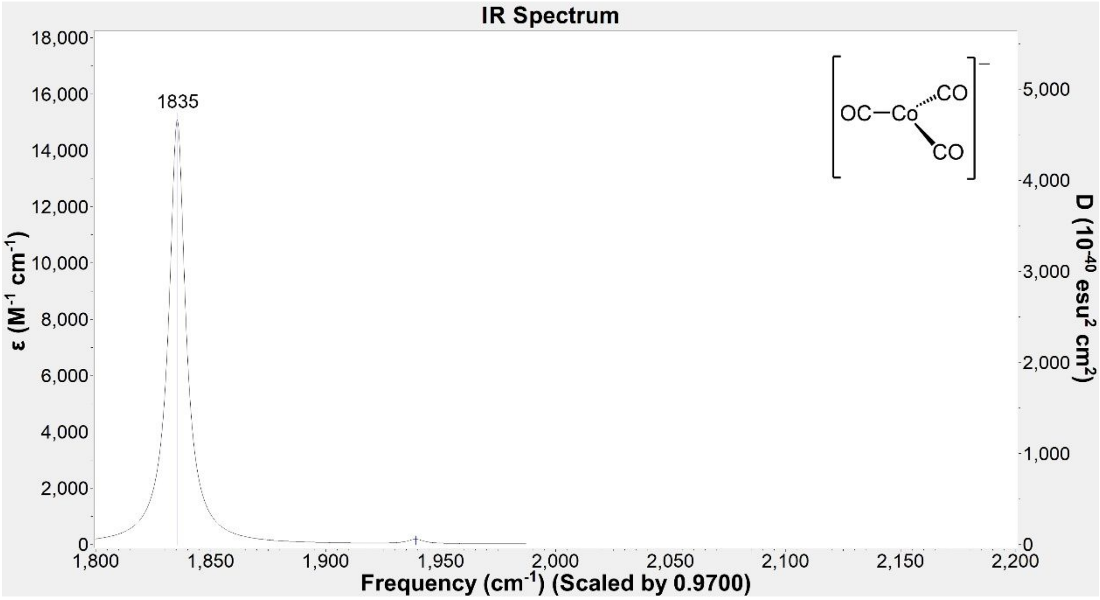

Figure S15. The IR spectra of $Co(CO)_3^-$ calculated under B3LYP/6-311+G(d) level, with molecular structures inserted in the upper right corner.

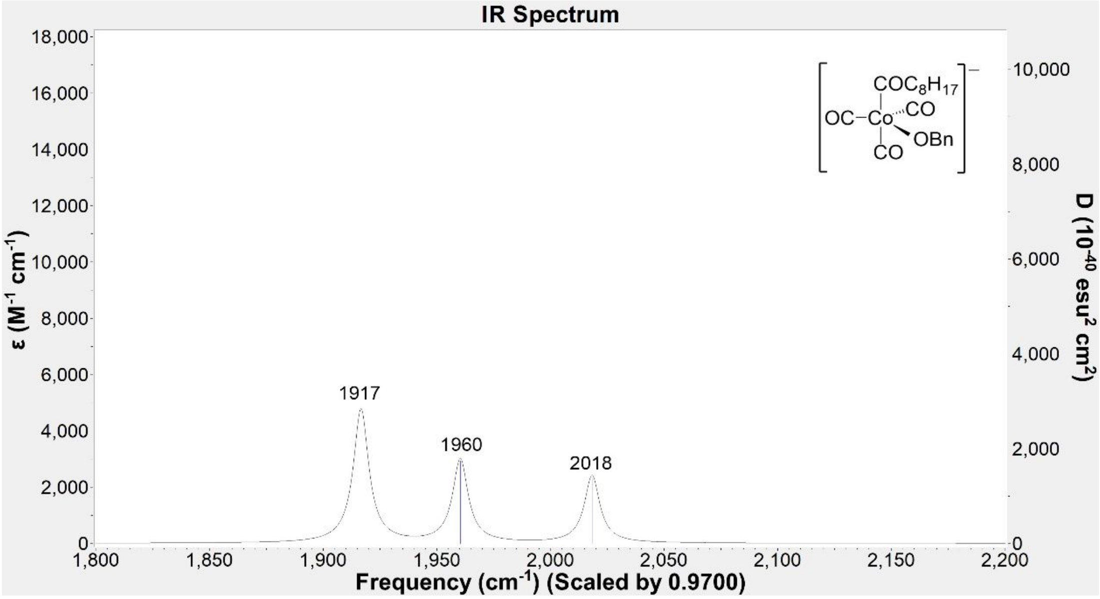

Figure S16. The IR spectra of $[Co(CO)_3(COC_8H_{17})(BnO)]^-$ calculated under B3LYP/6-311+G(d) level, with molecular structures inserted in the upper right corner.

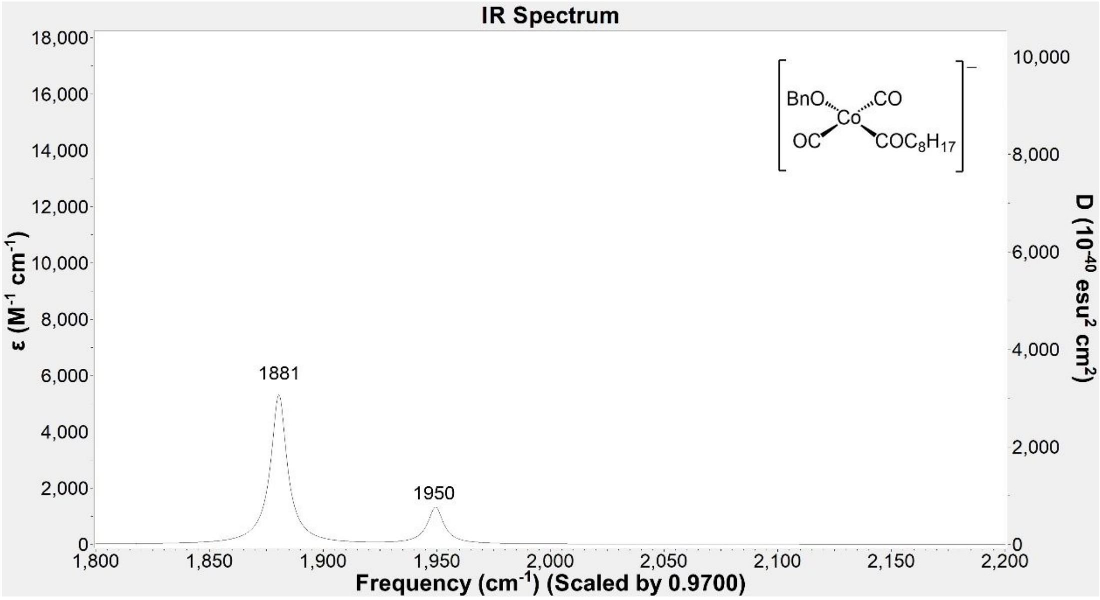

Figure S17. The IR spectra of $[Co(CO)_2(COC_8H_{17})(BnO)]^-$ calculated under B3LYP/6-311+G(d) level, with molecular structures inserted in the upper right corner.

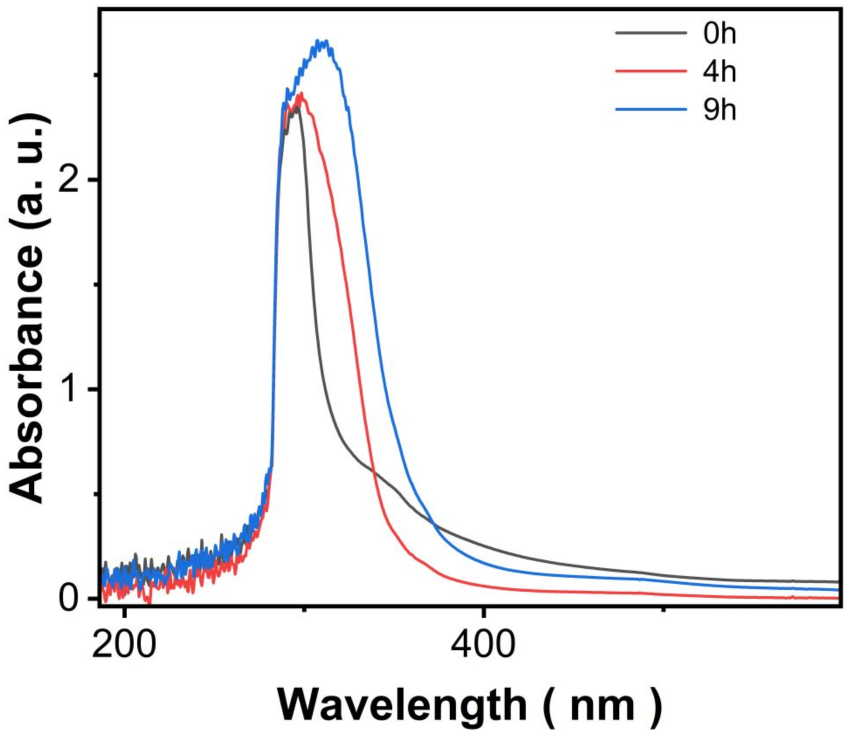

Figure S18. UV-vis spectra collected from solution after different reaction time. The measurement was conducted using a cuvette under argon, where the after-reaction solution was transferred with a syringe. Reaction conditions: Reaction conditions: Toluene 5 mL, 1-octene (0.5 mmol), benzyl alcohol (1 mmol, 2 equiv.), $Co(OAc)_2$ (3 mol %), NMPD (20 mol %), **L2** (0.5 mol %), CO (2 bar), reaction temperature (60 ^{o}C).

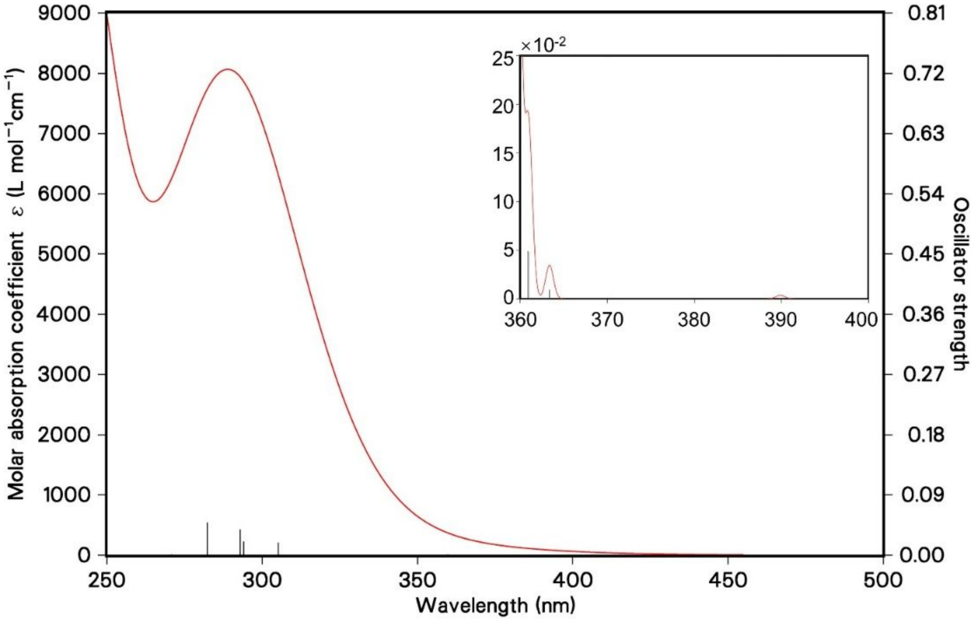

Figure S19. The calculated TD-DFT absorption spectra of $HCo(CO)_4$.

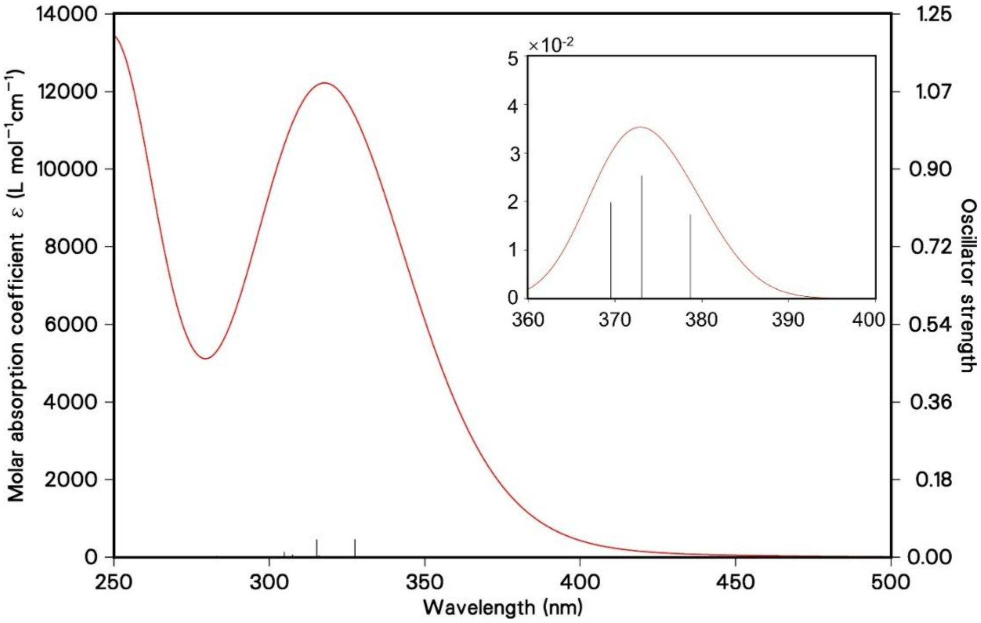

Figure S20. The calculated TD-DFT absorption spectra of $[Co(CO)_4]^{-}$.

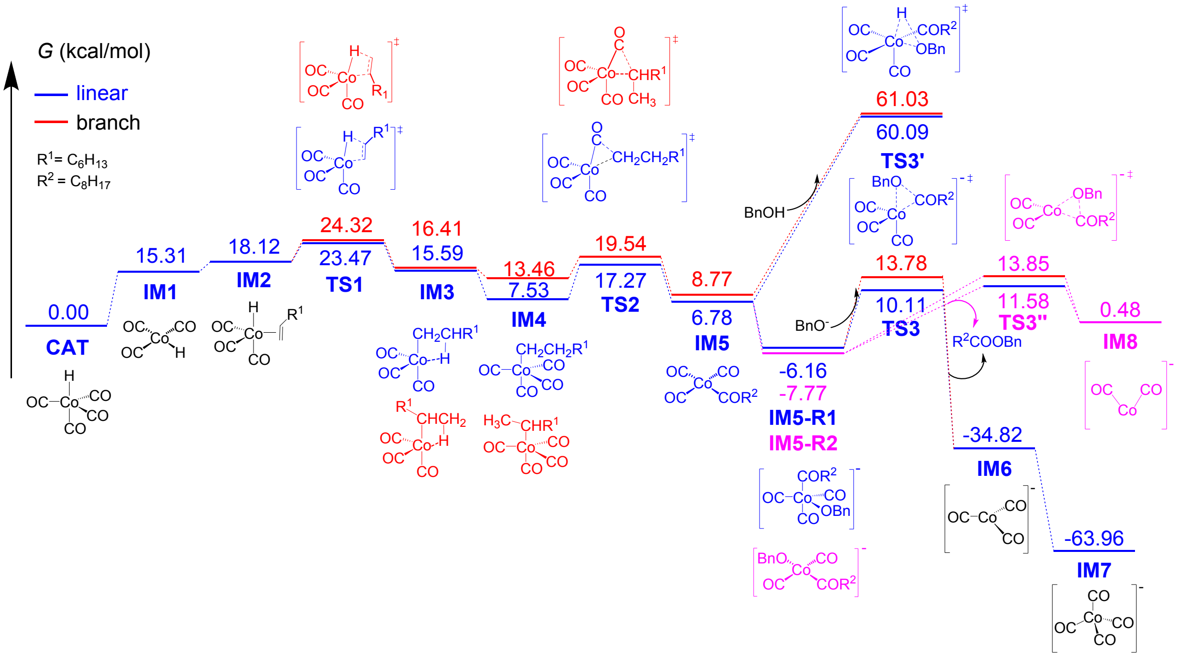

Figure S21. Full Gibbs free energy profiles of the catalytic cycle for 1-octene calculated at the B3LYP-SMD/6-311+G(d) level with toluene as solvent based on the B3LYP/6-311+G(d) optimized structures in gas phase along with thermal correction to Gibbs free energy at 358.15 K (CAT represents the corresponding catalyst, IM represents the intermediate during the reaction, and TS represents the transition state. The bule and red lines represent the pathways toward linear and branched esters, respectively.)

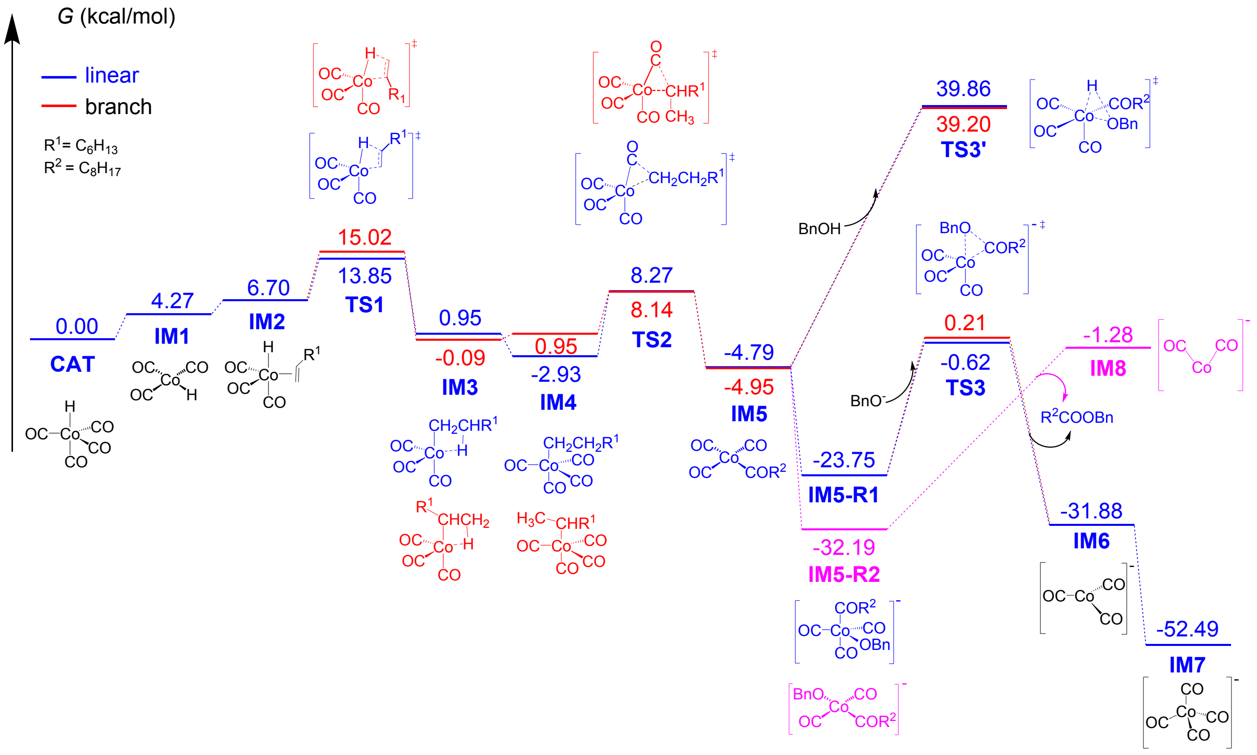

Figure S22. Full Gibbs free energy profiles of the catalytic cycle for 1-octene calculated at the M062X-SMD/6-311+G(d) level with toluene as solvent based on the M062X/6-311+G(d) optimized structures in gas phase along with thermal correction to Gibbs free energy at 358.15 K (CAT represents the corresponding catalyst, IM represents the intermediate during the reaction, and TS represents the transition state. The bule and red lines represent the pathways toward linear and branched esters, respectively.)

Table S7. Comparison of performance between the approaches developed in this work and that from the previous report.

**Feature**	**Alexanian**	**Present Manuscript**
Main Focus	Branched and cyclic olefins	Non-activated linear aliphatic olefins
Regioselectivity	Up to 63% for linear olefins (e.g., propylene)	Over 90% (up to 94%) for linear esters
Catalyst System	$Co_2(CO)_8$ with DMAP additive	Bench-stable $Co(OAc)_2$ with catalytic 1,3-dione ligands (e.g., **L2**)
Conditions	Mild, light-driven	Mild (60 °C, 2 bar CO), light-driven

3.3 Control experiment using $NaCo(CO)_4$ as the catalyst precursor.

$NaCo(CO)_4$ was prepared following the protocol from literature[1]. In a typical experiment, toluene 5 mL was mixed with 5 mg of $NaCo(CO)_4$, 0.5 mmol of 1-octene, 1 mmol of benzyl alcohol, 10 μL of NMPD under argon atmosphere using the reaction as shown in Figure S6. Afterwards, the system was charged with 2 bars of CO and kept stirring under the irradiation with 390nm LED lamp. The resulted ester product **3** was quantified with GC using hexadecane as the internal standard.

4. Characterizations of ester products

Benzyl nonanoate, 3

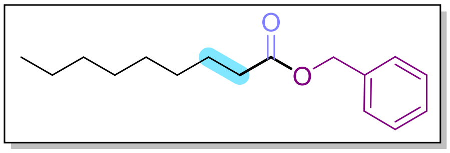

Yield 72 %, 35.8 mg , *n/iso* = 91 : 9, colorless oil. Large scale yield 67 %, 1.66 g.

^{1}H NMR (300 MHz, $CDCl_3$) δ 7.40 – 7.28 (m, 5H), 5.12 (s, 2H), 2.36 (t, *J* = 7.6 Hz, 2H), 1.72 – 1.57 (m, 2H), 1.39 – 1.20 (m, 10H), 0.88 (t, *J* = 6.8 Hz, 3H).

^{13}C NMR (75 MHz, $CDCl_3$) δ 173.8, 136.3, 128.7, 128.3, 66.2, 34.5, 31.9, 29.3, 29.3, 29.2, 25.1, 22.8, 14.2.

HRMS (EI): m/z calcd for $C_{16}H_{24}O_2^+$: 248.17708, Found: 248.17753 $[M]^+$.

Benzyl butyrate, 4

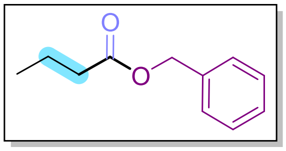

Yield 81 %, 144.2 mg, *n/iso* = 90 : 10, colorless oil.

^{1}H NMR (300 MHz, $CDCl_3$) δ 7.26 – 7.38 (m, 5H), 5.13 (s, 2H), 2.36 (t, *J* = 7.3 Hz, 2H), 1.80 – 1.60 (m, 2H), 0.97 (t, *J* = 7.4 Hz, 3H).

^{13}C NMR (75 MHz, $CDCl_3$) δ 173.5, 136.2, 128.6, 128.2, 66.1, 36.3, 18.4, 13.7.

HRMS (ESI): m/z calcd for $C_{11}H_{14}O_2Na_1^+$: 201.088601, found 201.0885618.

Benzyl heptanoate, 5

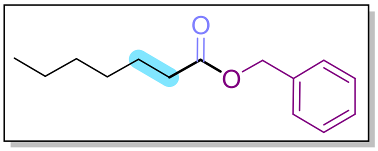

Yield 74 %, 32.6 mg, *n/iso* = 91 : 9, light yellow oil.

^{1}H NMR (300 MHz, CDCl$_3$) δ 7.40 – 7.29 (m, 5H), 5.12 (s, 2H), 2.36 (t, *J* = 7.5 Hz, 2H), 1.72 – 1.58 (m, 2H), 1.40 – 1.22 (m, 6H), 0.88 (t, *J* = 6.8 Hz, 3H).

^{13}C NMR (75 MHz, CDCl$_3$) δ 173.8, 136.3, 128.7, 128.3, 66.2, 34.5, 31.6, 28.9, 25.0, 22.6, 14.1.

HRMS(EI): m/z calcd for $C_{14}H_{20}O_2^+$: 220.14578, Found: 220.14583 [M]$^+$.

Benzyl dodecanoate, 6

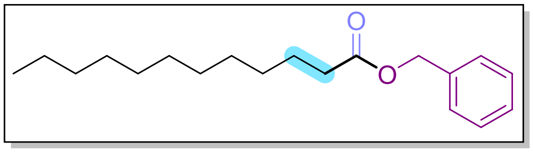

Yield 73 %, 42.3 mg, *n/iso* = 92 : 8, light yellow oil.

^{1}H NMR (400 MHz, CDCl$_3$) δ 7.40 – 7.29 (m, 5H), 5.12 (s, 2H), 2.36 (t, *J* = 5.7 Hz, 2H), 1.71 – 1.59 (m, 2H), 1.37 – 1.21 (m, 16H), 0.89 (t, *J* = 5.2 Hz, 3H).

^{13}C NMR (101 MHz, CDCl$_3$) δ 173.7, 136.2, 128.6, 128.2, 128.2, 66.1, 34.4, 31.9, 29.6, 29.6, 29.5, 29.4, 29.3, 29.2, 25.0, 22.7, 14.1.

HRMS(EI): m/z calcd for $C_{19}H_{30}O_2^+$: 290.22403, Found: 290.22485 [M]$^+$.

Benzyl tridecanoate, 7

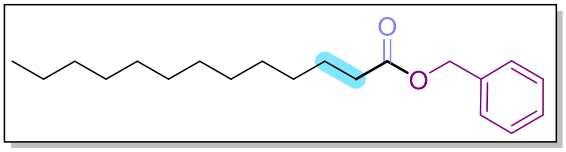

Yield 73 %, 44.4 mg, *n/iso* = 92 : 8, light yellow oil.

^{1}H NMR (300 MHz, CDCl$_3$) δ 7.39 – 7.28 (m, 5H), 5.12 (s, 2H), 2.36 (t, *J* = 7.5 Hz, 2H), 1.73 – 1.57 (m, 2H), 1.40 – 1.19 (m, 18H), 0.89 (t, *J* = 6.7 Hz, 3H).

^{13}C NMR (75 MHz, CDCl$_3$) δ 173.8, 136.3, 128.7, 128.6, 128.3, 128.3, 66.2, 34.5, 32.0, 29.8, 29.8, 29.7, 29.6, 29.5, 29.4, 29.3, 25.1, 22.8, 14.2.

HRMS(EI): m/z calcd for $C_{20}H_{32}O_2^+$: 304.23968, Found: 304.24110 [M]$^+$.

Benzyl pentadecanoate, 8

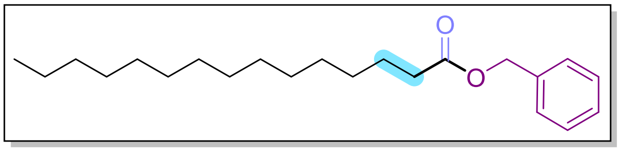

Yield 70 %, 46.5 mg, *n/iso* = 92 : 8, light yellow oil.

^{1}H NMR (300 MHz, $CDCl_3$) δ 7.40 – 7.28 (m, 5H), 5.12 (s, 2H), 2.36 (t, *J* = 7.5 Hz, 2H), 1.72 – 1.58 (m, 2H), 1.36 – 1.20 (m, 22H), 0.93 – 0.82 (m, 3H).

^{13}C NMR (75 MHz, $CDCl_3$) δ 173.8, 136.3, 128.7, 128.3, 66.2, 34.5, 32.1, 29.8, 29.8, 29.7, 29.6, 29.5, 29.4, 29.3, 25.1, 22.8, 14.3.

HRMS(EI): m/z calcd for $C_{22}H_{36}O_2^+$: 332.27098, Found: 332.27187 $[M]^+$.

Benzyl 4,4-dimethylpentanoate, 9

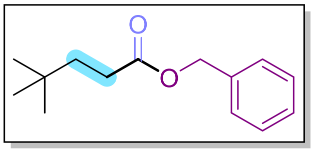

Yield 85 %, 37.5 mg, *n/iso* > 99.0 : 1, light yellow oil.

^{1}H NMR (300 MHz, $CDCl_3$) δ 7.40 – 7.31 (m, 5H), 5.12 (s, 2H), 2.42 – 2.29 (m, 2H), 1.66 – 1.53 (m, 2H), 0.90 (s, 9H).

^{13}C NMR (75 MHz, $CDCl_3$) δ 174.4, 136.2, 128.7, 128.4, 128.3, 66.3, 38.6, 30.2, 30.2, 29.1.

HRMS (EI): m/z calcd for $C_{14}H_{20}O_2^+$: 220.14578, Found: 220.14593 $[M]^+$.

Benzyl 3,5,5-trimethylhexanoate, 10

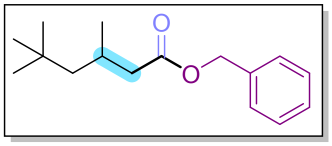

Yield 93 %, 46.4 mg, *n/iso* > 99 : 1, light yellow oil.

^{1}H NMR (300 MHz, $CDCl_3$) δ 7.39 – 7.30 (m, 5H), 5.12 (d, *J* = 1.0 Hz, 2H), 2.36 (dd, *J* = 14.5, 6.1 Hz, 1H), 2.19 (dd, *J* = 14.5, 7.9 Hz, 1H), 2.13 – 2.02 (m, 1H), 1.26 (dd, *J* = 14.0, 4.0 Hz, 1H), 1.12 (dd, *J* = 14.1, 6.3 Hz, 1H), 0.99 (d, *J* = 6.6 Hz, 3H), 0.90 (s, 9H).

^{13}C NMR (75 MHz, CDCl$_3$) δ 173.1, 136.3, 128.6, 128.4, 128.3, 66.1, 50.6, 44.1, 31.2, 30.1, 27.2, 22.8.

HRMS(EI): m/z calcd for $C_{16}H_{24}O_2^+$: 248.17708, Found: 248.17762 [M]$^+$.

Benzyl 2-((1S,5S)-6,6-dimethylbicyclo[3.1.1]heptan-2-yl)acetate, 11

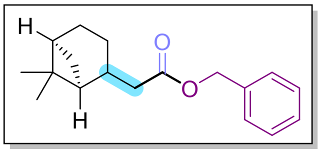

Yield 45 %, 24.6 mg, *n/iso* > 99 : 1, light yellow oil.

^{1}H NMR (400 MHz, CDCl$_3$) δ 7.43 – 7.29 (m, 5H), 5.14 (d, *J* = 12.4 Hz, 1H), 5.08 (d, *J* = 12.3 Hz, 1H), 2.55 – 2.43 (m, 1H), 2.34 – 2.20 (m, 2H), 2.10 – 2.00 (m, 1H), 1.92 – 1.84 (m, 1H), 1.82 – 1.67 (m, 4H), 1.38 – 1.24 (m, 2H), 1.19 (s, 3H), 0.85 (s, 3H).

^{13}C NMR (101 MHz, CDCl$_3$) δ 173.1, 136.3, 128.7, 128.3, 128.3, 66.1, 45.6, 41.1, 40.7, 39.6, 32.3, 26.8, 24.4, 23.3, 21.7, 20.2.

HRMS(ESI): m/z Calc. for $C_{18}H_2O_2Na^+$: 295.1674, Found: 295.1684

Benzyl cyclohexanecarboxylate, 16

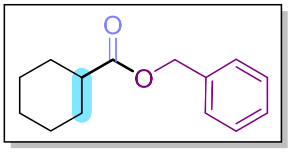

Yield 61 %, 26.6 mg, light yellow oil.

^{1}H NMR (300 MHz, CDCl$_3$) δ 7.40 – 7.28 (m, 5H), 5.12 (s, 2H), 2.36 (tt, *J* = 11.3, 3.6 Hz, 1H), 2.01 – 1.88 (m, 2H), 1.82 – 1.70 (m, 2H), 1.69 – 1.58 (m, 1H), 1.56 – 1.39 (m, 2H), 1.36 – 1.21 (m, 3H).

^{13}C NMR (75 MHz, CDCl$_3$) δ 176.0, 136.5, 128.6, 128.2, 128.1, 66.0, 43.3, 29.1, 25.9, 25.6.

HRMS(EI): m/z calcd for $C_{14}H_{18}O_2^+$: 218.13013, Found: 218.13011 [M]$^+$.

Benzyl 3,5-dimethylhexanoate, 18

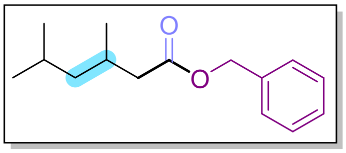

Yield 71 %, 33.2 mg, *n/iso* > 99 : 1, light yellow oil.

^{1}H NMR (300 MHz, $CDCl_3$) δ 7.38 – 7.30 (m, 5H), 5.12 (s, 2H), 2.33 (dd, *J* = 14.4, 5.8 Hz, 1H), 2.25 – 1.99 (m, 2H), 1.64 – 1.44 (m, 1H), 1.21 – 0.97 (m, 2H), 0.92 (d, *J* = 6.5 Hz, 3H), 0.89 – 0.83 (m, 6H).

^{13}C NMR (75 MHz, $CDCl_3$) δ 173.3, 136.3, 128.7, 128.4, 128.3, 66.1, 46.3, 42.3, 28.3, 25.3, 23.4, 22.2, 20.0.

HRMS(EI): m/z calcd for $C_{15}H_{22}O_2^+$: 234.16143, Found: 234.16187 $[M]^+$.

Benzyl 3,6-dimethylheptanoate, 19

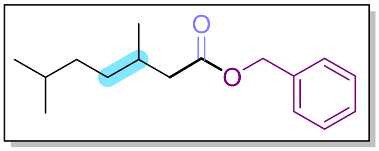

Yield 63 %, 31.0 mg, *n/iso* > 99 : 1, light yellow oil.

^{1}H NMR (300 MHz, $CDCl_3$) δ 7.42 – 7.29 (m, 5H), 5.12 (s, 2H), 2.36 (dd, *J* = 14.7, 6.2 Hz, 1H), 2.17 (dd, *J* = 14.7, 8.0 Hz, 1H), 2.07 – 1.82 (m, 1H), 1.48 (m, 1H), 1.38 – 1.02 (m, 4H), 0.93 (d, *J* = 6.7 Hz, 3H), 0.87 (d, *J* = 1.4 Hz, 3H), 0.84 (d, *J* = 1.3 Hz, 3H).

^{13}C NMR (75 MHz, $CDCl_3$) δ 173.3, 136.3, 128.7, 128.4, 128.3, 66.1, 42.0, 36.3, 34.6, 30.8, 28.3, 22.9, 22.6, 19.9.

HRMS(EI): m/z calcd for $C_{16}H_{24}O_2^+$: 248.17708, Found: 248.17775 $[M]^+$.

Benzyl 3-cyclohexylpropanoate, 20

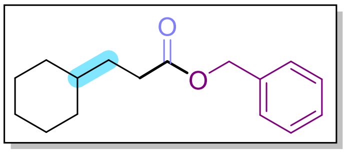

Yield 58 %, 28.7 mg, *n/iso* = 90 : 10, light yellow oil.

^{1}H NMR (300 MHz, $CDCl_3$) δ 7.39 – 7.30 (m, 5H), 5.12 (s, 2H), 2.41 – 2.33 (m, 2H), 1.75 – 1.61 (m, 5H), 1.60 – 1.49 (m, 2H), 1.29 – 1.10 (m, 4H), 0.98 – 0.81 (m, 2H).

^{13}C NMR (75 MHz, CDCl$_3$) δ 174.1, 136.3, 128.7, 128.3, 128.3, 66.2, 37.3, 33.1, 32.4, 32.0, 26.6, 26.3.

HRMS(EI): m/z calcd for $C_{16}H_{22}O_2^+$: 246.16143, Found: 246.16131 [M]$^+$.

Benzyl 5-methylhexanoate 21-1 (s$_1$), Bbenzyl 3-methylhexanoate 21-2 (s$_2$),

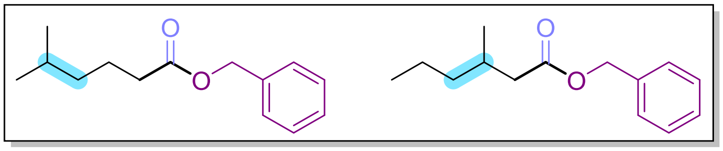

Yield 58 %, 25.5 mg, 3s$_1$: 3s$_2$ = 1.5 : 1, n/*iso* > 99.0 : 1, light yellow oil.

^{1}H NMR (300 MHz, CDCl$_3$) δ 7.41 – 7.28 (m, 5H, **s$_1$&s$_2$**), 5.12 (s, 2H, **s$_1$&s$_2$**), 2.42 – 2.29 (m, 1.64H, 1.22H****s$_1$**, 0.42H****s$_2$**), 2.17 (dd, *J* = 14.7, 8.0 Hz, 0.42H****s$_2$**), 2.09 – 1.90 (m, 0.42H****s$_2$**), 1.72 – 1.47 (m, 1.83H****s$_1$**), 1.40 – 1.07 (m, 3.52H, 1.68H****s$_1$**, 1.83H****s$_2$**), 0.93 (d, *J* = 6.6 Hz, 1.26H****s$_2$**), 0.91 – 0.85 (m, 4.92H, 3.66H****s$_1$**, 1.26H****s$_2$**).

^{13}C NMR (75 MHz, CDCl$_3$) δ 173.8 (**s$_1$**), 173.3 (**s$_2$**), 136.3 (**s$_1$&s$_2$**), 128.7 (**s$_1$&s$_2$**), 128.3 (**s$_1$&s$_2$**), 128.3 (**s$_1$&s$_2$**), 66.2 (**s$_1$**), 66.1 (**s$_2$**), 42.0 (**s$_2$**), 39.1 (**s$_2$**), 38.5 (**s$_1$**), 34.7 (**s$_1$**), 30.3 (**s$_2$**), 27.9 (**s$_1$**), 23.0 (**s$_1$**), 22.6 (**s$_1$**), 20.1 (**s$_2$**), 19.8 (**s$_2$**), 14.3 (**s$_2$**).

HRMS(EI): m/z calcd for $C_{14}H_{20}O_2^+$: 220.14578, Found: 220.14608 [M]$^+$.

4-Fluorobenzyl nonanoate, 22

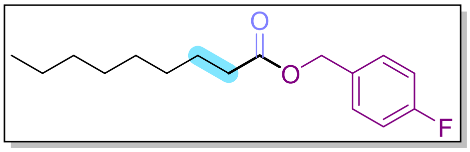

Yield 81 %, 43.1 mg, *n/iso* = 88 : 12, light yellow oil.

^{1}H NMR (400 MHz, CDCl$_3$) δ 7.34 – 7.24 (m, 2H), 7.05 – 6.94 (m, 2H), 5.03 (s, 2H), 2.29 (t, *J* = 7.5 Hz, 2H), 1.66 – 1.52 (m, 2H), 1.32 – 1.12 (m, 10H), 0.89 – 0.78 (t, *J* = 6.9 Hz, 3H).

^{13}C NMR (101 MHz, CDCl$_3$) δ 173.8, 162.7 (d, *J* = 246.7 Hz), 132.1 (d, *J* = 3.2 Hz), 130.3 (d, *J* = 8.1 Hz), 115.6 (d, *J* = 21.5 Hz), 65.5, 34.4, 31.9, 29.3, 29.2, 25.1, 22.8, 14.2.

^{19}F NMR (376 MHz, CDCl$_3$) δ -113.75 – -113.86 (m).

HRMS(EI): m/z calcd for $C_{16}H_{23}O_2F_1^+$: 266.16766, Found: 266.16763 [M]$^+$.

4-Chlorobenzyl nonanoate, 23

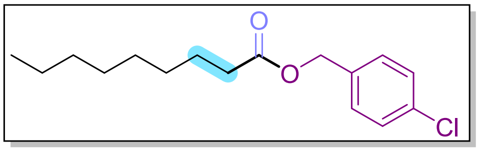

Yield 67 %, 38.0 mg, *n/iso* = 87 : 13, light yellow oil.

^{1}H NMR (400 MHz, $CDCl_3$) δ 7.36 – 7.25 (m, 4H), 5.07 (s, 2H), 2.34 (t, *J* = 7.6 Hz, 2H), 1.71 – 1.55 (m, 2H), 1.36 – 1.19 (m, 10H), 0.87 (t, *J* = 6.9 Hz, 3H).

^{13}C NMR (101 MHz, $CDCl_3$) δ 173.7, 134.8, 134.2, 129.7, 128.8, 65.3, 34.4, 31.9, 29.3, 29.2, 25.1, 22.8, 14.2.

HRMS(EI): m/z calcd for $C_{16}H_{23}O_2{}^{37}Cl_1{}^+$: 284.13516, Found: 284.13423 $[M]^+$.

4-Bromobenzyl nonanoate, 24

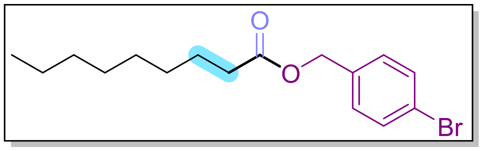

Yield 58 %, 37.8 mg, *n/iso* = 87 : 13, light brown oil.

^{1}H NMR (400 MHz, $CDCl_3$) δ 7.51 – 7.46 (m, 2H), 7.25 – 7.20 (m, 2H), 5.05 (s, 2H), 2.34 (t, *J* = 7.5 Hz, 2H), 1.71 – 1.57 (m, 2H), 1.36 – 1.19 (m, 10H), 0.87 (t, *J* = 6.9 Hz, 3H).

^{13}C NMR (101 MHz, $CDCl_3$) δ 173.7, 135.3, 131.8, 130.0, 122.3, 65.4, 34.4, 31.9, 29.3, 29.2, 25.1, 22.8, 14.2.

HRMS(EI): m/z calcd for $C_{16}H_{23}O_2Br_1{}^+$: 326.08759, Found: 326.08813 $[M]^+$.

4-(Trifluoromethyl)benzyl nonanoate, 25

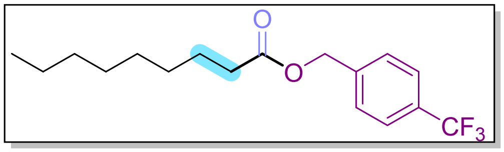

Yield 54 %, 34.0 mg, *n/iso* = 90 : 10, light yellow oil.

^{1}H NMR (400 MHz, $CDCl_3$) δ 7.55 (d, *J* = 8.1 Hz, 2H), 7.39 (d, *J* = 8.0 Hz, 2H), 5.09 (s, 2H), 2.30 (t, *J* = 7.5 Hz, 2H), 1.68 – 1.48 (m, 2H), 1.36 – 1.01 (m, 10H), 0.83 – 0.77 (t, *J* = 6.9 Hz, 3H).

^{13}C NMR (101 MHz, $CDCl_3$) δ 173.6, 140.3, 130.4 (q, J = 32.4 Hz), 125.6 (q, J = 3.7 Hz), 124.2 (q, J = 272.0 Hz), 65.2, 34.4, 31.9, 29.3, 29.3, 25.1, 22.8, 14.2.

^{19}F NMR (376 MHz, $CDCl_3$) δ -62.65.

HRMS(EI):m/z calcd for $C_{17}H_{23}O_2F_3^+$: 316.16447, Found: 316.16436 $[M]^+$.

4-Methylbenzyl nonanoate, 26

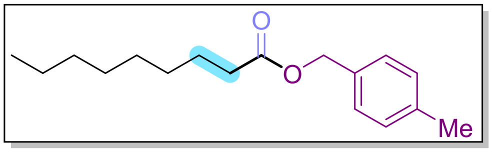

Yield 63 %, 33.0 mg, *n/iso* = 89 : 11, light yellow oil.

^{1}H NMR (400 MHz, $CDCl_3$) δ 7.25 (d, J = 7.9 Hz, 2H), 7.17 (d, J = 7.9 Hz, 2H), 5.08 (s, 2H), 2.39 – 2.29 (m, 5H), 1.70 – 1.58 (m, 2H), 1.37 – 1.18 (m, 10H), 0.88 (t, J = 6.9, 3H).

^{13}C NMR (101 MHz, $CDCl_3$) δ 173.9, 138.1, 133.3, 129.3, 128.5, 66.1, 34.5, 31.9, 29.3, 29.3, 29.2, 25.1, 22.8, 21.3, 14.2.

HRMS(EI): m/z calcd for $C_{17}H_{26}O_2^+$: 262.19273, Found: 262.19290 $[M]^+$

4-(tert-butyl)benzyl nonanoate, 27

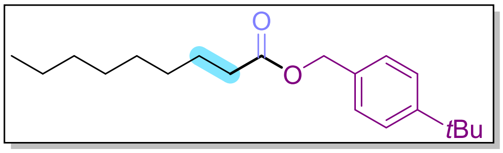

Yield 78 %, 47.4 mg, *n/iso* = 93 : 7, light yellow oil.

^{1}H NMR (400 MHz, $CDCl_3$) δ 7.43 – 7.37 (m, 2H), 7.33 – 7.27 (m, 2H), 5.10 (s, 2H), 2.35 (t, J = 7.5 Hz, 2H), 1.72 – 1.58 (m, 2H), 1.36 – 1.21 (m, 19H), 0.88 (t, J = 6.9 Hz, 3H).

^{13}C NMR (101 MHz, $CDCl_3$) δ 173.9, 151.3, 133.2, 128.2, 125.6, 66.1, 34.7, 34.5, 31.9, 31.4, 29.4, 29.3, 29.3, 25.1, 22.8, 14.2.

HRMS(ESI): m/z calcd for $C_{20}H_{32}O_2Na^+$: 327.2300, Found: 327.2309.

4-Methoxybenzyl nonanoate, 28

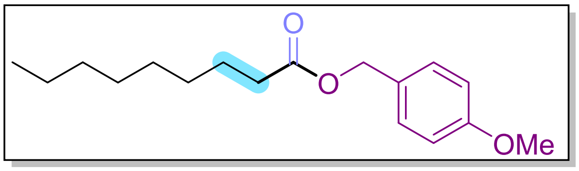

Yield 47 %, 26.1 mg, *n/iso* = 89 : 11, light yellow oil.

^{1}H NMR (400 MHz, $CDCl_3$) δ 7.32 – 7.27 (m, 2H), 6.91 – 6.86 (m, 2H), 5.05 (s, 2H), 3.81 (s, 3H), 2.32 (t, *J* = 7.6 Hz, 2H), 1.69 – 1.57 (m, 2H), 1.34 – 1.20 (m, 10H), 0.88 (t, *J* = 6.9 Hz, 3H).

^{13}C NMR (101 MHz, $CDCl_3$) δ 173.9, 159.7, 130.2, 128.4, 114.0, 66.0, 55.4, 34.5, 31.9, 29.3, 29.3, 25.1, 22.8, 14.2.

HRMS (EI): m/z calcd for $C1_7H_{26}O_3^+$: 278.18765, Found: 278.18685 $[M]^+$.

[1,1'-Biphenyl]-4-ylmethyl nonanoate, 29

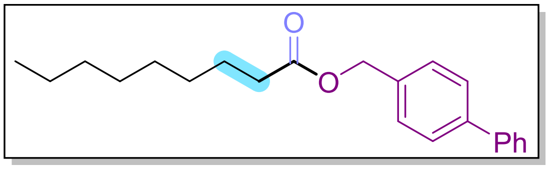

Yield 52 %, 33.7 mg, *n/iso* = 90 : 10, white solid, m. p., 38.1-39.1 ^{o}C

^{1}H NMR (300 MHz, $CDCl_3$) δ 7.63 – 7.56 (m, 4H), 7.49 – 7.41 (m, 4H), 7.40 – 7.32 (m, 1H), 5.16 (s, 2H), 2.38 (t, *J* = 7.5 Hz, 2H), 1.75 – 1.59 (m, 2H), 1.39 – 1.21 (m, 10H), 0.8 (t, *J* = 6.8 Hz, 3H).

^{13}C NMR (75 MHz, $CDCl_3$) δ 173.9, 141.3, 140.8, 135.3, 128.9, 128.8, 127.6, 127.4, 127.3, 66.0, 34.5, 31.9, 29.4, 29.3, 29.3, 25.1, 22.8, 14.2.

HRMS(EI): m/z calcd for $C_{22}H_{28}O_2^+$: 324.20838, Found: 324.20852 $[M]^+$.

2-Chlorobenzyl nonanoate, 30

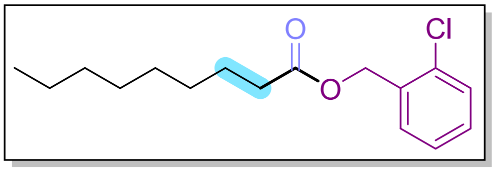

Yield 69 %, 38.8 mg, *n/iso* = 90 : 10, light yellow oil.

^{1}H NMR (400 MHz, CDCl$_3$) δ 7.44 – 7.36 (m, 2H), 7.29 – 7.24 (m, 2H), 5.22 (s, 2H), 2.38 (t, J = 7.5 Hz, 2H), 1.72 – 1.60 (m, 2H), 1.37 – 1.20 (m, 10H), 0.88 (t, J = 6.9 Hz, 3H).

^{13}C NMR (101 MHz, CDCl$_3$) δ 173.6, 133.9, 133.8, 129.9, 129.7, 129.6, 127.0, 63.6, 34.4, 31.9, 29.3, 29.3, 25.1, 22.8, 14.2.

HRMS(EI): m/z calcd for $C_{16}H_{23}O_2Cl_1^+$: 282.13811, Found: 282.13816 [M]$^+$.

Phenethyl nonanoate, 31

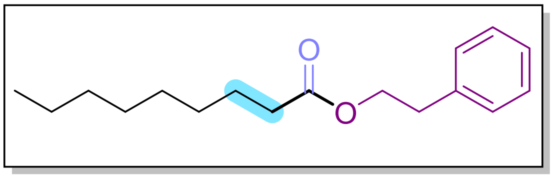

Yield 50 %, 26.2 mg, *n/iso* = 90 : 10, light yellow oil.

^{1}H NMR (400 MHz, CDCl$_3$) δ 7.34 – 7.27 (m, 2H), 7.26 – 7.19 (m, 3H), 4.29 (t, J = 7.1 Hz, 2H), 2.94 (t, J = 7.1 Hz, 2H), 2.28 (t, J = 7.5 Hz, 2H), 1.65 – 1.52 (m, 2H), 1.36 – 1.16 (m, 10H), 0.88 (t, J = 6.9 Hz, 3H).

^{13}C NMR (101 MHz, CDCl$_3$) δ 174.0, 138.0, 129.0, 128.6, 126.7, 64.8, 35.3, 34.5, 32.0, 29.4, 29.3, 25.1, 22.8, 14.2.

HRMS(ESI): m/z calcd for $C_{17}H_{26}O_2Na^+$: 285.1830, Found: 285.1839.

Naphthalen-2-ylmethyl nonanoate, 32

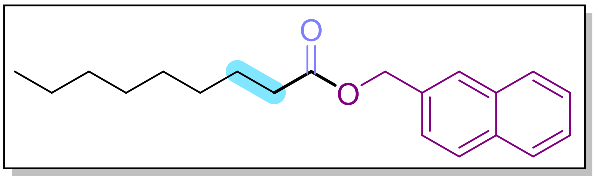

Yield 47 %, 27.8 mg, *n/iso* = 90 : 10, light yellow oil.

^{1}H NMR (400 MHz, CDCl$_3$) δ 7.87 – 7.81 (m, 4H), 7.53 – 7.44 (m, 3H), 5.29 (s, 2H), 2.39 (t, J = 7.5 Hz, 2H), 1.74 – 1.62 (m, 2H), 1.39 – 1.22 (m, 10H), 0.88 (t, J = 7.1 Hz, 3H).

^{13}C NMR (101 MHz, CDCl$_3$) δ 173.9, 133.7, 133.3, 133.2, 128.5, 128.1, 127.8, 127.4, 126.4, 126.4, 126.0, 66.3, 34.5, 31.9, 29.4, 29.3, 29.3, 25.1, 22.8, 14.2.

HRMS(EI): m/z calcd for $C_{20}H_{26}O_2^+$: 298.19273, Found: 298.19245 [M]$^+$.

Butyl 3,5,5-trimethylhexanoate, 33

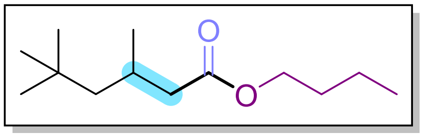

Yield 65 %, 28.1 mg, *n/iso* > 99 : 1, light yellow oil.

^{1}H NMR (400 MHz, $CDCl_3$) δ 4.10 – 3.99 (m, 2H), 2.29 (dd, *J* = 14.3, 6.0 Hz, 1H), 2.12 (dd, *J* = 14.4, 8.1 Hz, 1H), 2.08 – 1.97 (m, 1H), 1.67 – 1.55 (m, 2H), 1.44 – 1.31 (m, 2H), 1.24 (dd, *J* = 14.0, 4.0 Hz, 1H), 1.11 (dd, *J* = 14.0, 6.3 Hz, 1H), 0.97 (d, *J* = 6.6 Hz, 3H), 0.93 (t, *J* = 7.4 Hz, 3H), 0.90 (s, 9H).

^{13}C NMR (101 MHz, $CDCl_3$) δ 173.5, 64.2, 50.7, 44.3, 31.2, 30.9, 30.1, 27.2, 22.8, 19.3, 13.8.

HRMS (ESI): m/z calcd for $C_{13}H_{26}O_2Na_1^+$: 237.182501, found 237.1824467.

Hexyl 3,5,5-trimethylhexanoate, 34

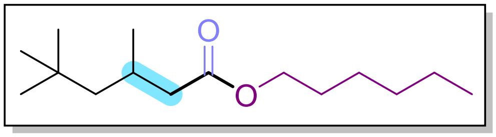

Yield 52 %, 25.3 mg, *n/iso* > 99 : 1, light yellow oil

^{1}H NMR (400 MHz, $CDCl_3$) δ 4.10 – 3.99 (m, 2H), 2.29 (dd, *J* = 14.3, 5.9 Hz, 1H), 2.12 (dd, *J* = 14.3, 8.0 Hz, 1H), 2.08 – 1.98 (m, 1H), 1.67 – 1.55 (m, 2H), 1.40 – 1.27 (m, 6H), 1.24 (dd, *J* = 14.1, 3.9 Hz, 1H), 1.11 (dd, *J* = 14.0, 6.4 Hz, 1H), 0.97 (d, *J* = 6.5 Hz, 3H), 0.94 – 0.85 (m, 12H).

^{13}C NMR (101 MHz, $CDCl_3$) δ 173.5, 64.5, 50.7, 44.3, 31.6, 31.2, 30.1, 28.8, 27.2, 25.8, 22.8, 22.7, 14.1.

HRMS (ESI): m/z calcd for $C_{15}H_{30}O_2Na_1^+$: 265.213801, found 265.2137468.

Octyl 3,5,5-trimethylhexanoate, 35

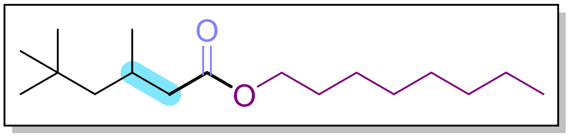

Yield 61 %, 32.9 mg, *n/iso* > 99 : 1, light yellow oil

^{1}H NMR (400 MHz, CDCl$_3$) δ 4.10-3.99 (m, 2H), 2.29 (dd, *J* = 14.3, 6.0 Hz, 1H), 2.11 (dd, *J* = 14.3, 8.0 Hz, 1H), 2.08 – 1.98 (m, 1H), 1.67 – 1.55 (m, 2H), 1.38 – 1.20 (m, 11H), 1.11 (dd, *J* = 14.1, 6.3 Hz, 1H), 0.97 (d, *J* = 6.5 Hz, 3H), 0.93 – 0.82 (m, 12H).

^{13}C NMR (101 MHz, CDCl$_3$) δ 173.4, 64.5, 50.7, 44.3, 31.9, 31.2, 30.1, 29.3, 29.3, 28.8, 27.2, 26.1, 22.8, 22.8, 14.2.

HRMS (ESI): m/z calcd for $C_{17}H_{34}O_2Na_1^+$: 293.245101, found 293.2449836.

Naphthalen-2-ylmethyl 3,5,5-trimethylhexanoate, 36

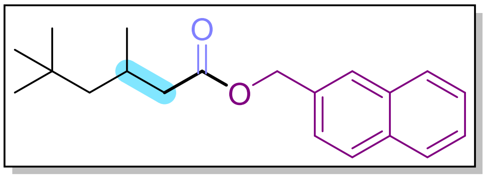

Yield 52 %, 31.0 mg, *n/iso* > 99 : 1, light yellow oil.

^{1}H NMR (300 MHz, CDCl$_3$) δ 7.89 – 7.80 (m, 4H), 7.54 – 7.43 (m, 3H), 5.29 (d, *J* = 1.5 Hz, 2H), 2.40 (dd, *J* = 14.5, 6.1 Hz, 1H), 2.23 (dd, *J* = 14.5, 8.0 Hz, 1H), 2.16 – 2.01 (m, 1H), 1.27 (dd, *J* = 14.1, 4.0 Hz, 1H), 1.13 (dd, *J* = 14.0, 6.4 Hz, 1H), 1.00 (d, *J* = 6.6 Hz, 3H), 0.90 (s, 9H).

^{13}C NMR (75 MHz, CDCl$_3$) δ 173.1, 133.6, 133.2, 133.1, 128.4, 128.0, 127.7, 127.4, 126.3, 126.2, 126.0, 66.2, 50.5, 44.0, 31.1, 30.0, 27.1, 22.7.

HRMS(EI): m/z calcd for $C_{20}H_{26}O_2^+$: 298.19273, Found: 298.19323 $[M]^+$.

4-(4,4,5,5-tetramethyl-1,3,2-dioxaborolan-2-yl)benzyl 3,5,5-trimethylhexanoate, 37,

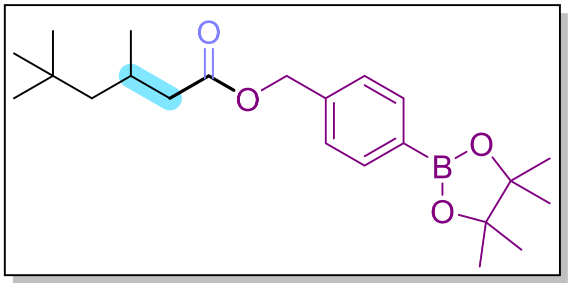

Yield 45 %,33.8 mg, *n/iso* > 99 : 1, light yellow oil.

^{1}H NMR (300 MHz, CDCl$_3$) δ 7.77 – 7.62 (m, 2H), 7.36 – 7.23 (m, 2H), 5.05 (d, *J* = 1.7 Hz, 2H), 2.29 (dd, *J* = 14.5, 6.0 Hz, 1H), 2.11 (dd, *J* = 14.5, 8.0 Hz, 1H), 2.05 – 1.92 (m, 1H), 1.27 (s, 12H), 1.17 (dd, *J* = 14.1, 4.1 Hz, 1H), 1.04 (dd, *J* = 14.1, 6.3 Hz, 1H), 0.90 (d, *J* = 6.5 Hz, 3H), 0.82 (s, 9H).

^{13}C NMR (75 MHz, $CDCl_3$) δ 173.1, 139.3, 135.1, 127.4, 84.0, 66.0, 50.6, 44.1, 31.2, 30.1, 27.2, 25.0, 22.8.

HRMS(EI): m/z calcd for $C_{22}H_{35}O_4B_1^+$: 374.26229, Found: 374.26278 $[M]^+$.

2-Phenoxyethyl 3,5,5-trimethylhexanoate, 38

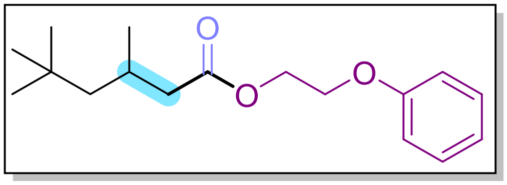

Yield 37 %, 20.8 mg, *n/iso* > 99 : 1, light yellow oil

^1H NMR (300 MHz, $CDCl_3$) δ 7.31 – 7.22 (m, 2H), 6.98 – 6.85 (m, 3H), 4.44 – 4.39 (m, 2H), 4.18 – 4.13 (m, 2H), 2.33 (dd, *J* = 14.6, 6.1 Hz, 1H), 2.17 (dd, *J* = 14.6, 7.9 Hz, 1H), 2.10 – 1.96 (m, 1H), 1.24 (dd, *J* = 14.0, 4.0 Hz, 1H), 1.10 (dd, *J* = 14.1, 6.4 Hz, 1H), 0.97 (d, *J* = 6.6 Hz, 3H), 0.88 (s, 9H).

^{13}C NMR (75 MHz, $CDCl_3$) δ 173.3, 158.6, 129.7, 121.3, 114.8, 66.0, 62.7, 50.6, 44.0, 31.2, 30.1, 27.2, 22.8.

HRMS(EI): m/z calcd for $C_{17}H_{26}O_3^+$: 278.18765, Found: 278.18772 $[M]^+$.

2-Chloroethyl 3,5,5-trimethylhexanoate, 39

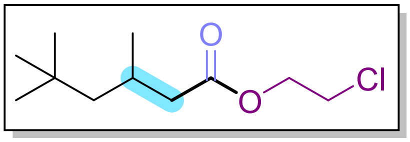

Yield 50 %, 21.9 mg, *n/iso* > 99 : 1, light yellow oil.

^1H NMR (300 MHz, $CDCl_3$) δ 4.39 – 4.25 (m, 2H), 3.68 (t, *J* = 5.7 Hz, 2H), 2.35 (dd, *J* = 14.6, 6.0 Hz, 1H), 2.18 (dd, *J* = 14.5, 8.0 Hz, 1H), 2.12 – 1.98 (m, 1H), 1.26 (dd, *J* = 14.1, 4.0 Hz, 1H), 1.13 (dd, *J* = 14.0, 6.4 Hz, 1H), 0.99 (d, *J* = 6.6 Hz, 3H), 0.91 (s, 9H).

^{13}C NMR (75 MHz, $CDCl_3$) δ 172.9, 63.9, 50.6, 43.9, 41.8, 31.2, 30.1, 27.2, 22.8.

HRMS (ESI): m/z calculated for $C_{11}H_{21}Cl_1O_2Na_1^+$: 243.112229, found 243.1119977.

Phenethyl 3,5,5-trimethylhexanoate, 40

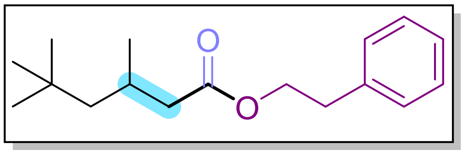

Yield 67 %, 35.0 mg, *n/iso* > 99 : 1, light yellow oil.

^{1}H NMR (300 MHz, CDCl$_3$) δ 7.34 – 7.18 (m, 5H), 4.37 – 4.21 (m, 2H), 2.95 (t, *J* = 7.1 Hz, 2H), 2.30 (dd, *J* = 14.3, 5.7 Hz, 1H), 2.11 (dd, *J* = 14.3, 8.2 Hz, 1H), 2.06 – 1.93 (m, 1H), 1.22 (dd, *J* = 14.0, 4.0 Hz, 1H), 1.10 (dd, *J* = 14.1, 6.1 Hz, 1H), 0.94 (d, *J* = 6.5 Hz, 3H), 0.90 (s, 9H).

^{13}C NMR (75 MHz, CDCl$_3$) δ 173.2, 138.0, 129.0, 128.6, 126.7, 64.8, 50.6, 44.2, 35.3, 31.2, 30.1, 27.1, 22.8.

HRMS (ESI): m/z calcd for $C_{17}H_{26}O_2Na^+$: 285.1830, Found: 285.1836.

2-(Trimethylsilyl) ethyl 3,5,5-trimethylhexanoate, 41

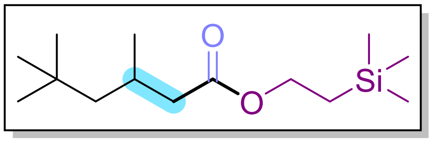

Yield 41 %, 21.3 mg, *n/iso* > 99 : 1, light yellow oil.

^{1}H NMR (300 MHz, CDCl$_3$) δ 4.20 – 4.10 (m, 2H), 2.28 (dd, *J* = 13.8, 5.3 Hz, 1H), 2.15 – 1.96 (m, 2H), 1.24 (dd, *J* = 14.0, 3.9 Hz, 1H), 1.11 (dd, *J* = 14.0, 6.1 Hz, 1H), 1.04 – 0.95 (m, 5H), 0.90 (s, 9H), 0.04 (s, 9H).

^{13}C NMR (75 MHz, CDCl$_3$) δ 173.5, 62.4, 50.7, 44.4, 31.2, 30.1, 27.2, 22.8, 17.5, -1.3.

HRMS (ESI): m/z calculated for $C_{14}H_{30}O_2Si_1Na_1^+$: 281.190728, found 281.1903616

^{1}H and ^{13}C NMR Spectra

Note: *The green highlights in the spectra denote peaks arising from the branched isomeric products.*

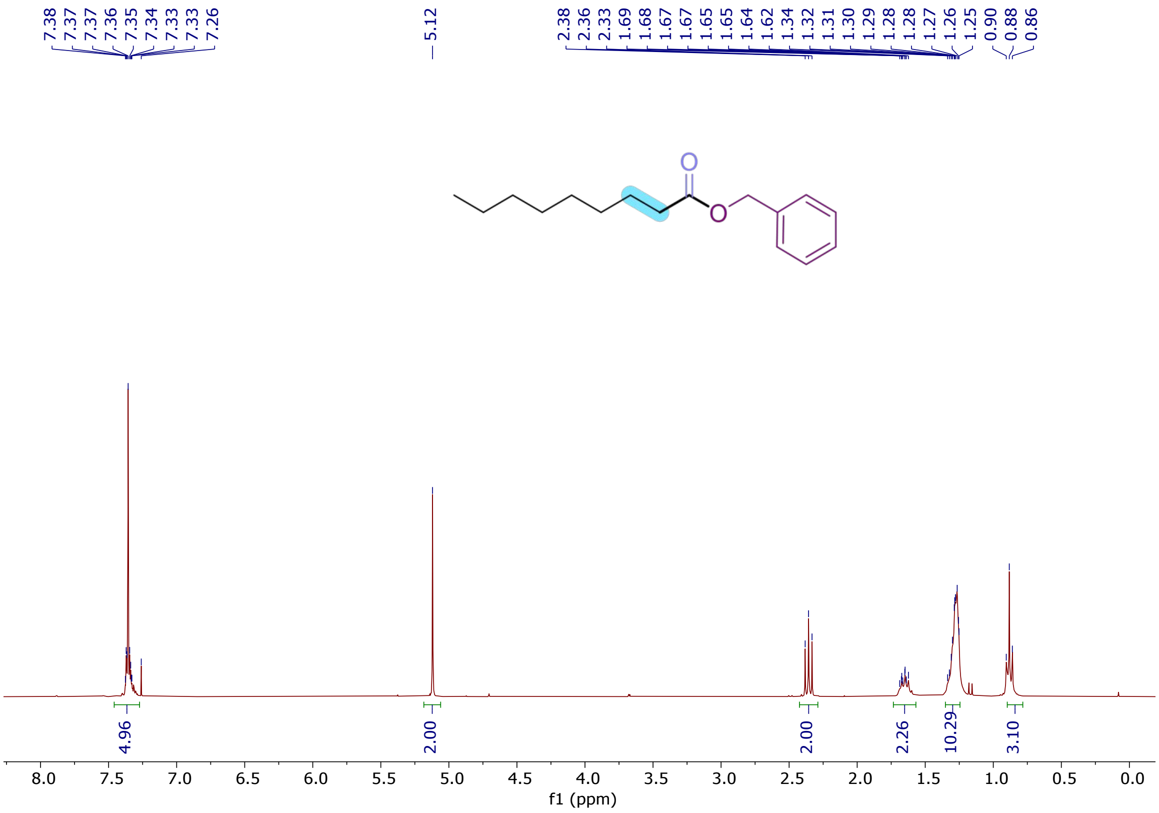

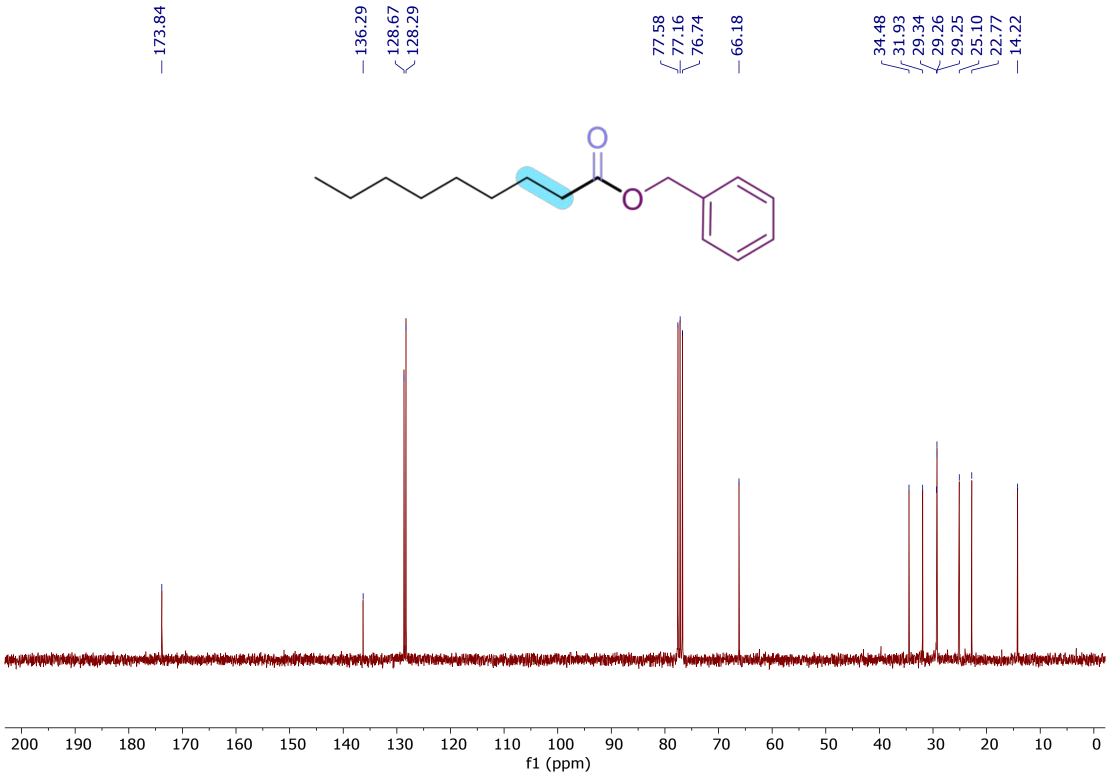

^{1}H NMR and ^{13}C NMR of **3**, *n/iso* = 91 : 9, (300 MHz, $CDCl_3$).

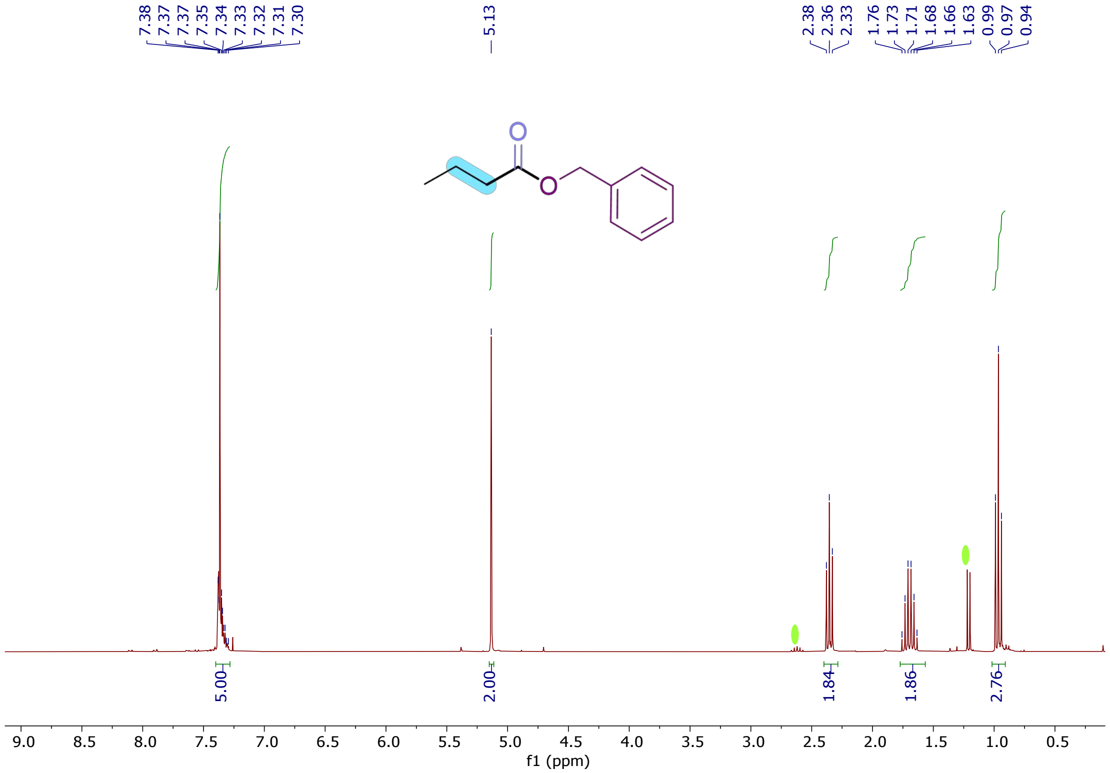

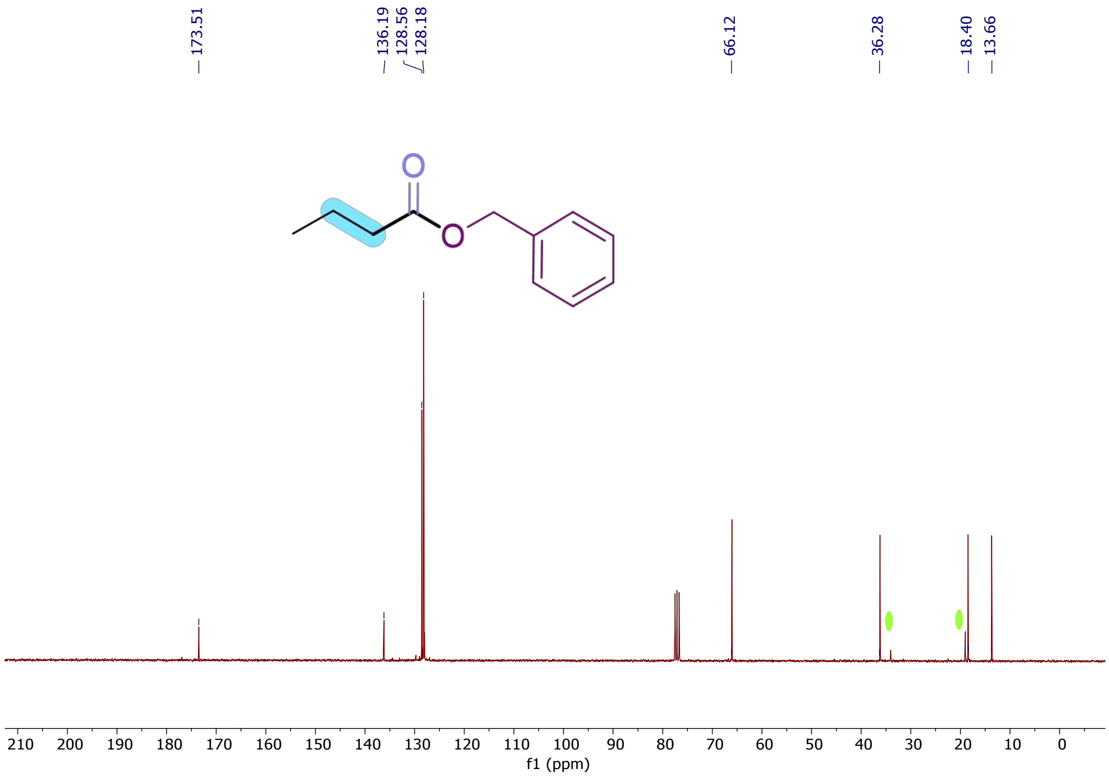

^{1}H NMR and ^{13}C NMR of **4**, *n/iso* = 90 : 10, (300 MHz, $CDCl_3$).

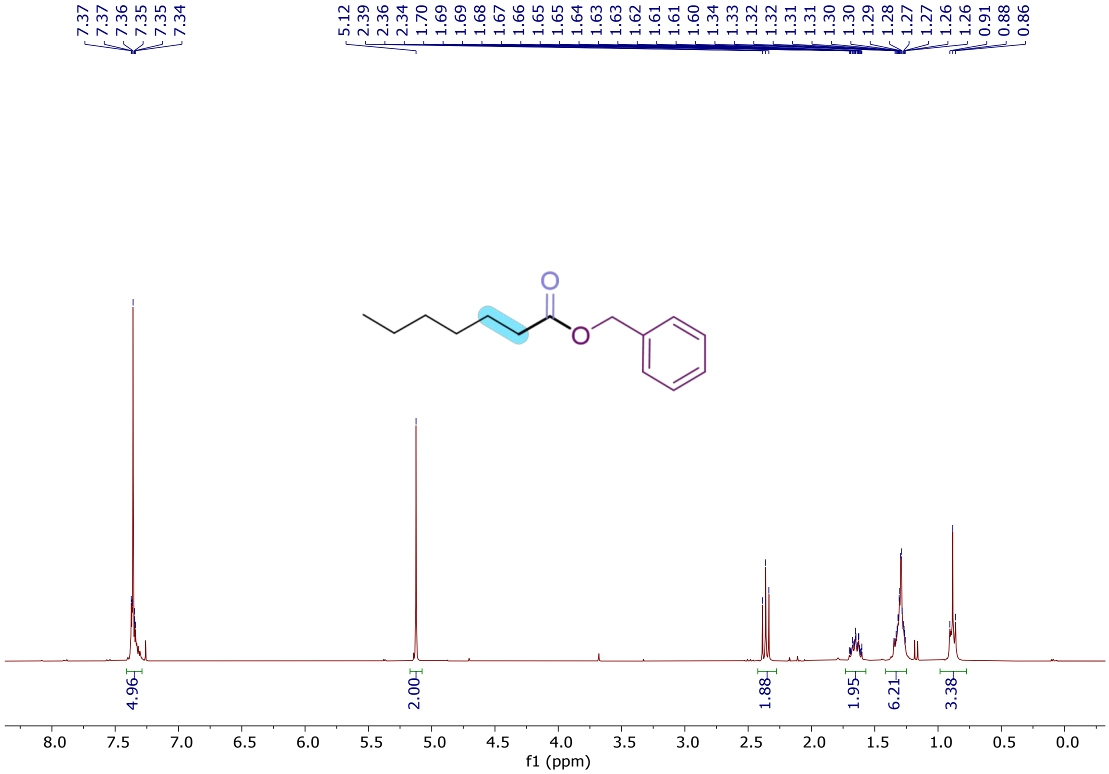

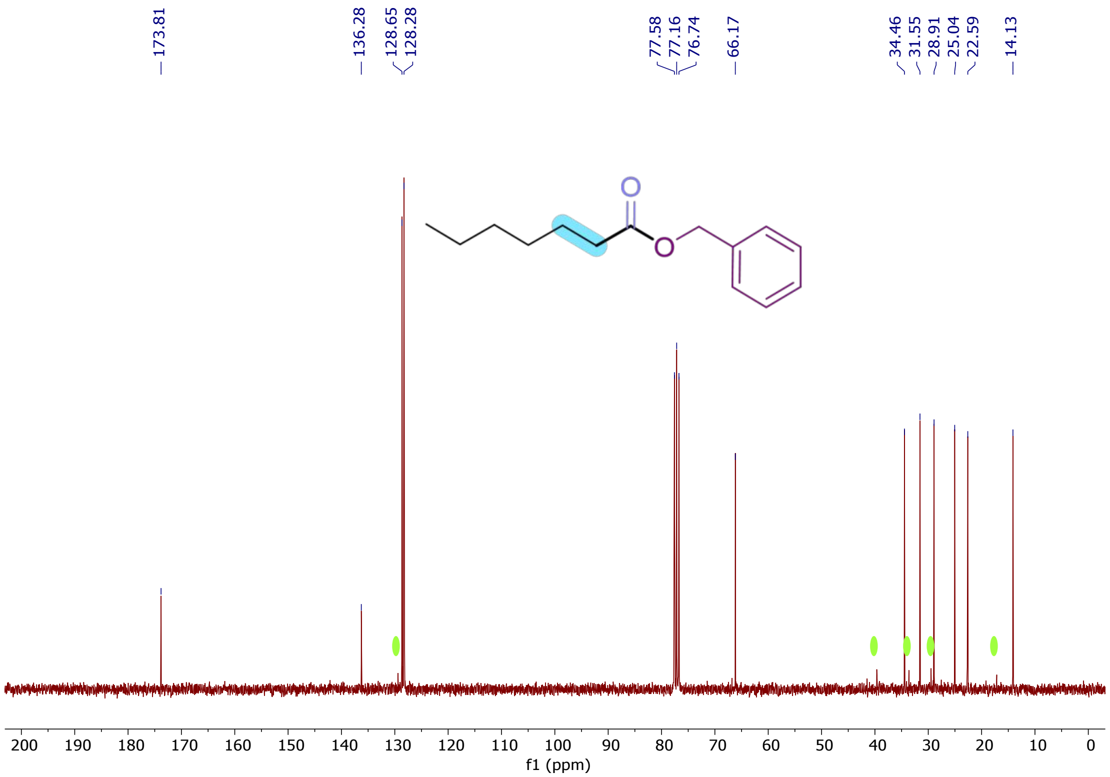

^{1}H NMR and ^{13}C NMR of **5**, *n/iso* = 91 : 9, (300 MHz, $CDCl_3$).

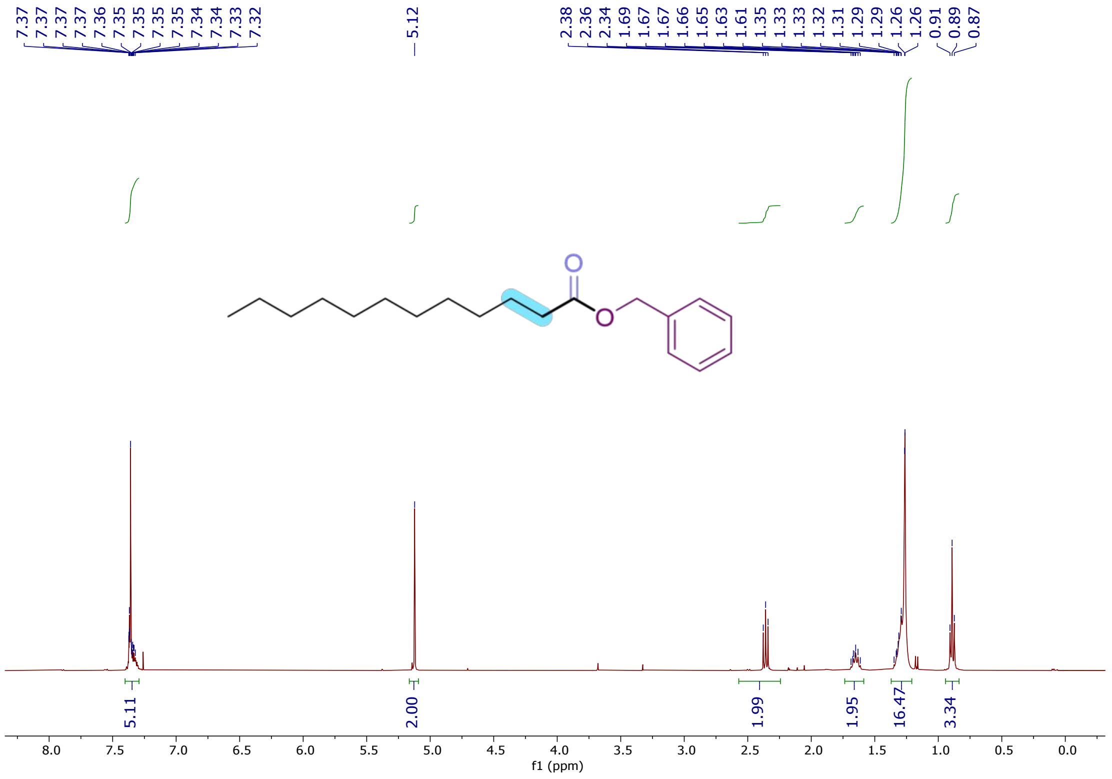

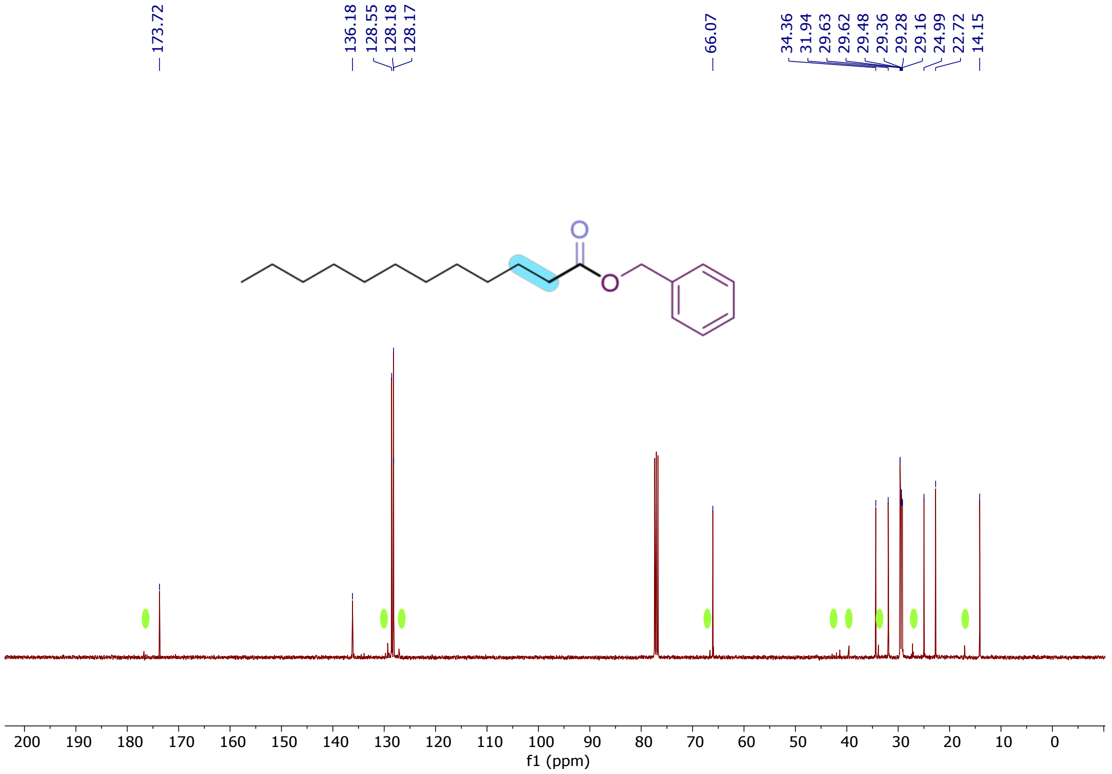

^{1}H NMR and ^{13}C NMR of **6**, *n/iso* = 92 : 8, (300 MHz, $CDCl_3$).

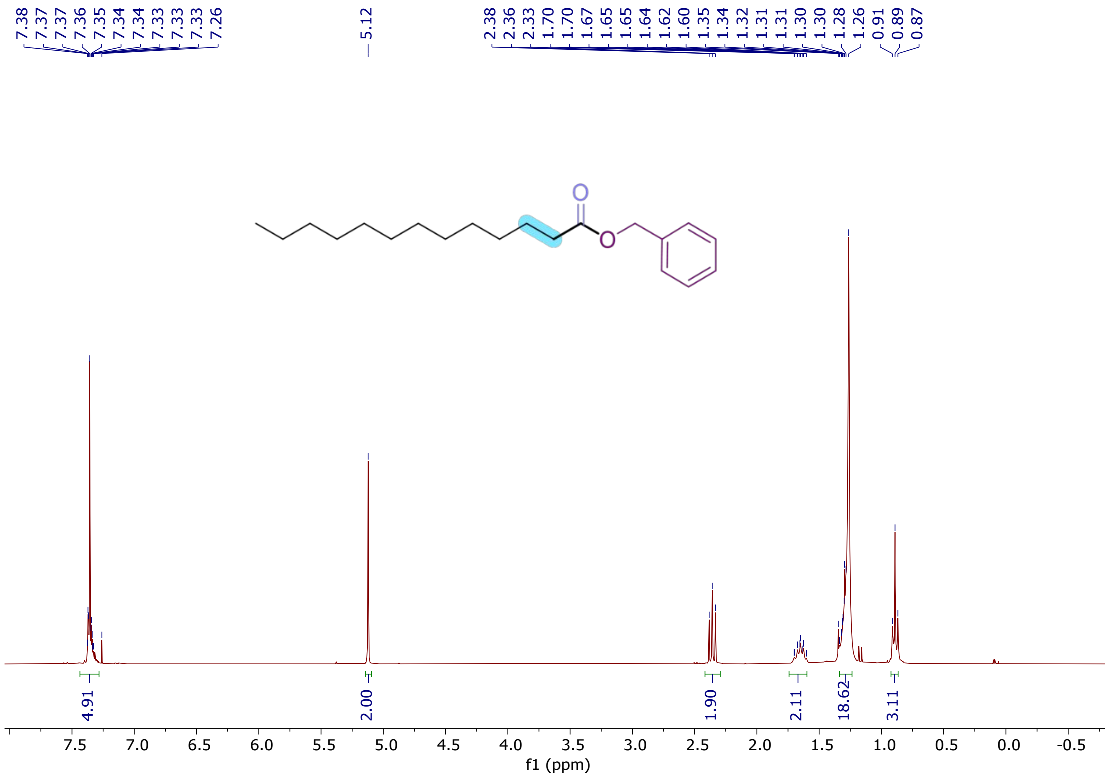

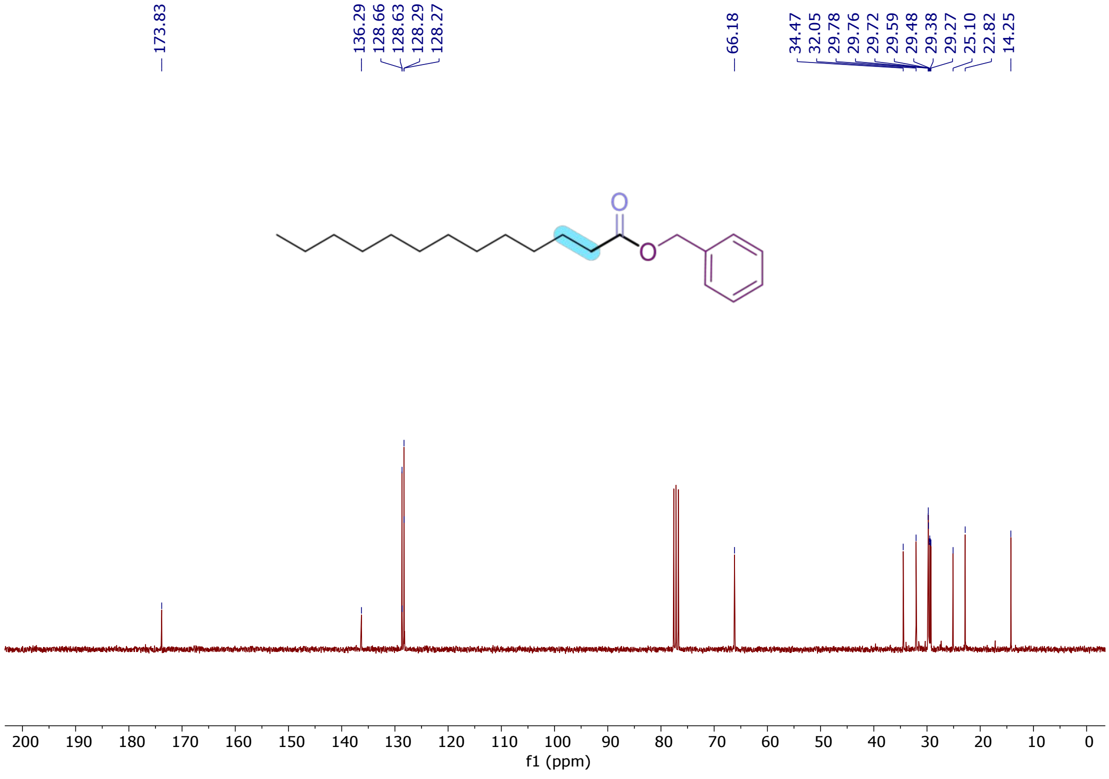

^{1}H NMR and ^{13}C NMR of **7**, *n/iso* = 92 : 8, (300 MHz, $CDCl_3$).

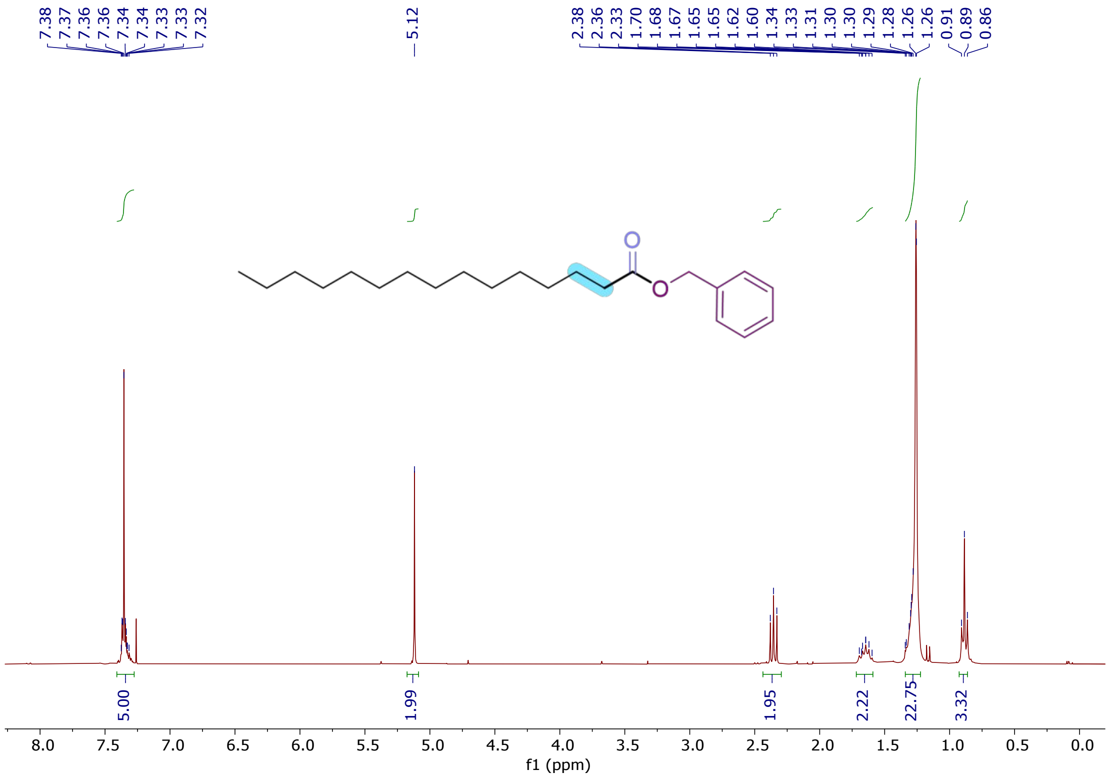

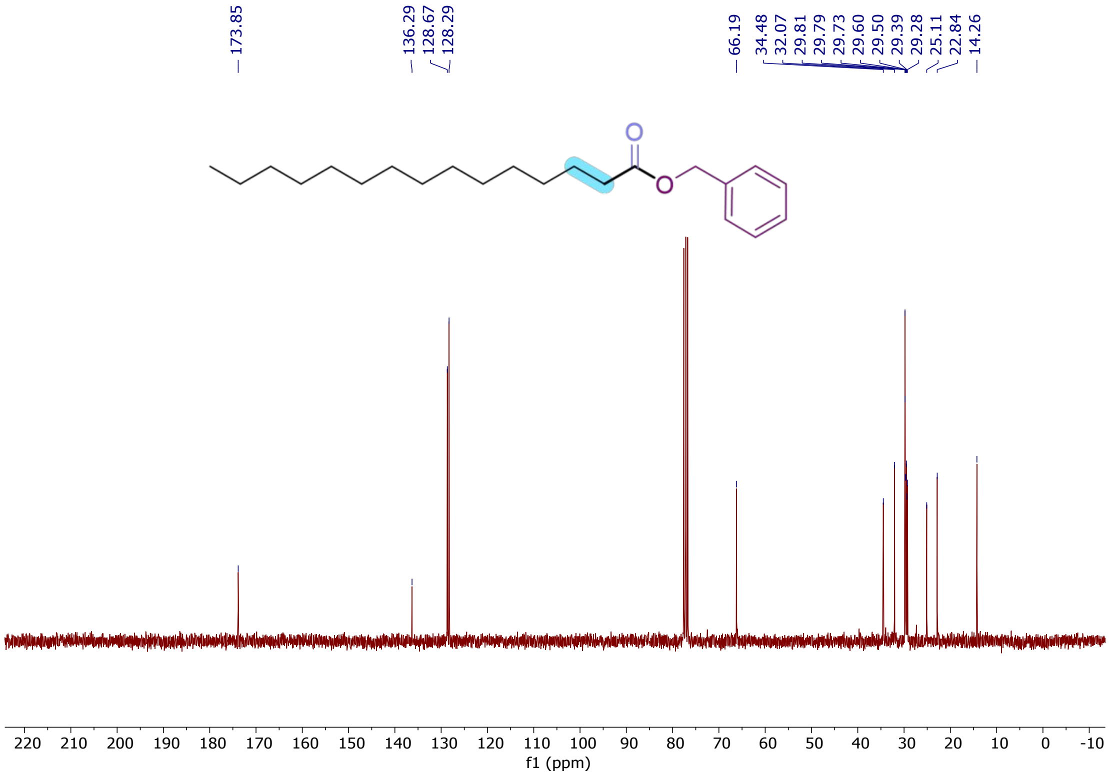

^{1}H NMR and ^{13}C NMR of **8**, *n/iso* = 92 : 8, (300 MHz, $CDCl_3$).

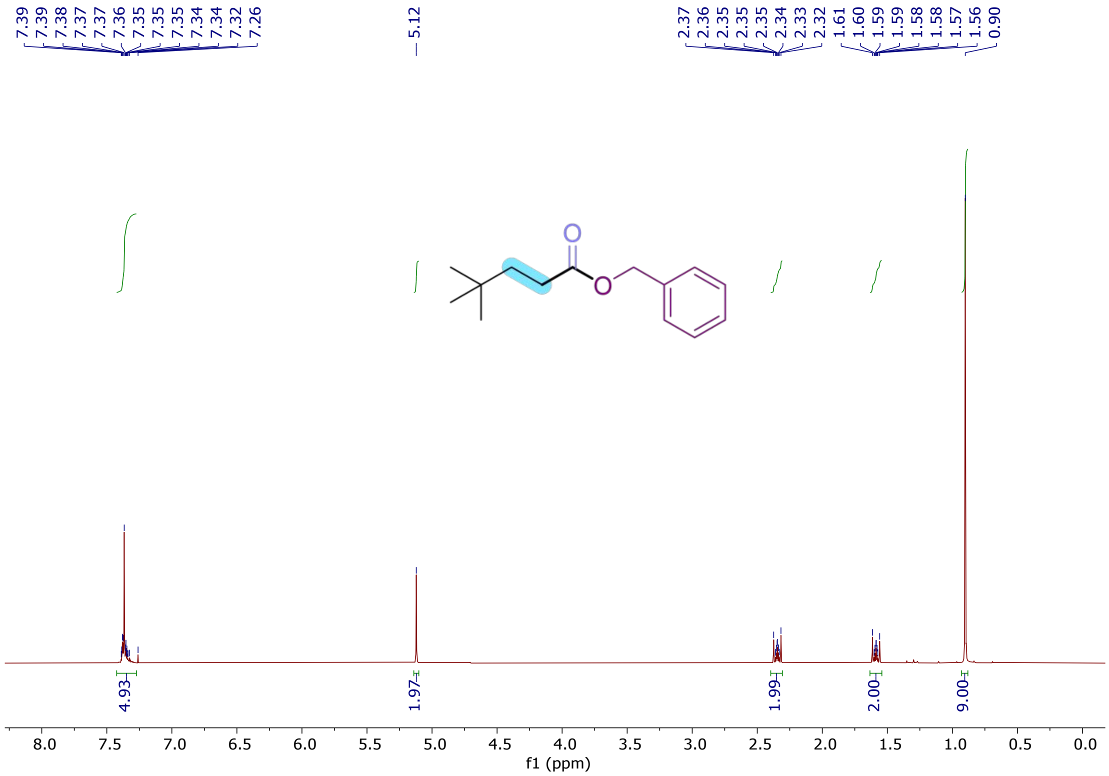

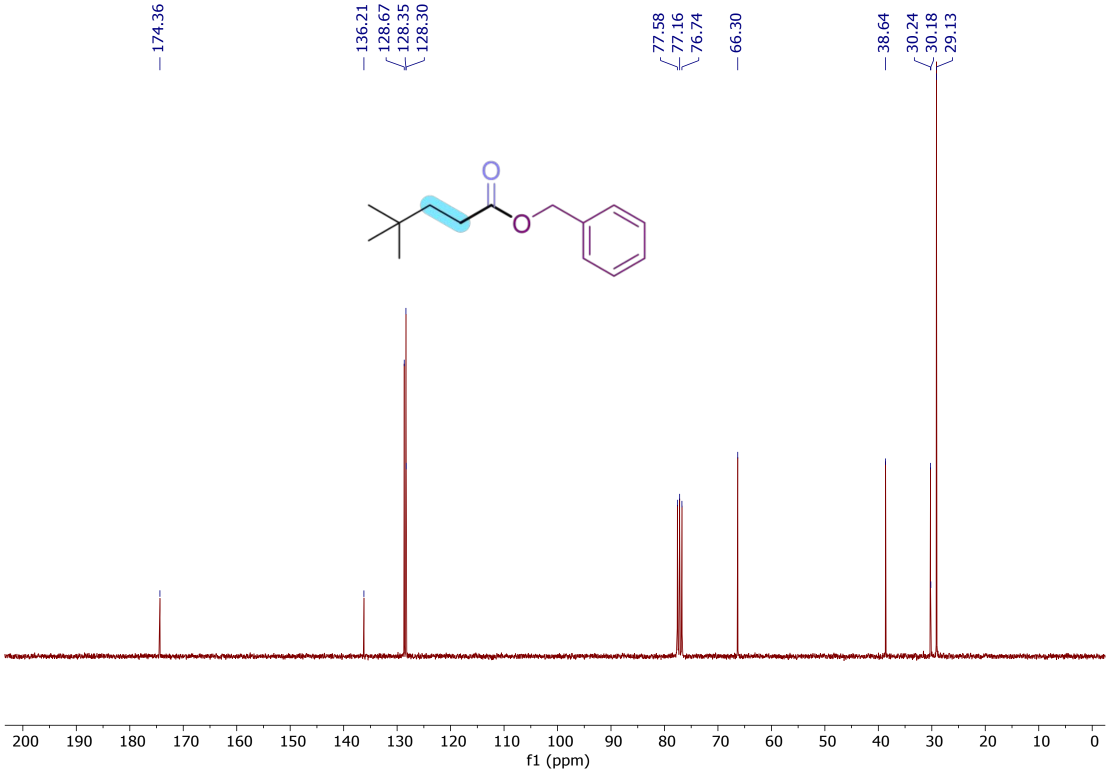

^{1}H NMR and ^{13}C NMR of **9** (300 MHz, $CDCl_3$).

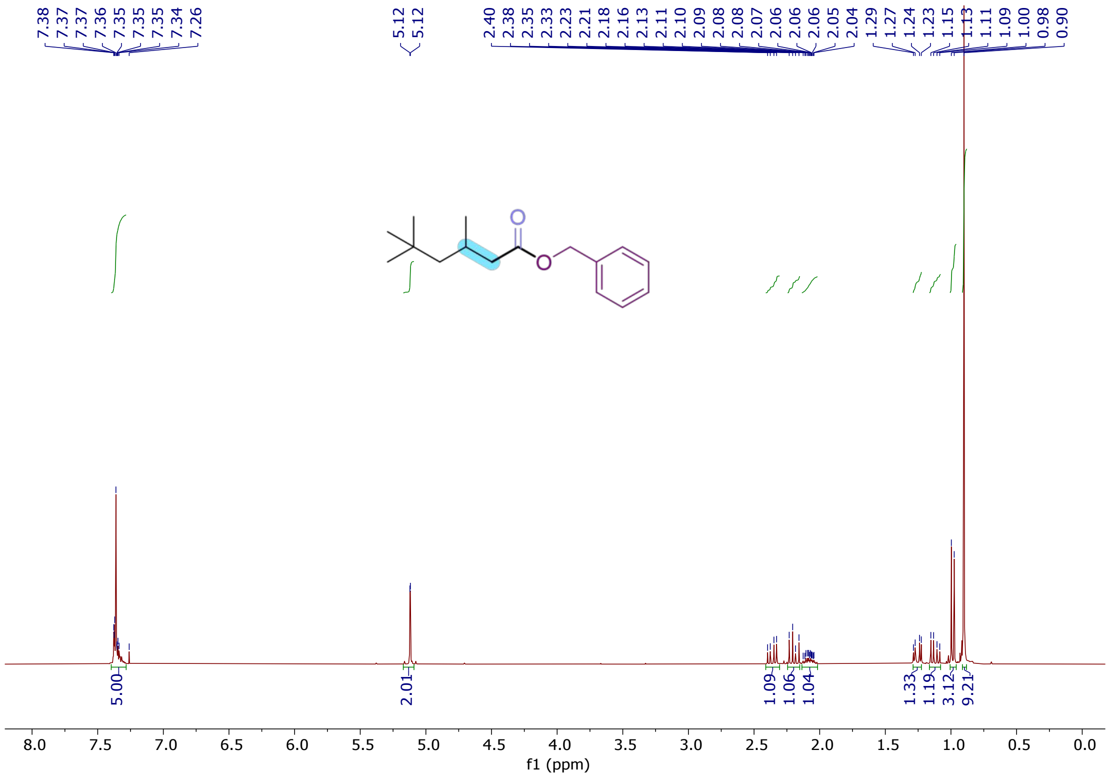

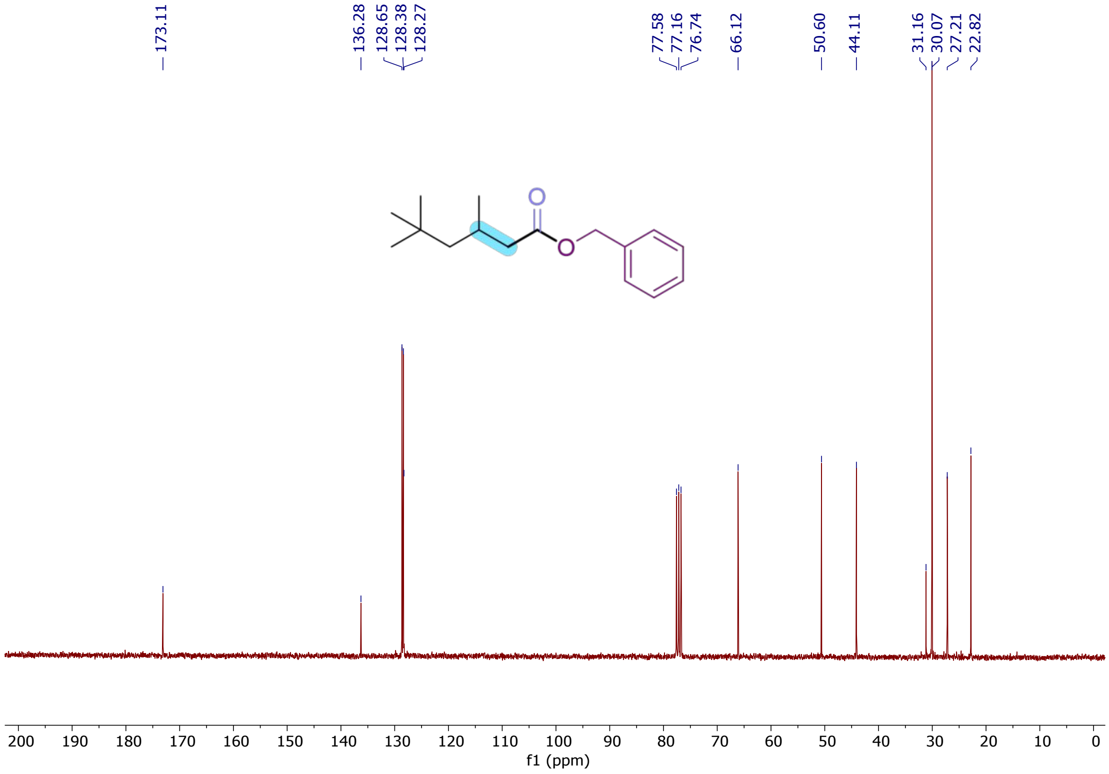

^{1}H NMR and ^{13}C NMR of **10** (300 MHz, $CDCl_3$).

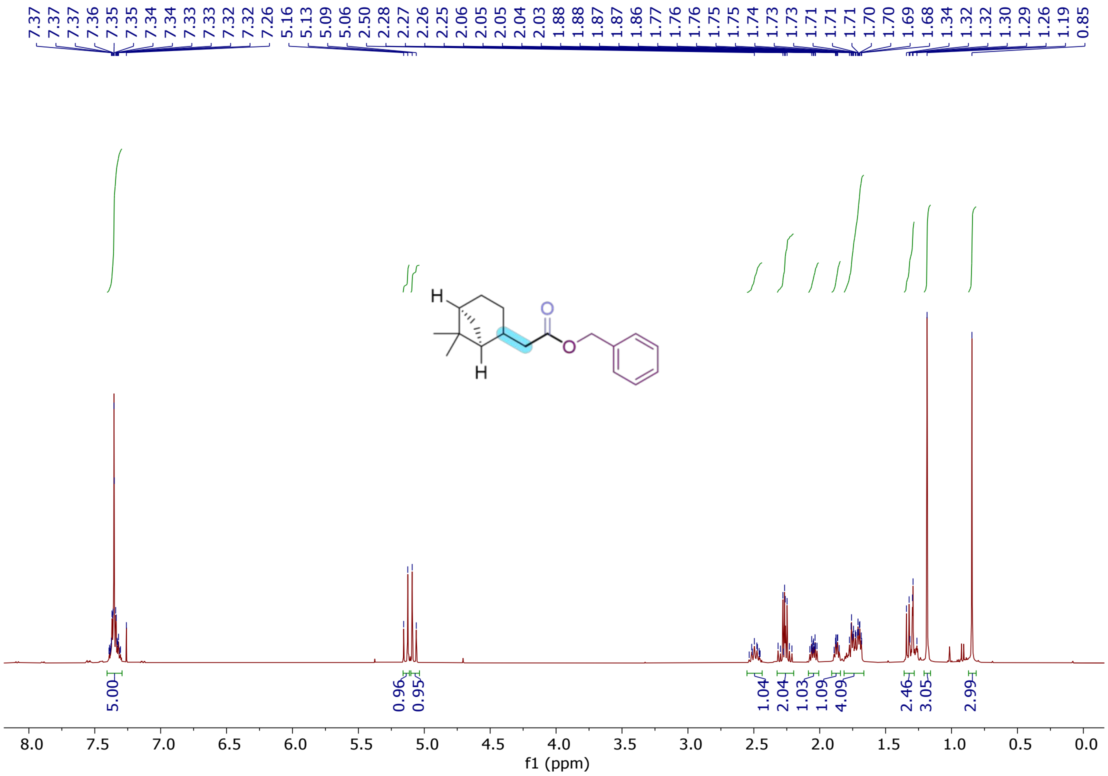

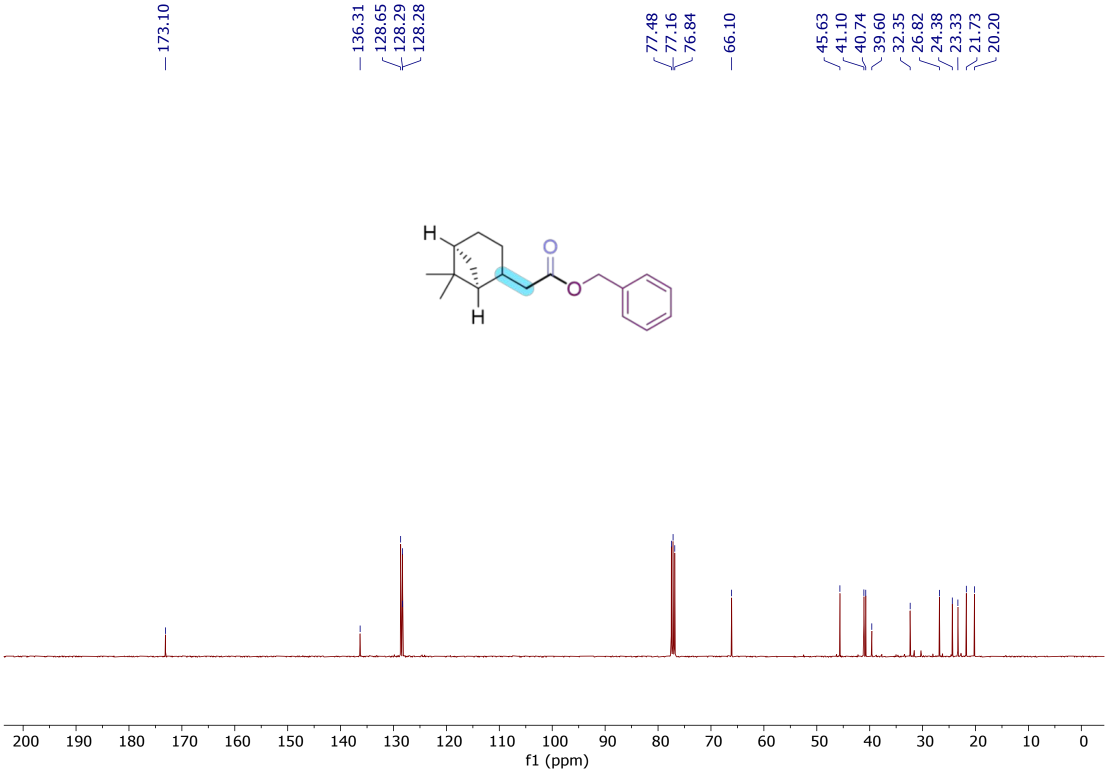

^1H NMR and ^{13}C NMR of **11** (400 MHz, $CDCl_3$).

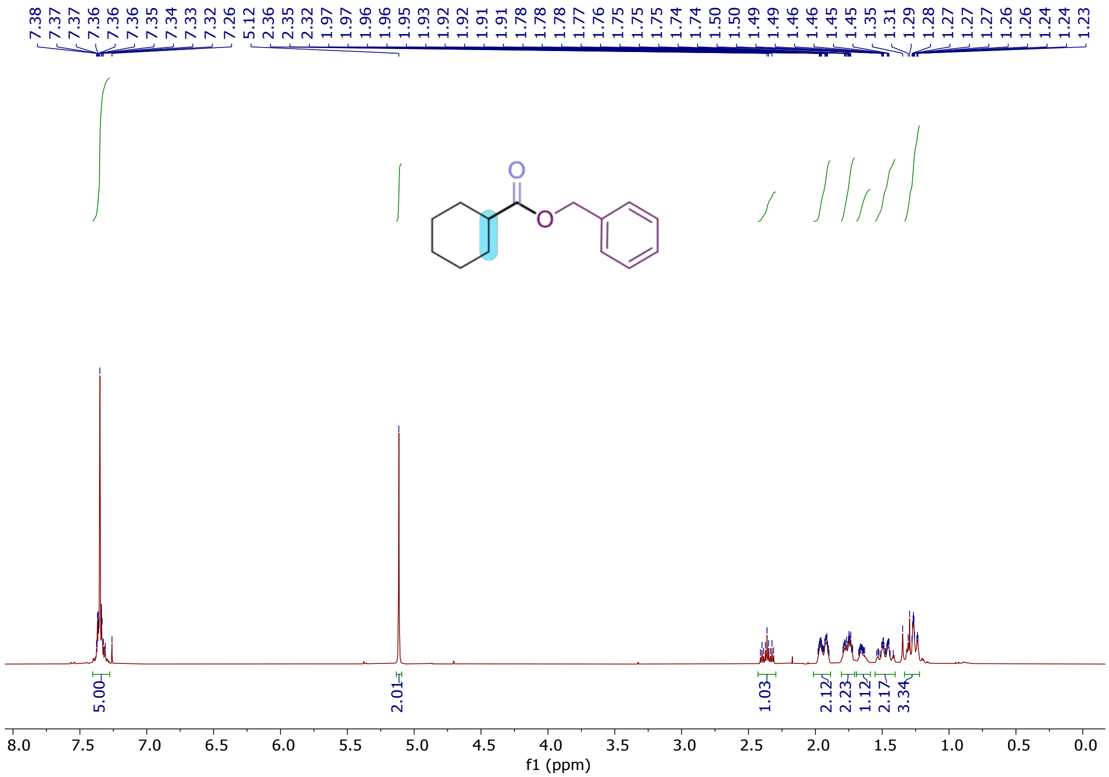

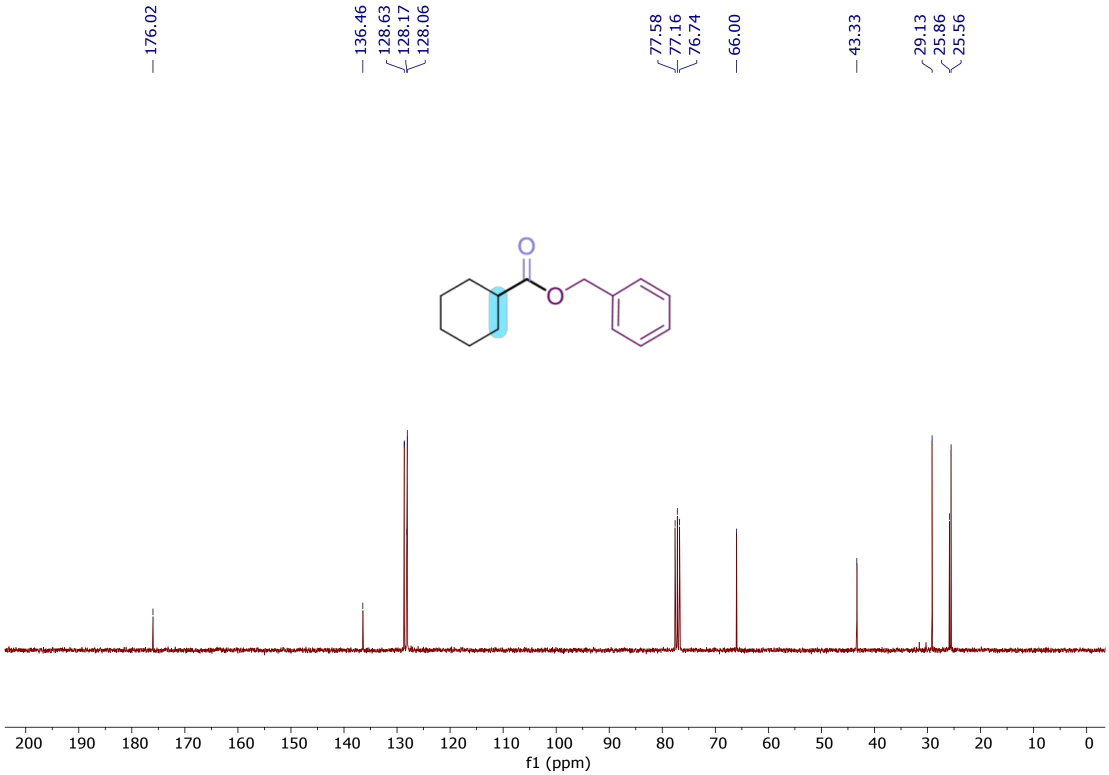

^{1}H NMR and ^{13}C NMR of **16** (300 MHz, $CDCl_3$).

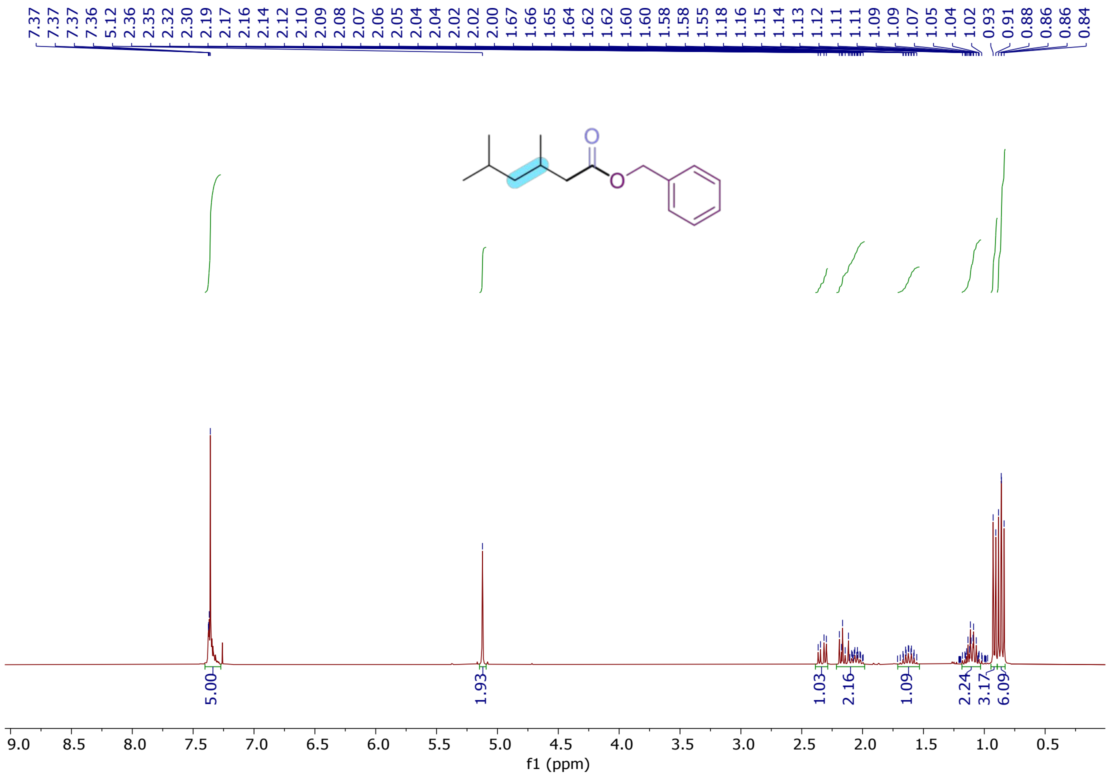

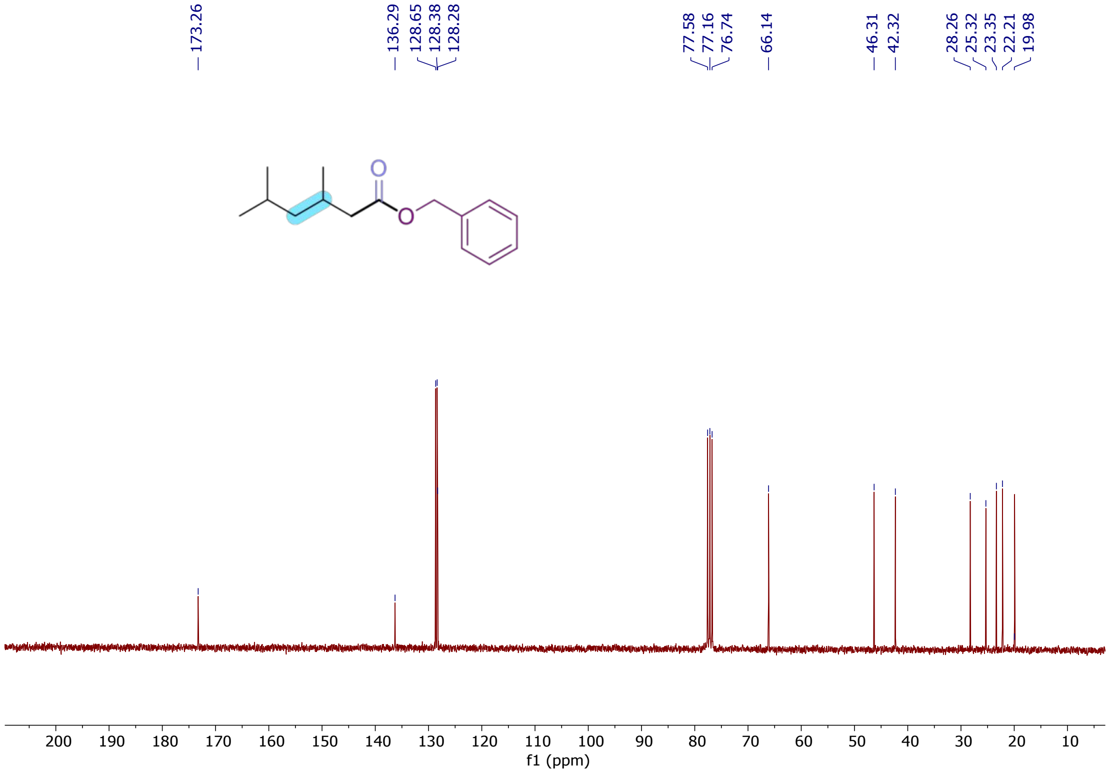

^{1}H NMR and ^{13}C NMR of **18** (300 MHz, $CDCl_3$).

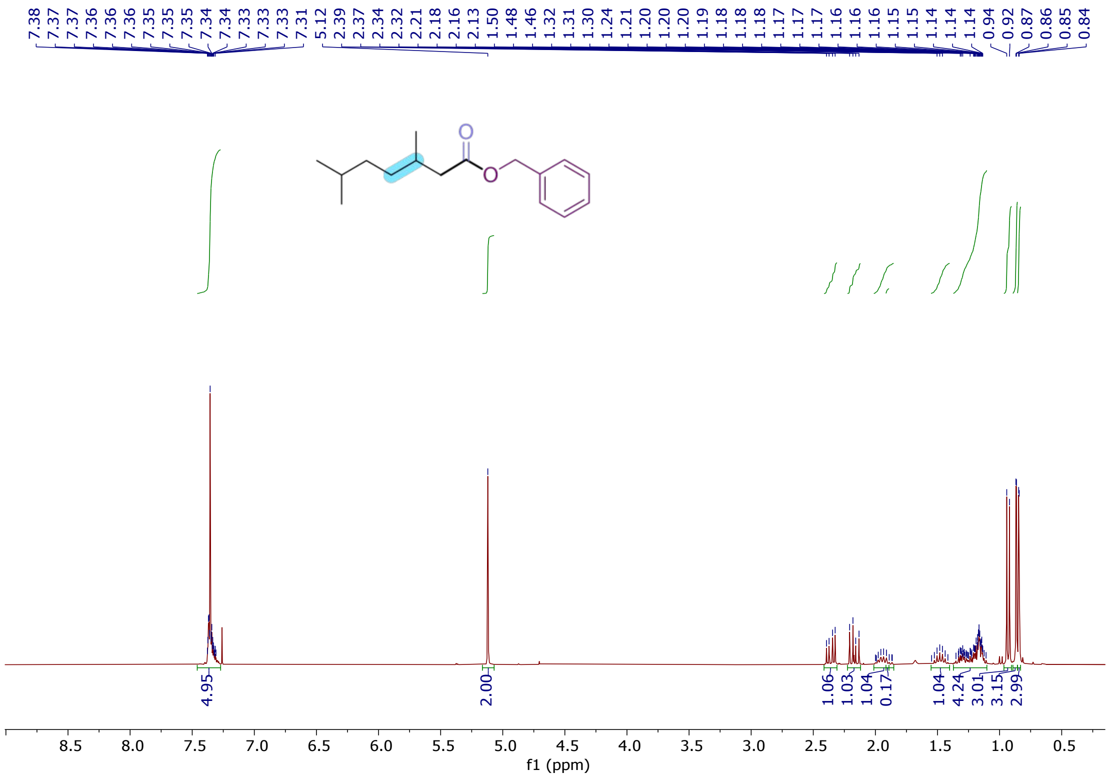

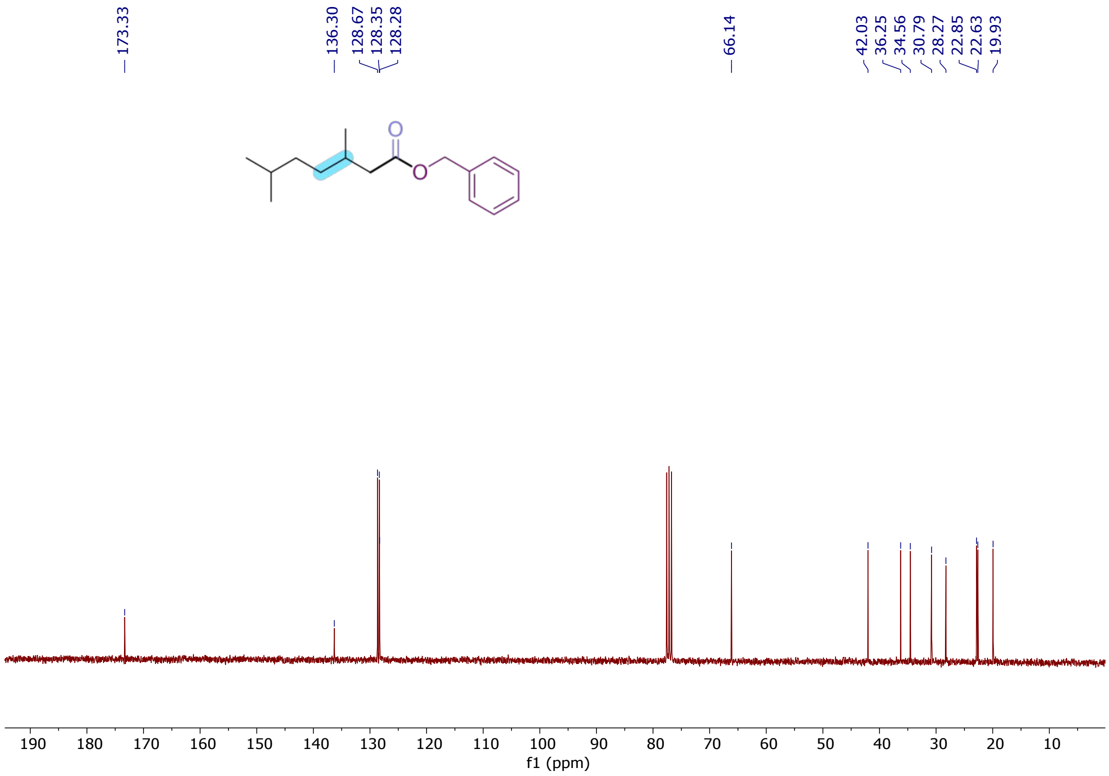

^{1}H NMR and ^{13}C NMR of **19**, (300 MHz, $CDCl_3$).

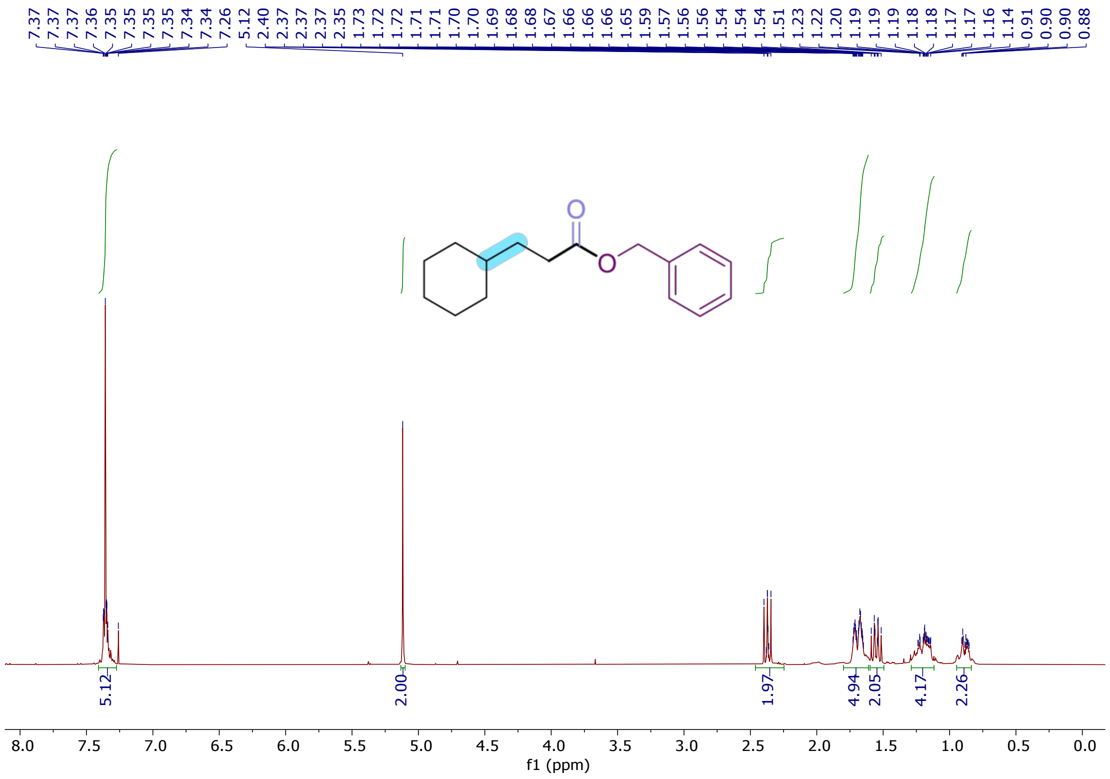

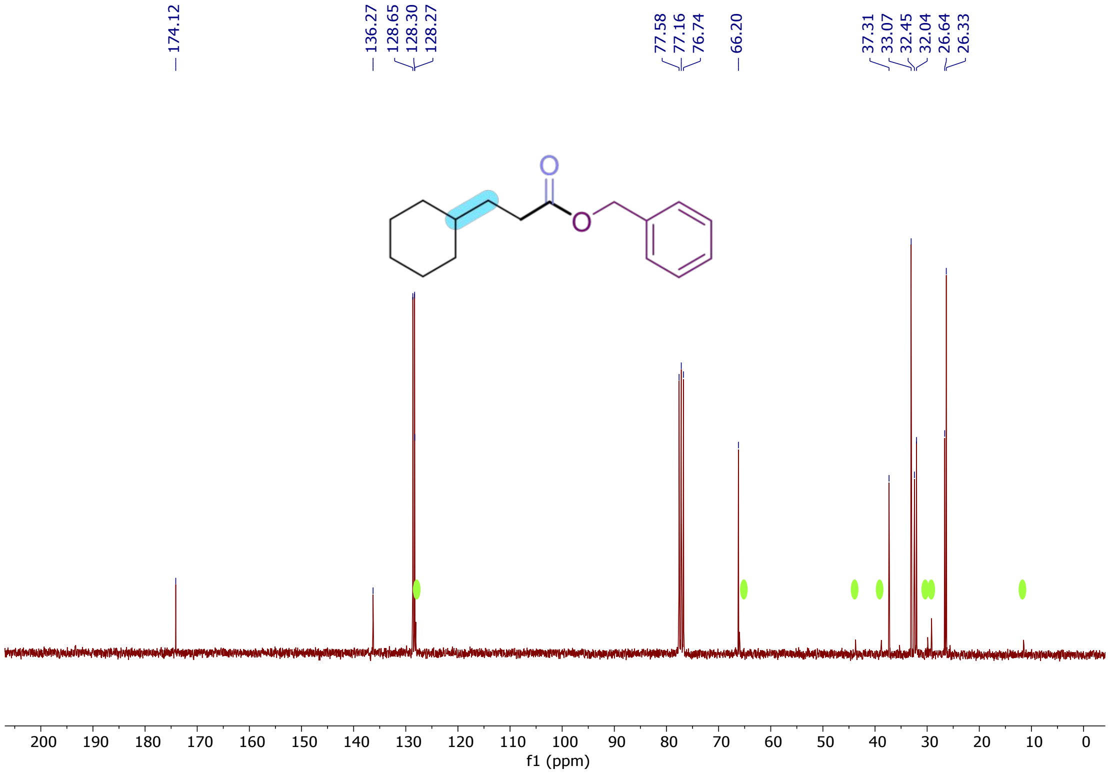

^{1}H NMR and ^{13}C NMR of **20**, *n/iso* = 90 :10 (300 MHz, $CDCl_3$).

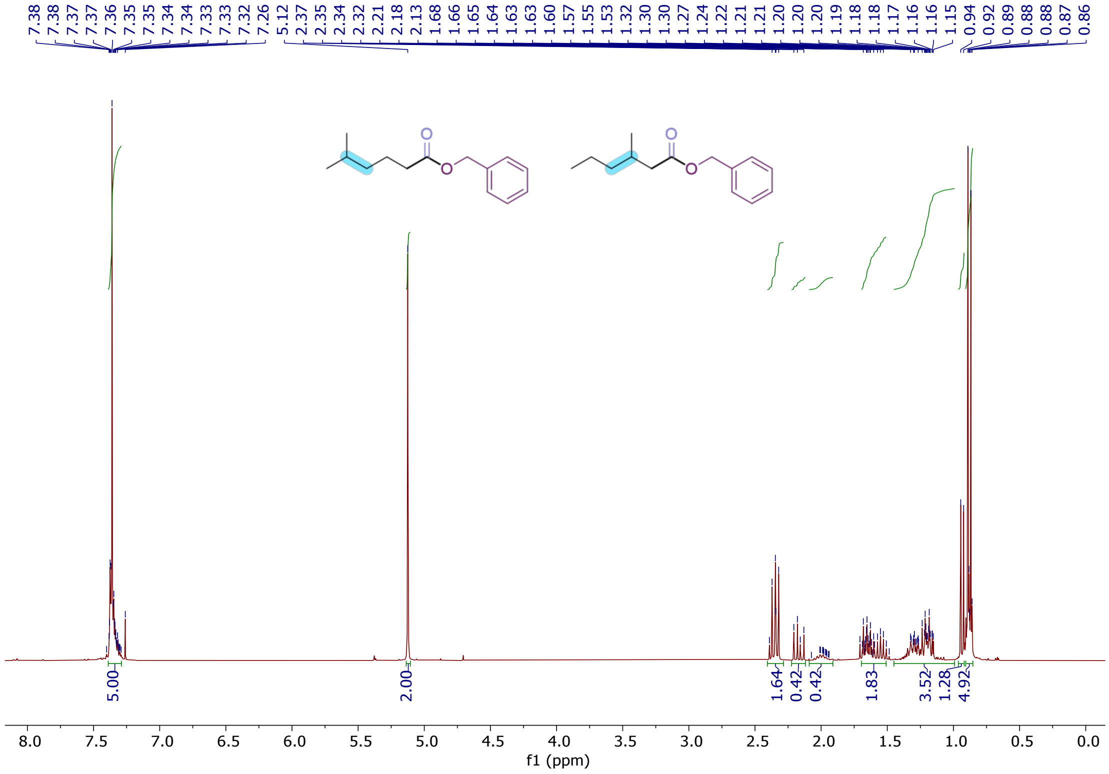

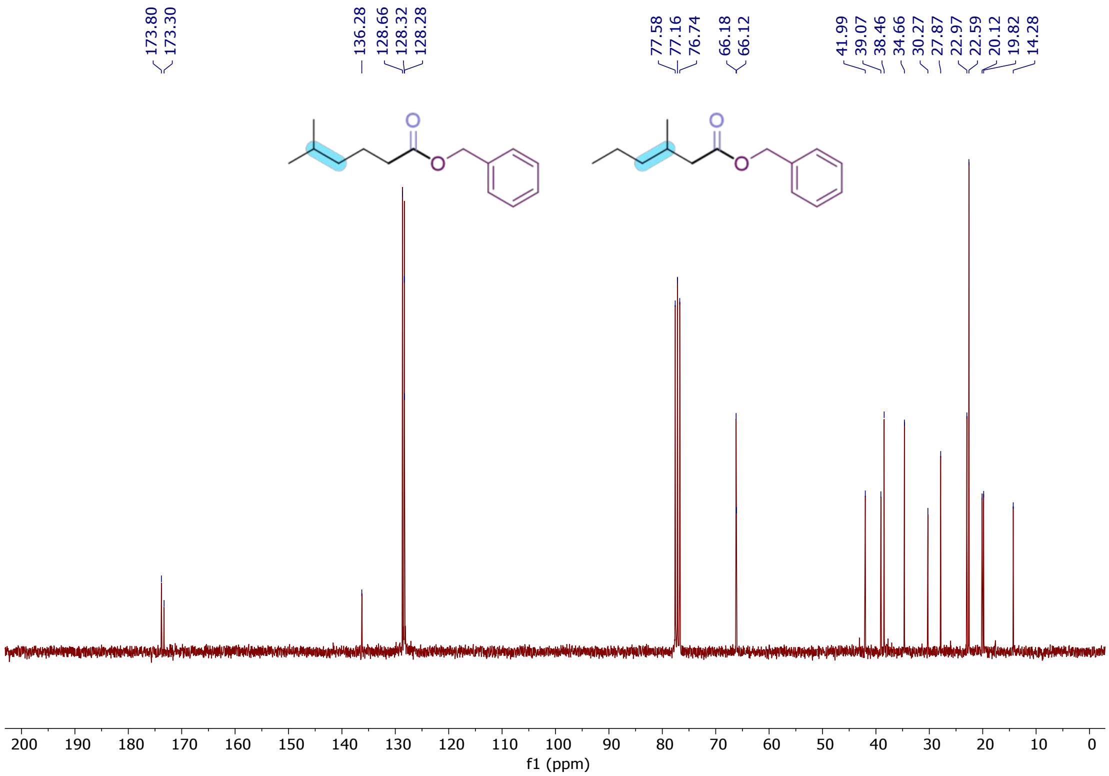

^{1}H NMR and ^{13}C NMR of **21**, *s₁* : s₂ = 1.5 : 1 (300 MHz, $CDCl_3$).

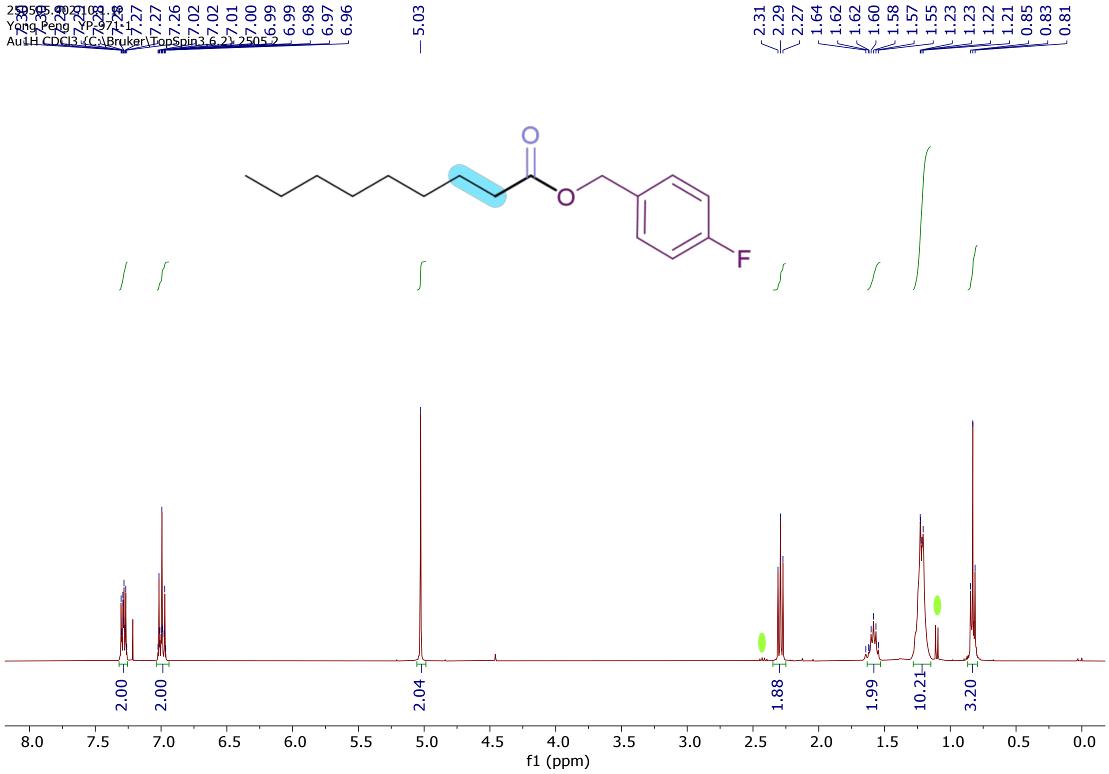

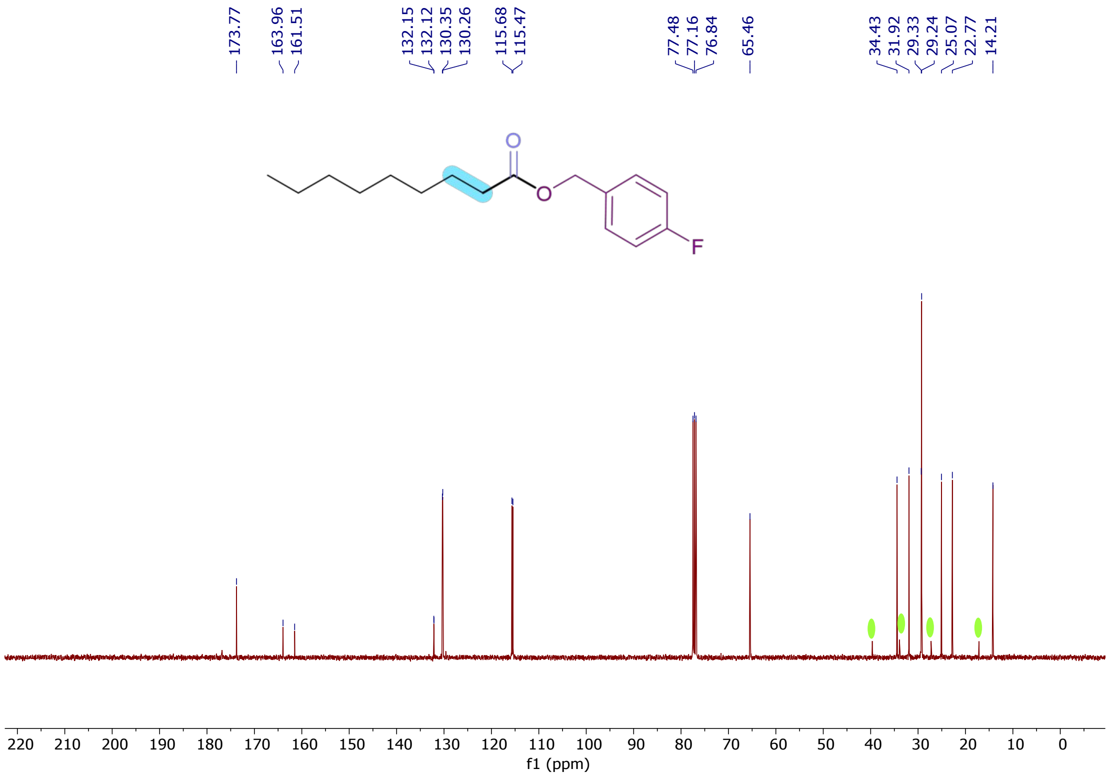

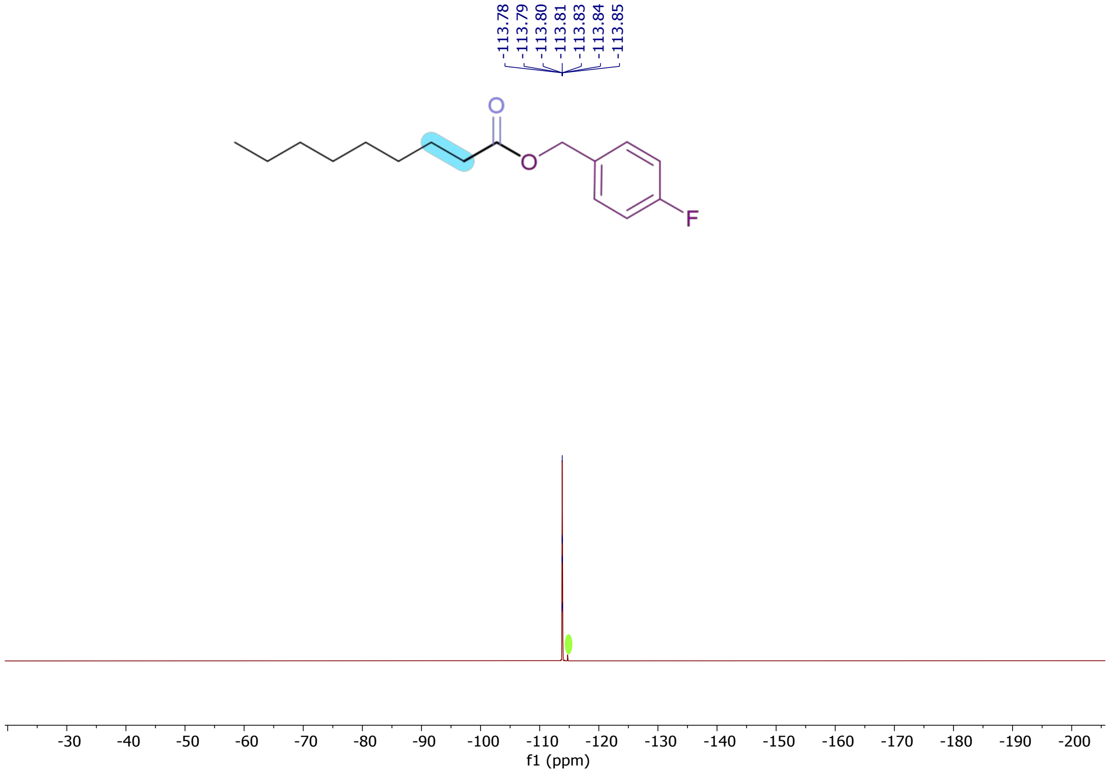

^{1}H NMR, ^{13}C NMR and ^{19}F NMR of **22**, *n/iso* = 88 : 12 (400 MHz, $CDCl_3$).

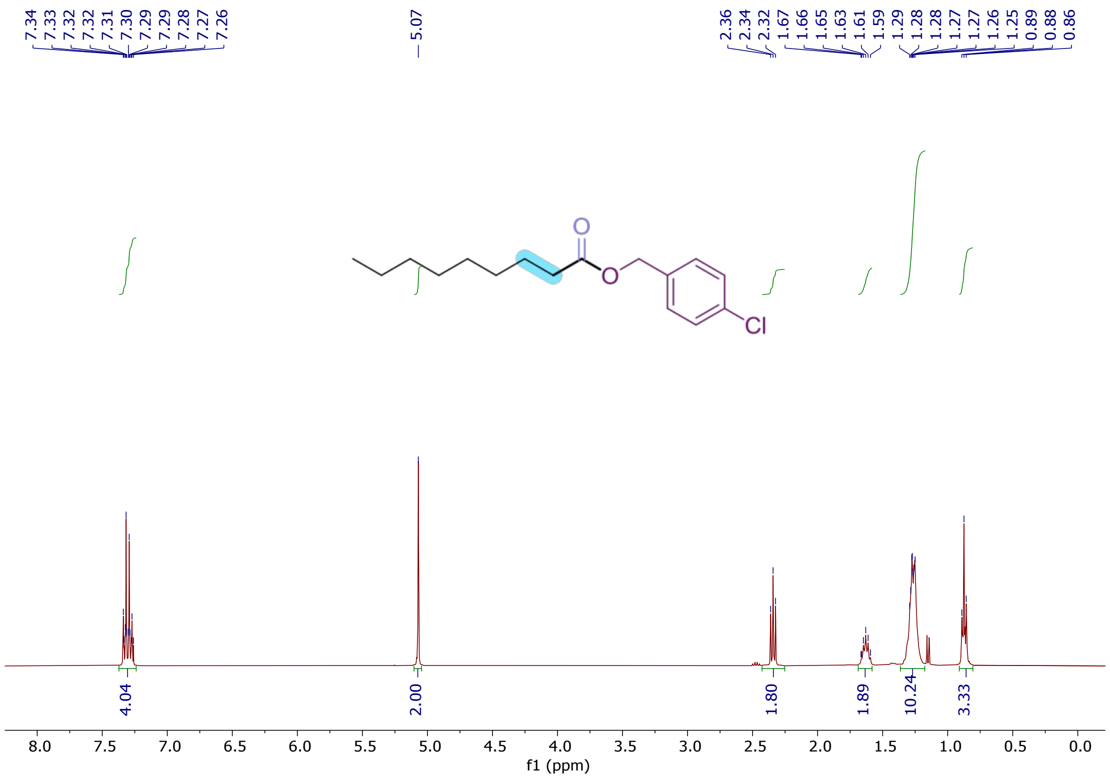

7.34
7.33
7.32
7.32
7.31
7.30
7.29
7.29
7.28
7.27
7.26
5.07
2.36
2.34
2.32
1.67
1.66
1.65
1.63
1.61
1.59
1.29
1.28
1.28
1.27
1.27
1.26
1.25
0.89
0.88
0.86
O
O
Cl
4.04
2.00
1.80
1.89
10.24
3.33
8.0 7.5 7.0 6.5 6.0 5.5 5.0 4.5 4.0 3.5 3.0 2.5 2.0 1.5 1.0 0.5 0.0
f1 (ppm)

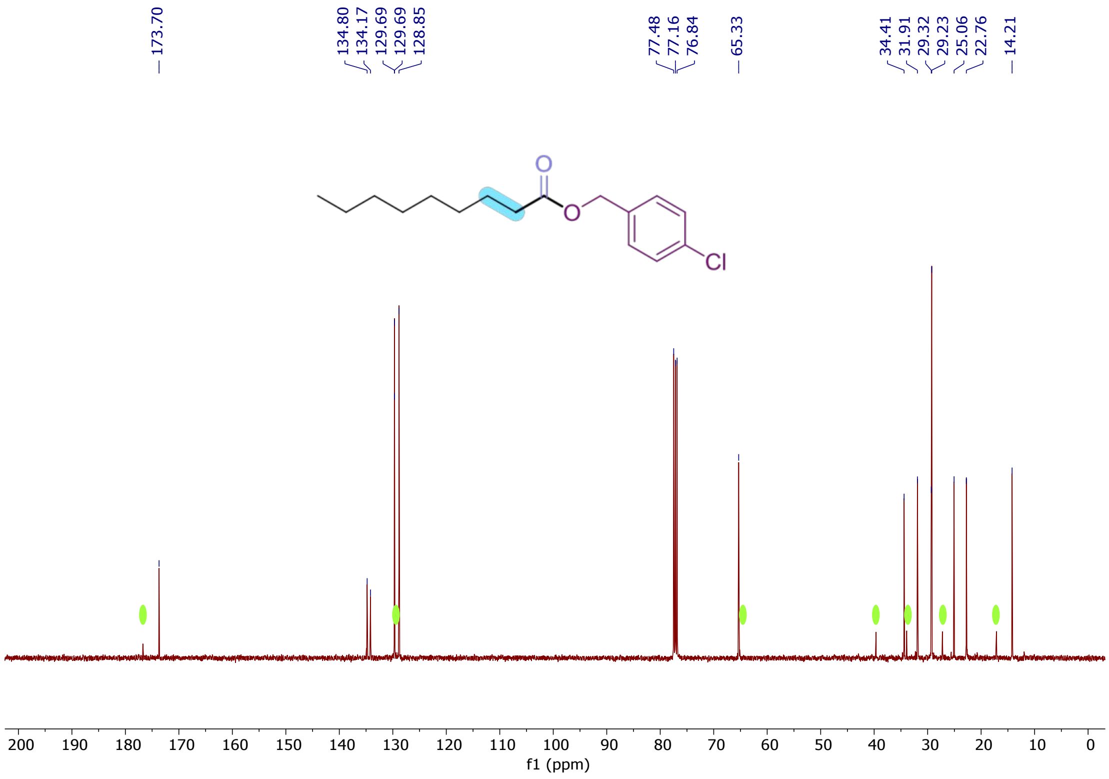

^{1}H NMR and ^{13}C NMR of **23**, *n/iso* = 87 : 13 (400 MHz, $CDCl_3$).

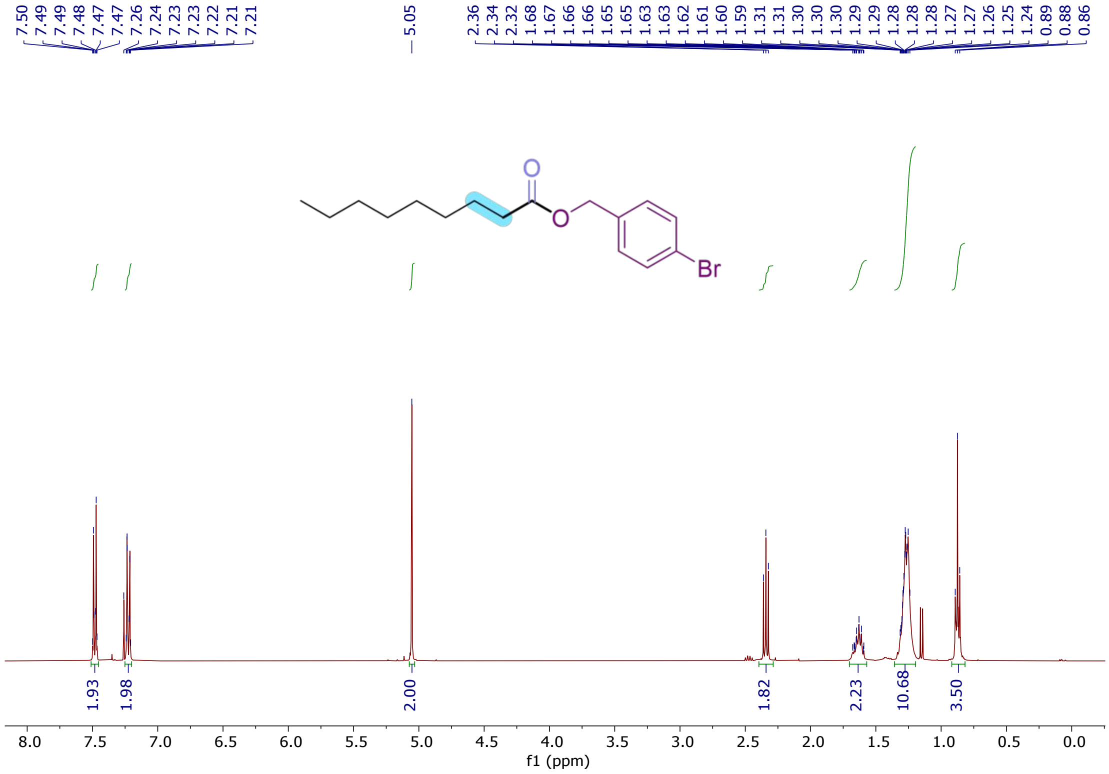

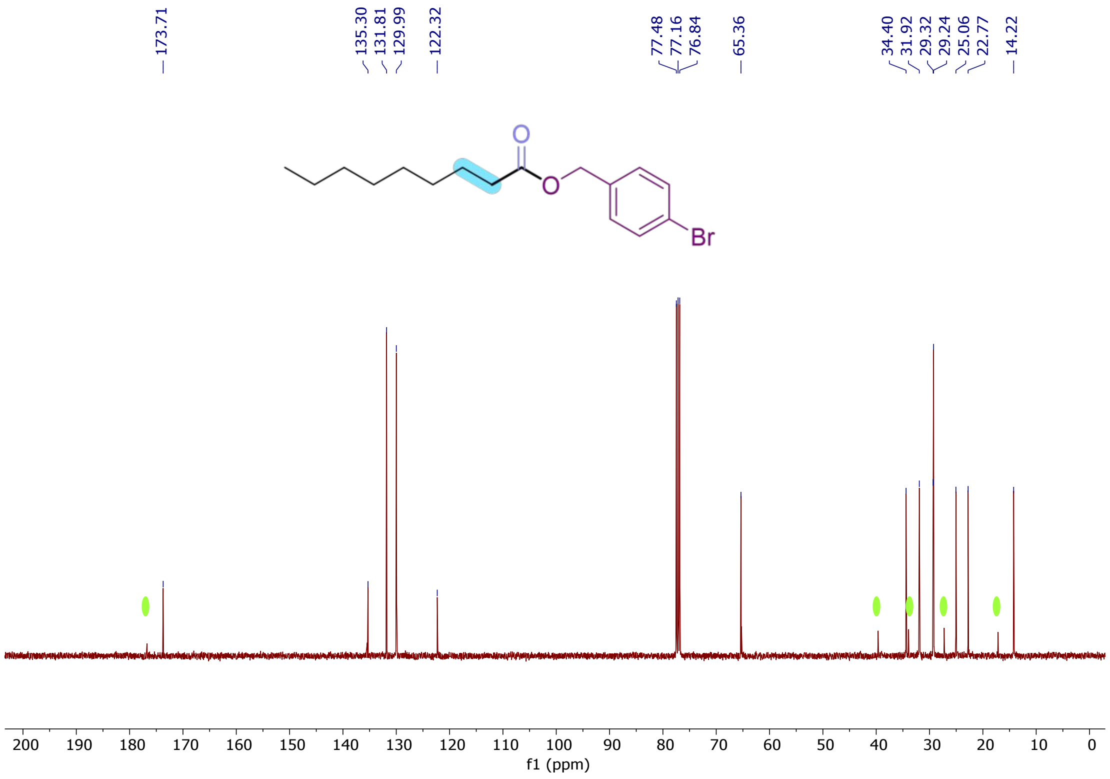

^{1}H NMR and ^{13}C NMR of **24**, *n/iso* = 87 : 13 (400 MHz, $CDCl_3$).

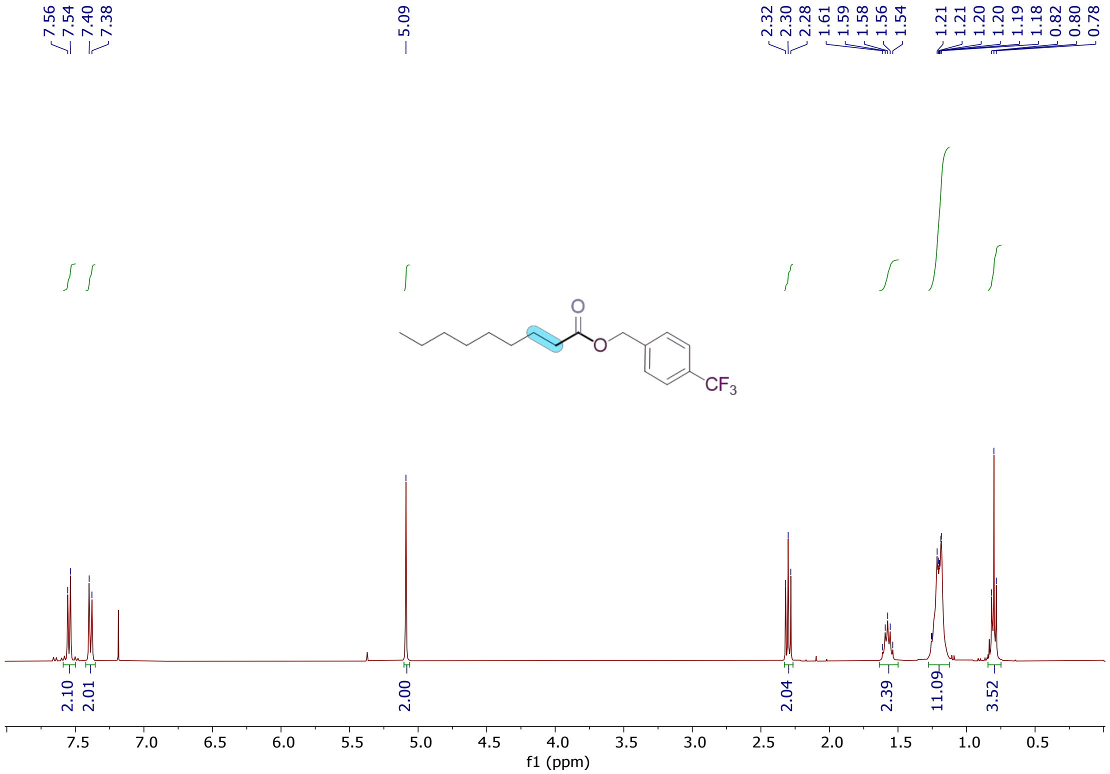

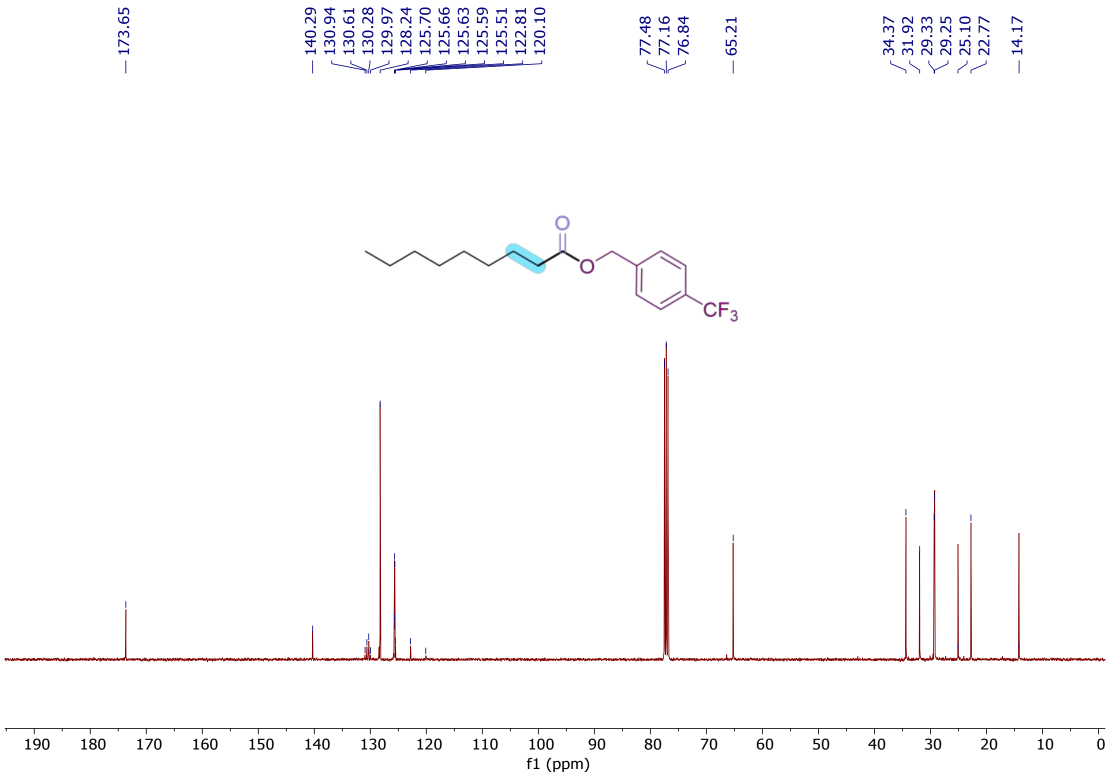

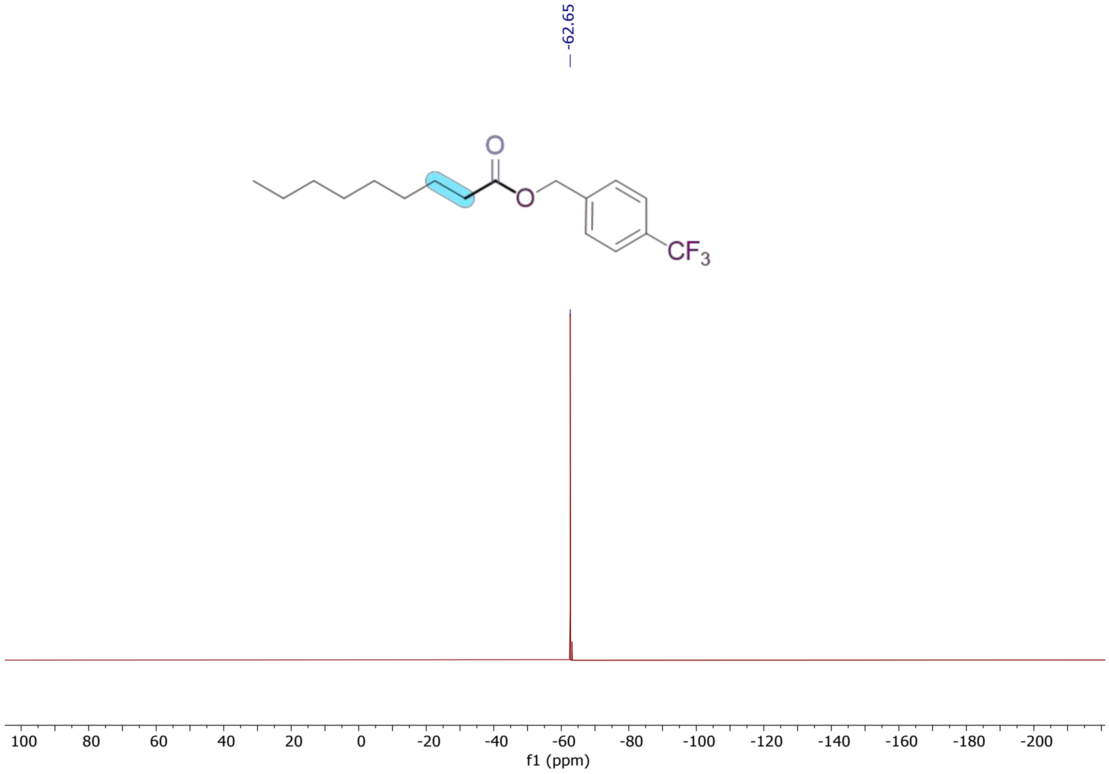

^{1}H NMR, ^{13}C NMR and ^{19}F NMR of **25**, *n/iso* = 90 : 10 (400 MHz, $CDCl_3$).

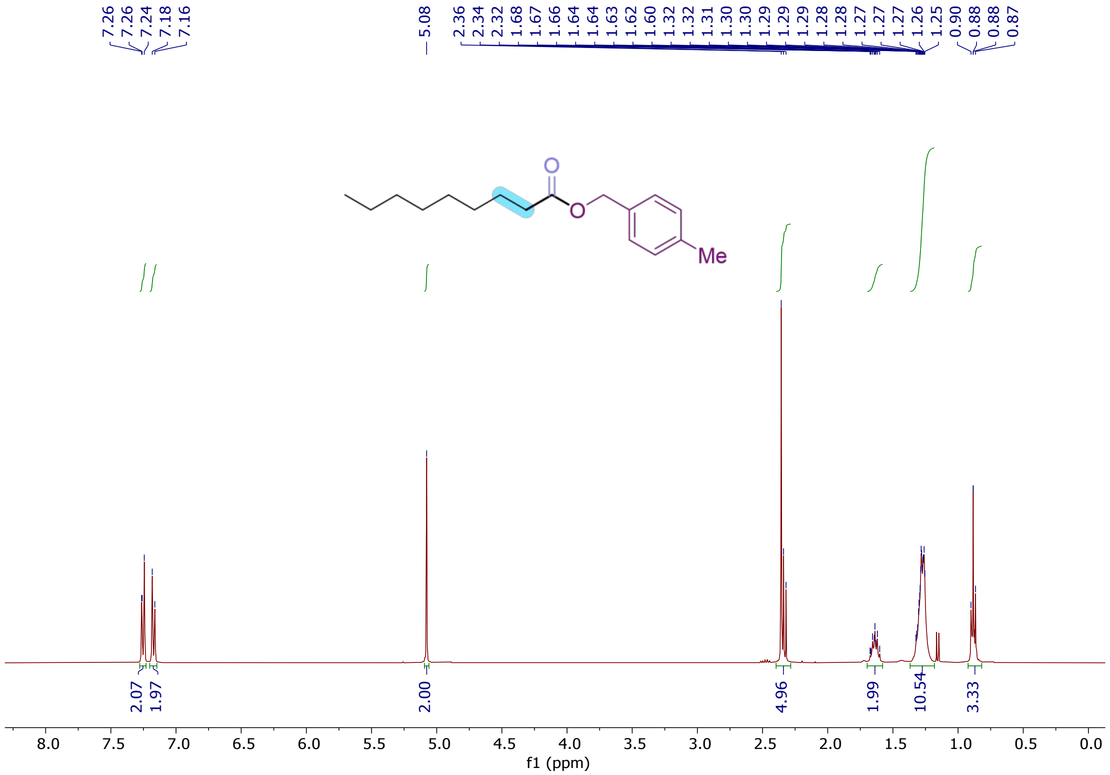
7.26
7.26
7.24
7.18
7.16
5.08
2.36
2.34
2.32
1.68
1.67
1.66
1.64
1.64
1.63
1.62
1.60
1.32
1.32
1.31
1.30
1.30
1.29
1.29
1.29
1.28
1.28
1.27
1.27
1.27
1.26
1.25
0.90
0.88
0.88
0.87
O
O
Me
2.07
1.97
2.00
4.96
1.99
10.54
3.33
8.0 7.5 7.0 6.5 6.0 5.5 5.0 4.5 4.0 3.5 3.0 2.5 2.0 1.5 1.0 0.5 0.0
f1 (ppm)

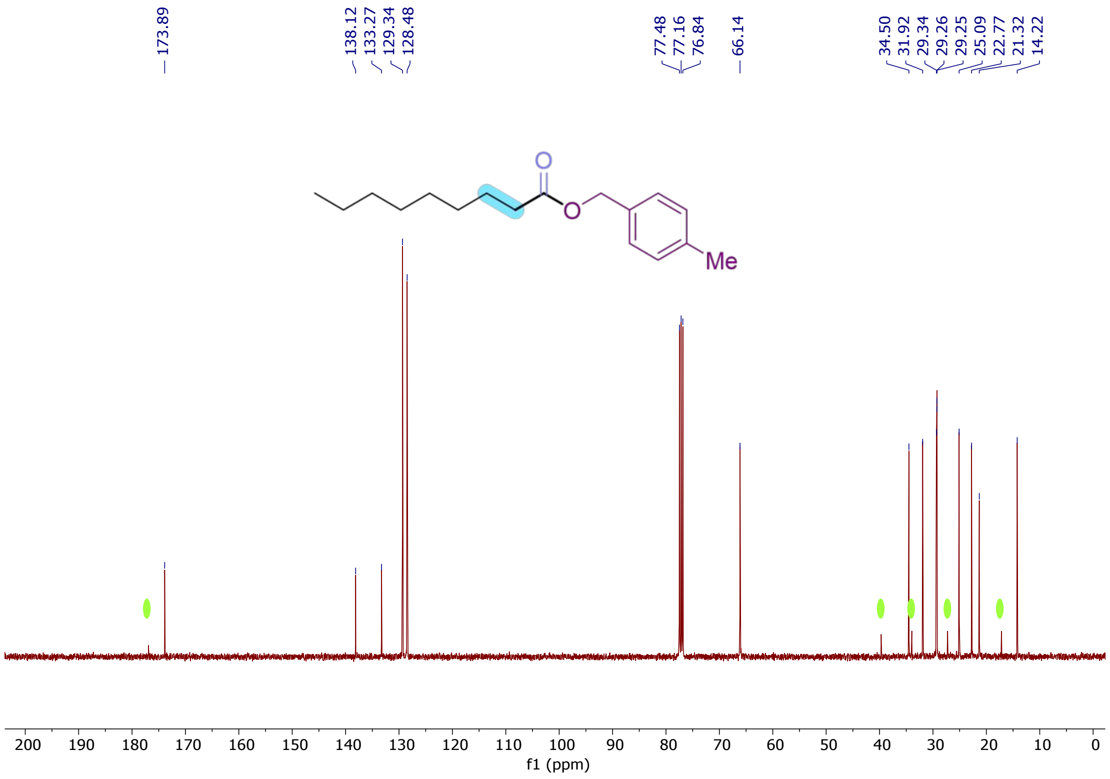

^{1}H NMR and ^{13}C NMR of **26**, *n/iso* = 89 : 11 (400 MHz, $CDCl_3$).

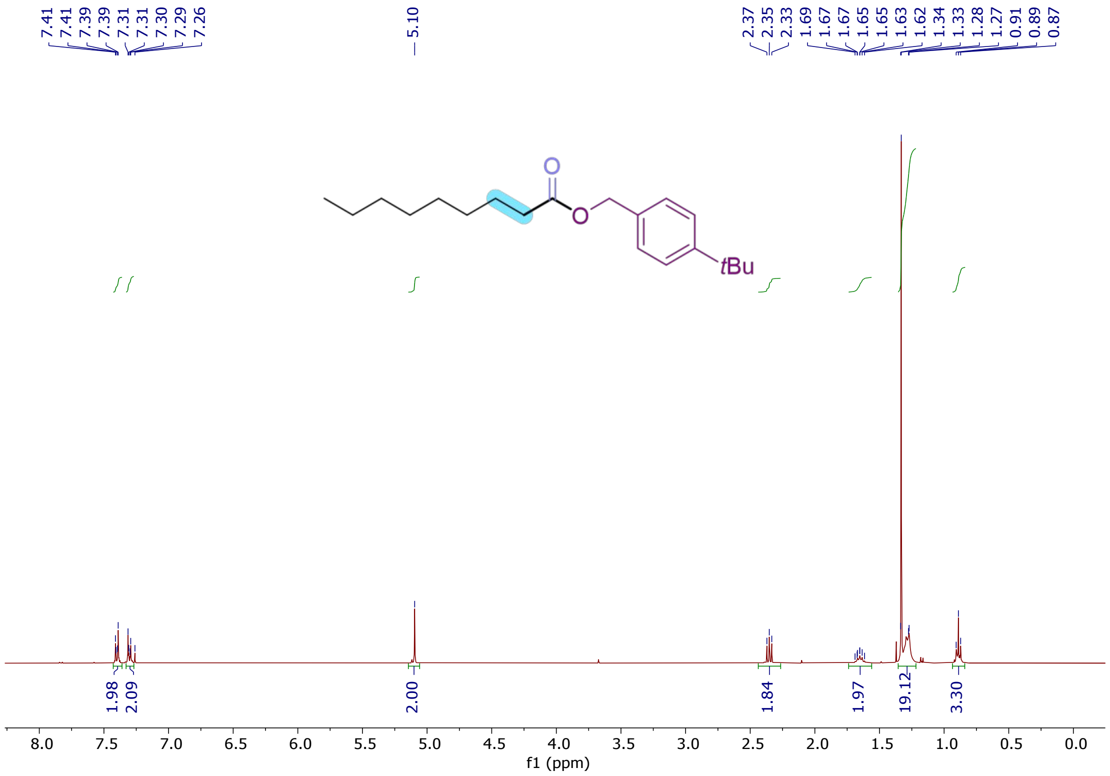

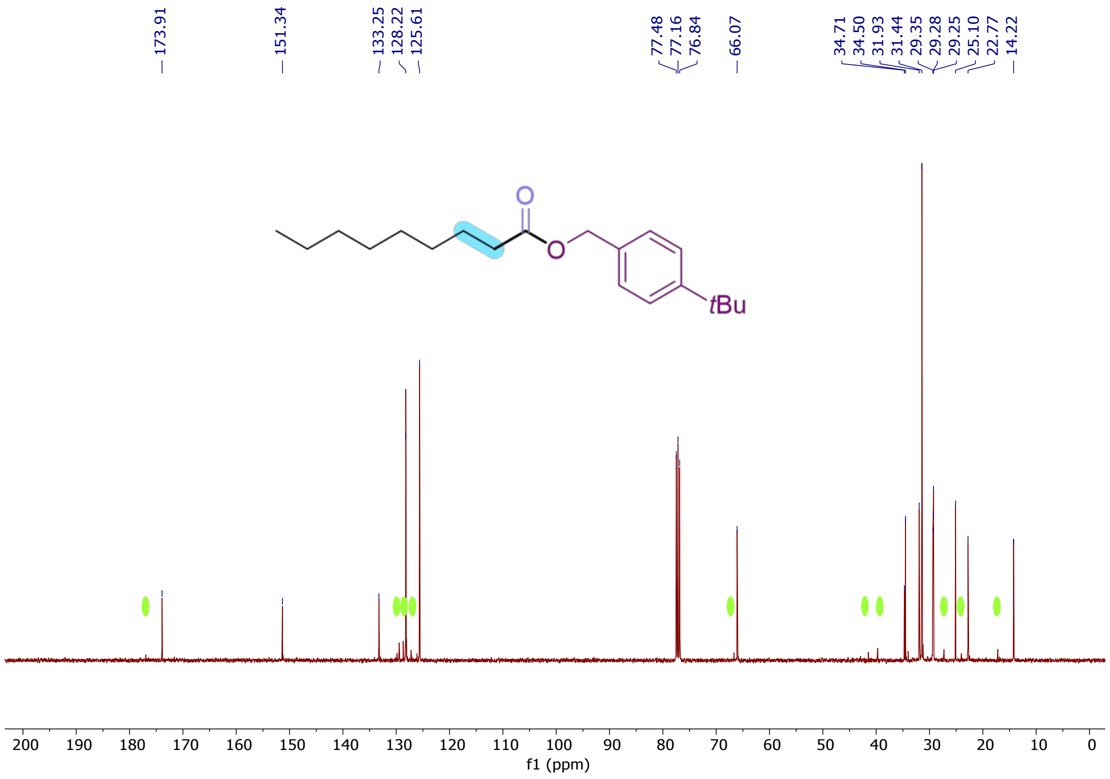

^{1}H NMR and ^{13}C NMR of **27**, *n/iso* = 93 : 7 (400 MHz, $CDCl_3$).

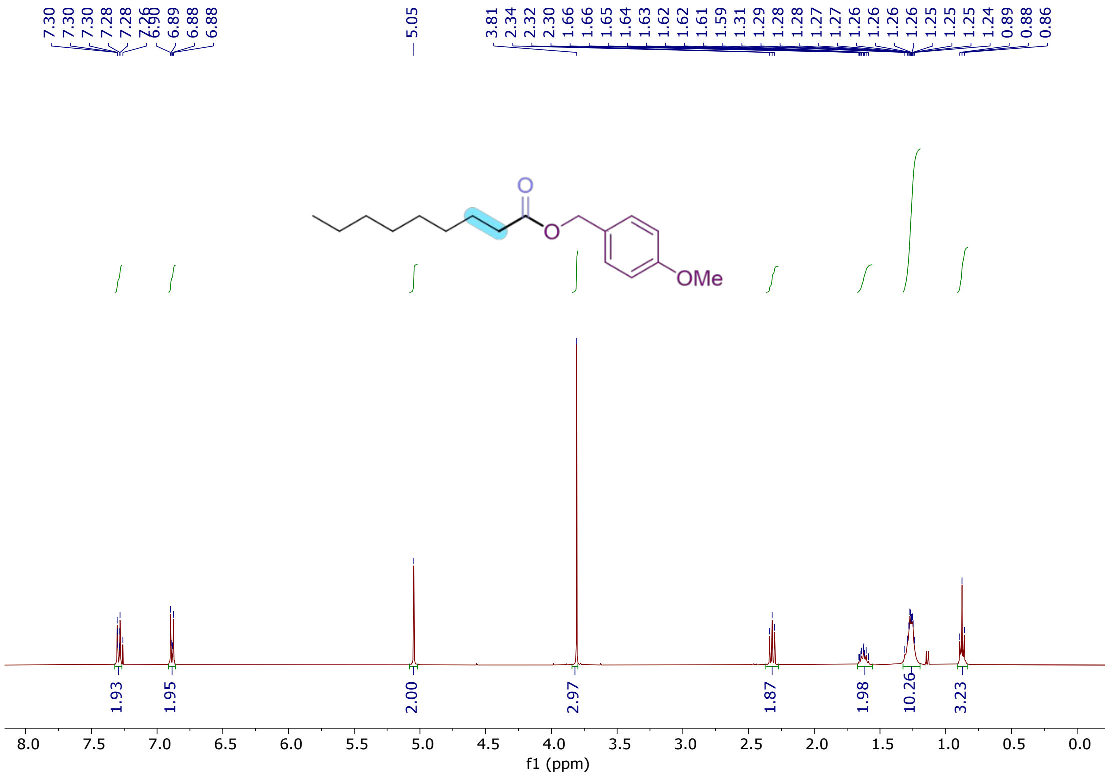

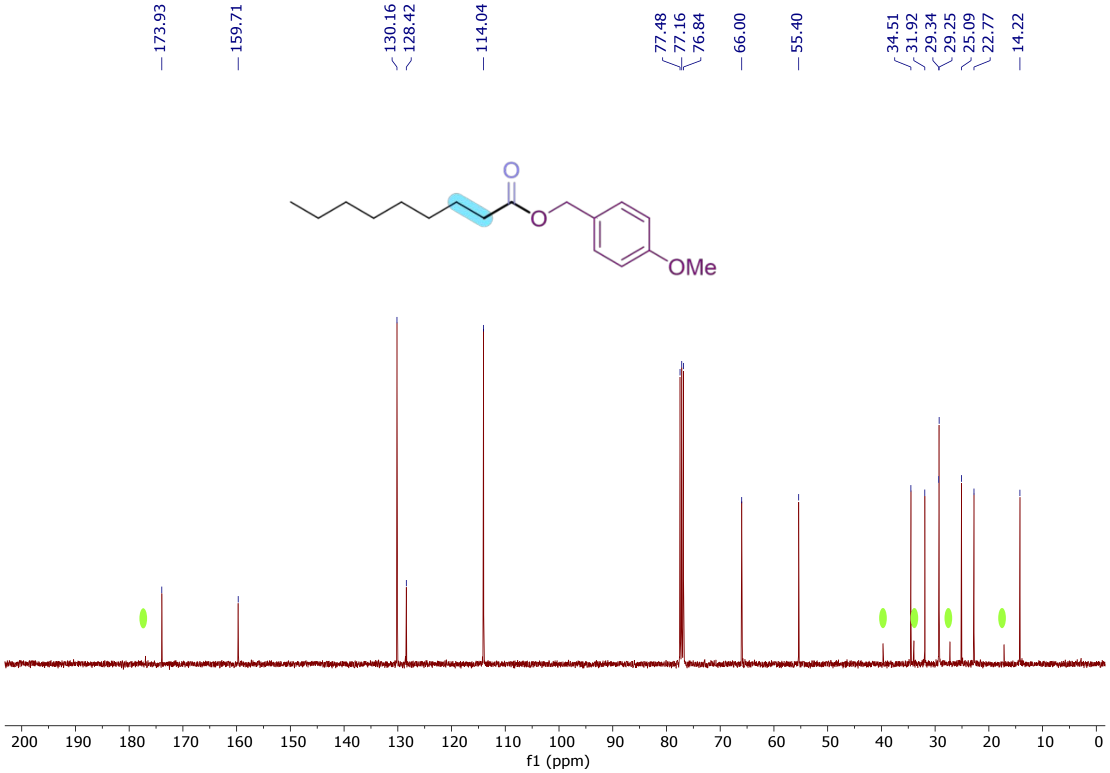

^{1}H NMR and ^{13}C NMR of **28**, *n/iso* = 89 : 11 (400 MHz, $CDCl_3$).

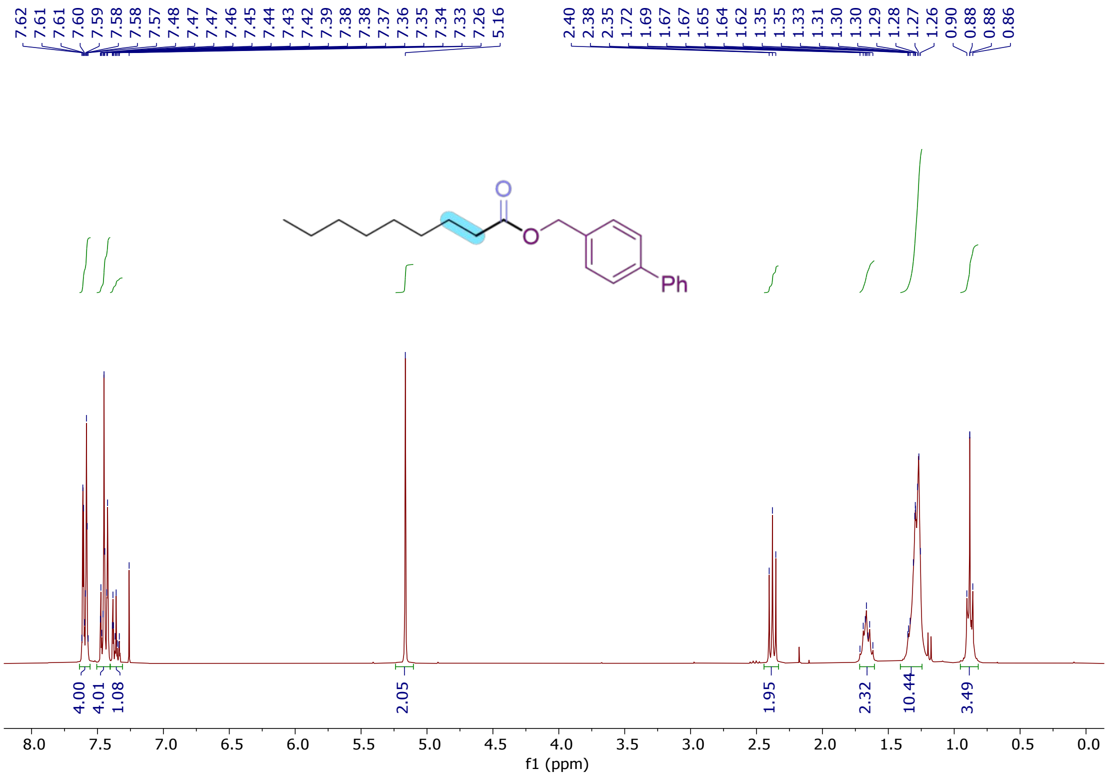

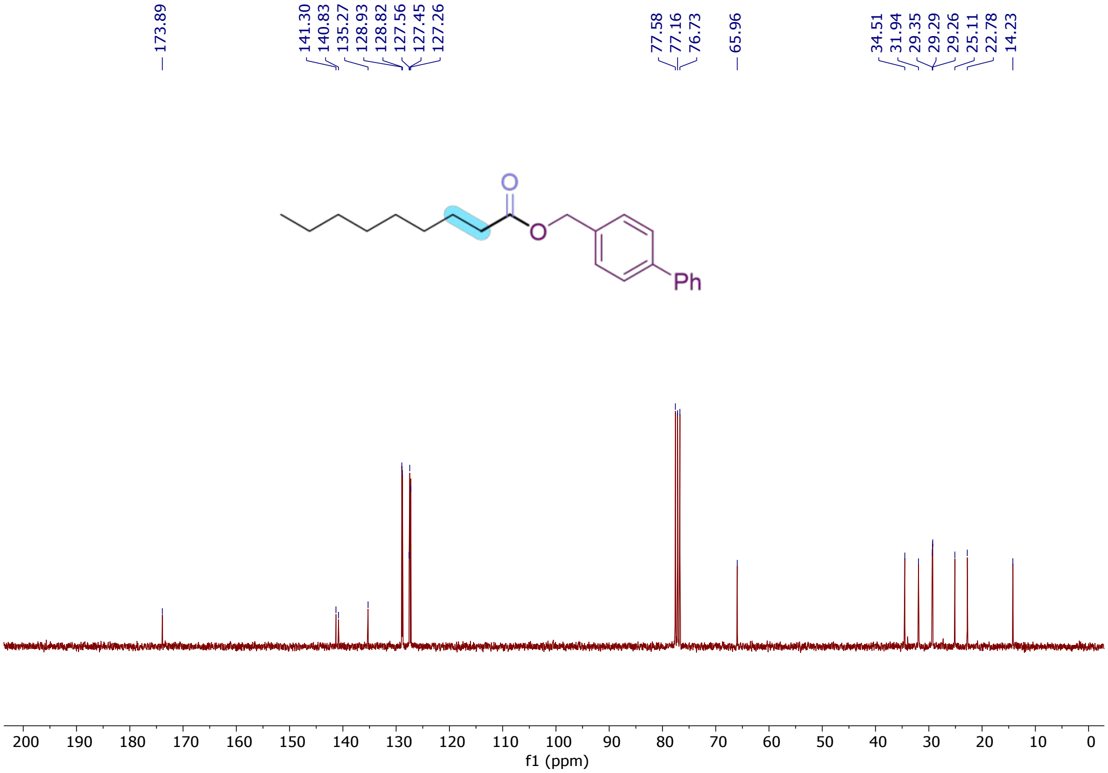

^{1}H NMR and ^{13}C NMR of **29**, *n/iso* = 90 : 10 (300 MHz, $CDCl_3$).

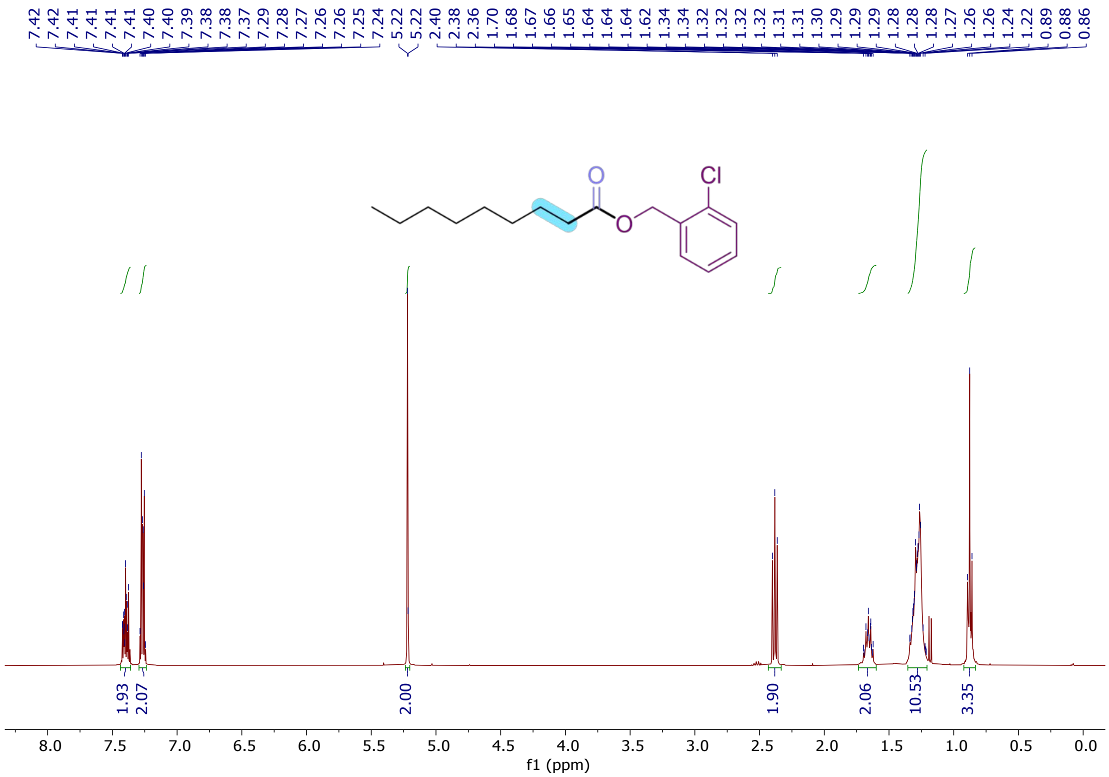

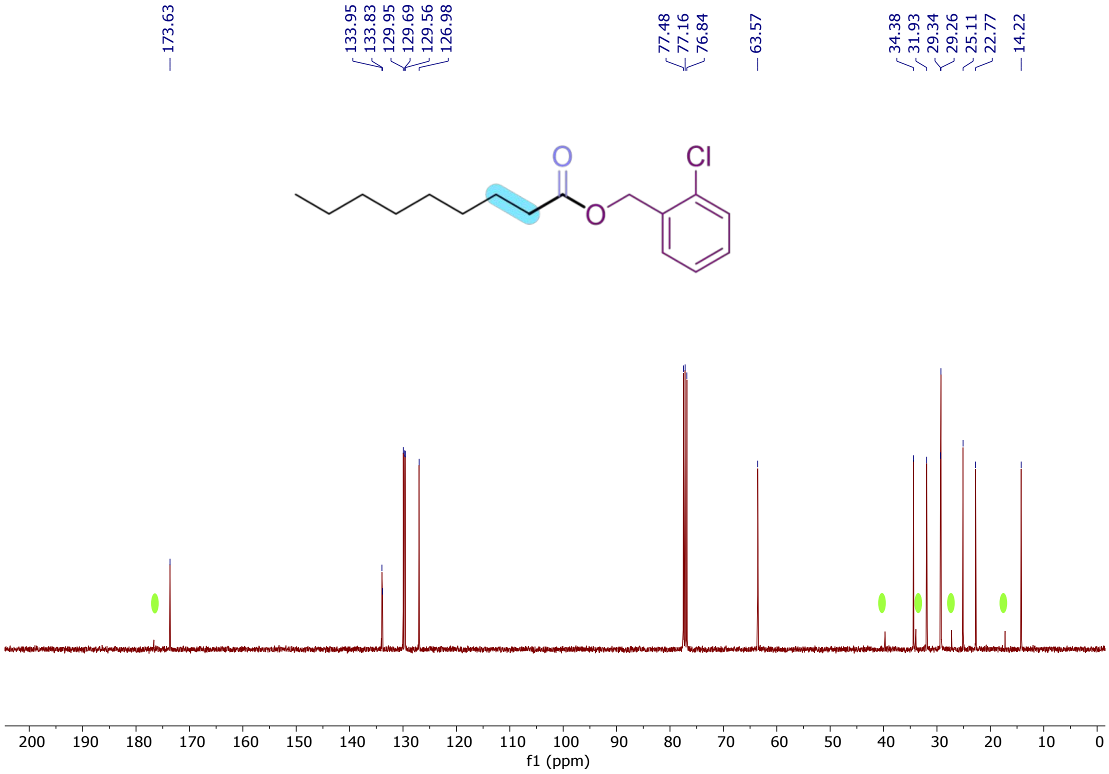

^{1}H NMR and ^{13}C NMR of **30**, *n/iso* = 90 : 10 (400 MHz, $CDCl_3$).

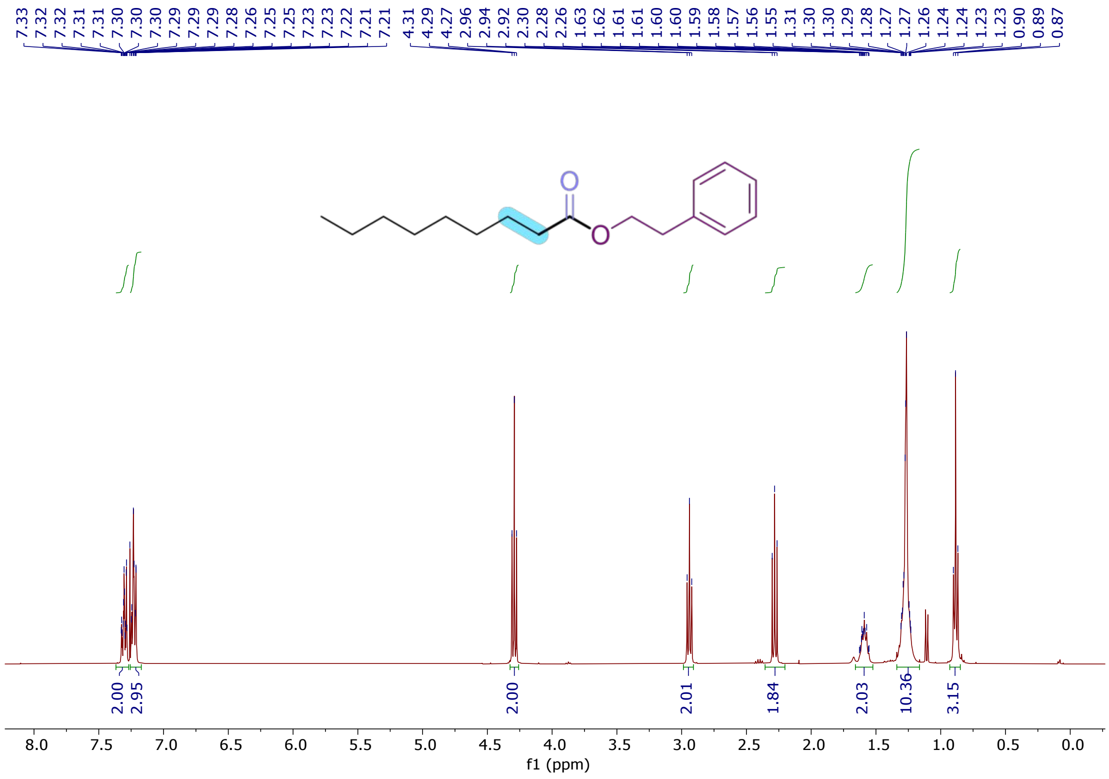

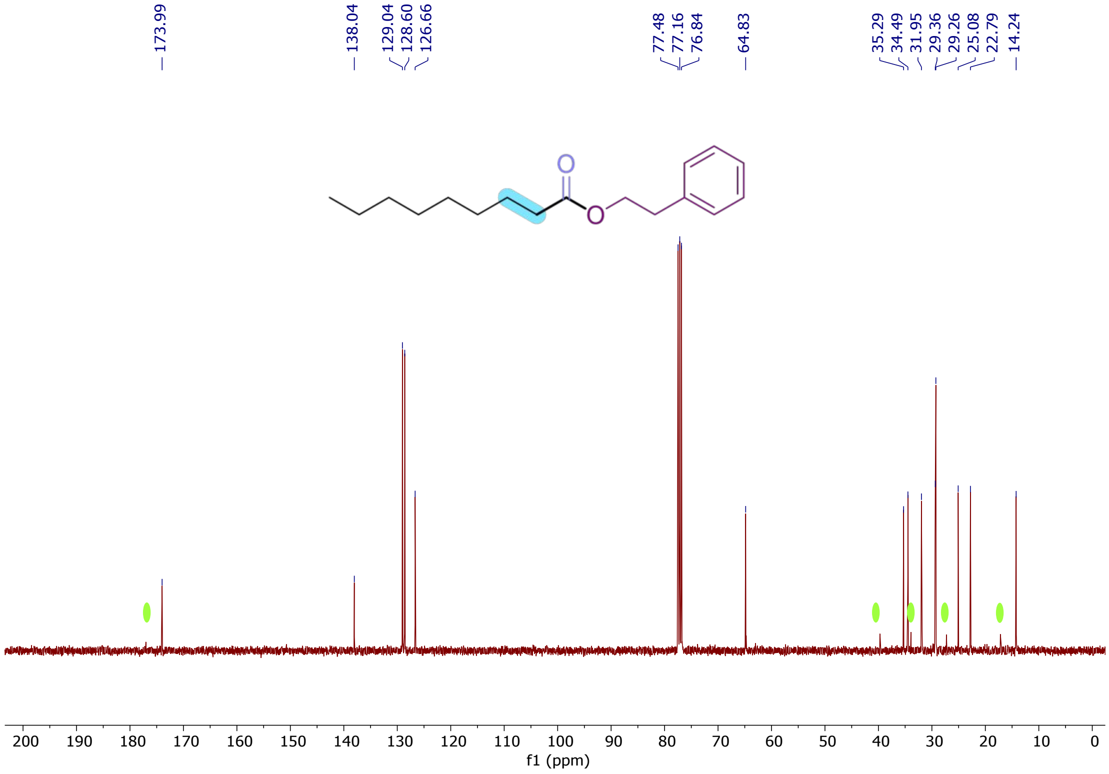

^{1}H NMR and ^{13}C NMR of **31**, *n/iso* = 90 : 10 (400 MHz, $CDCl_3$).

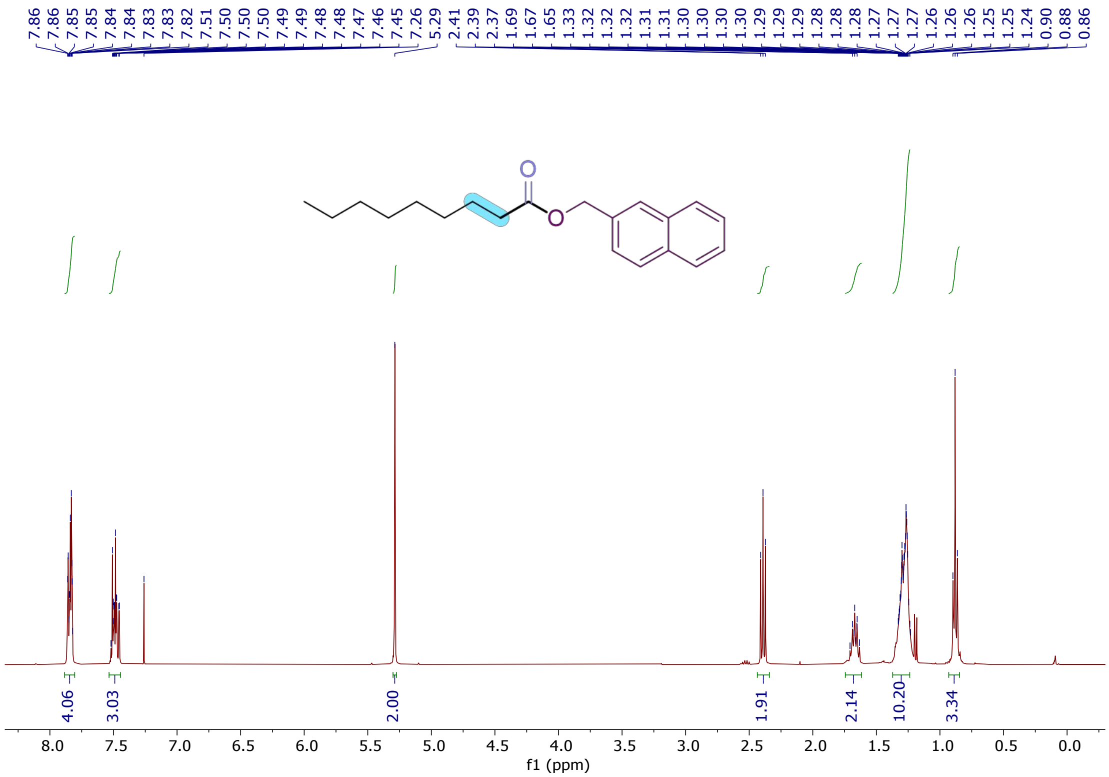

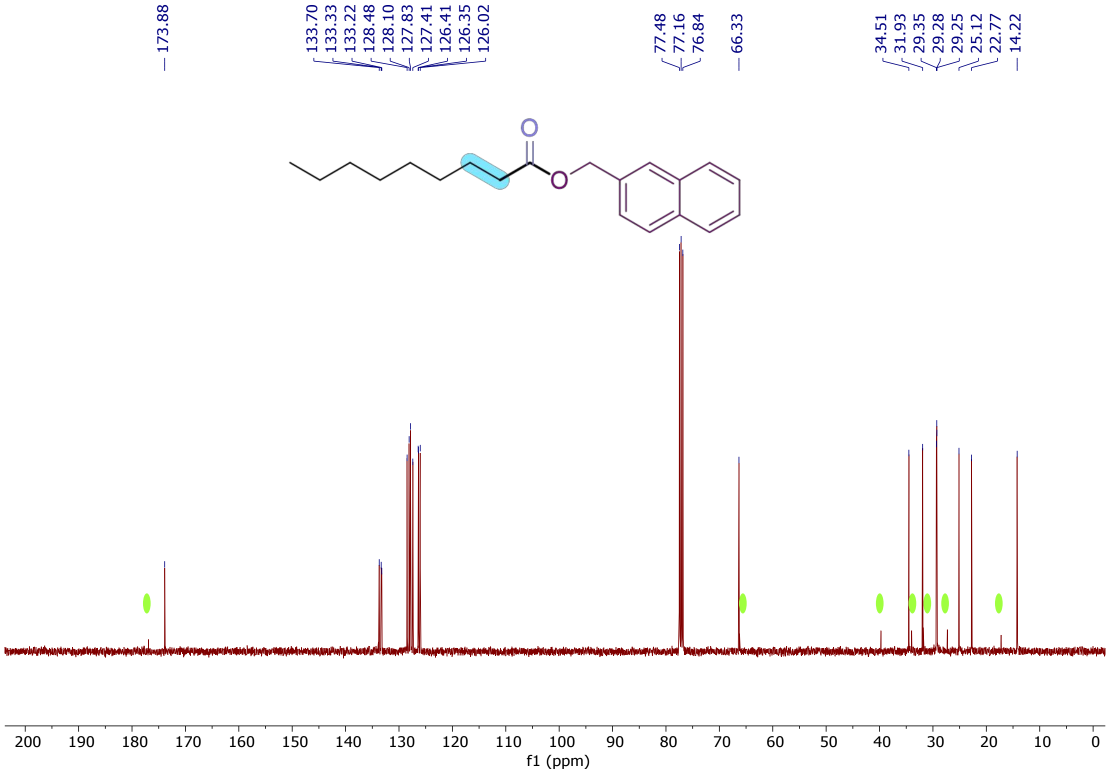

^{1}H NMR and ^{13}C NMR of **32**, *n/iso* = 90 : 10 (400 MHz, $CDCl_3$).

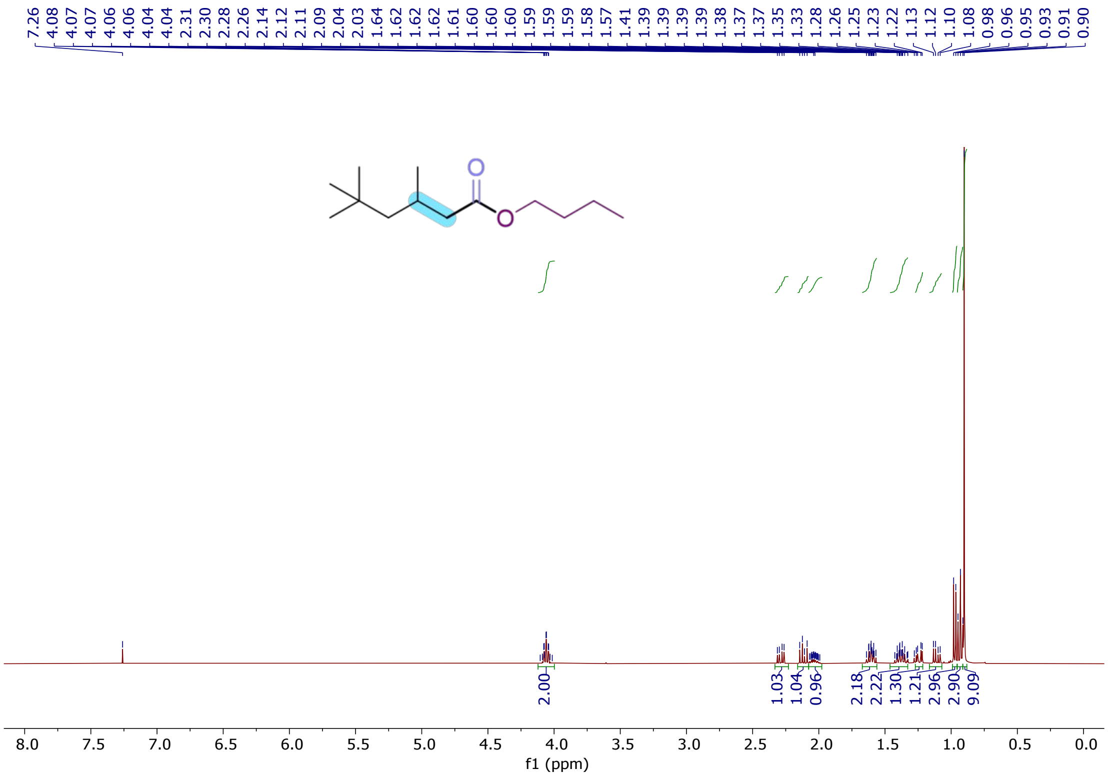

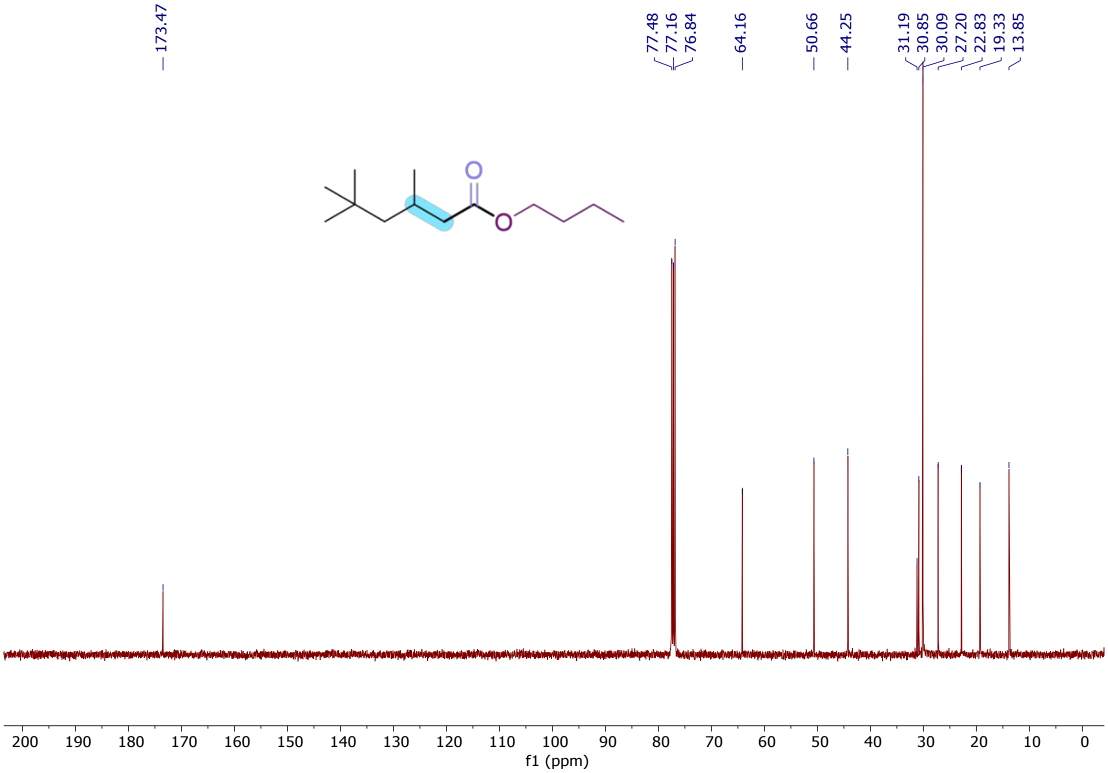

^1H NMR and ^{13}C NMR of **33**, (400 MHz, $CDCl_3$).

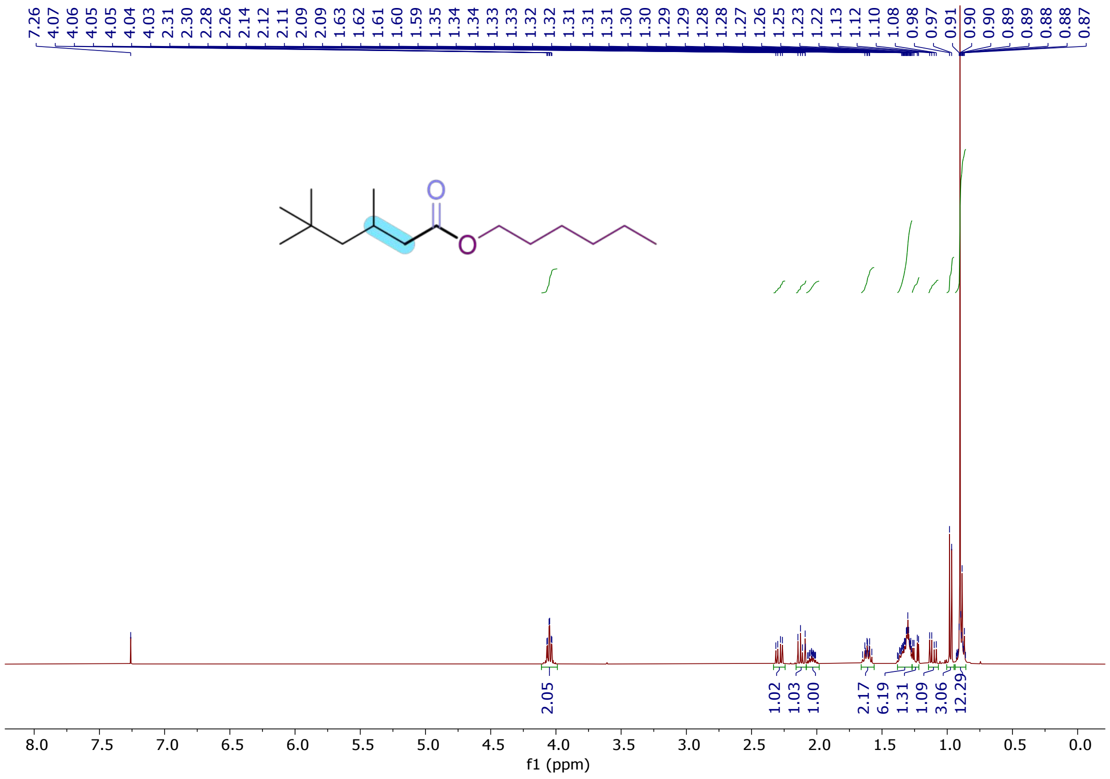

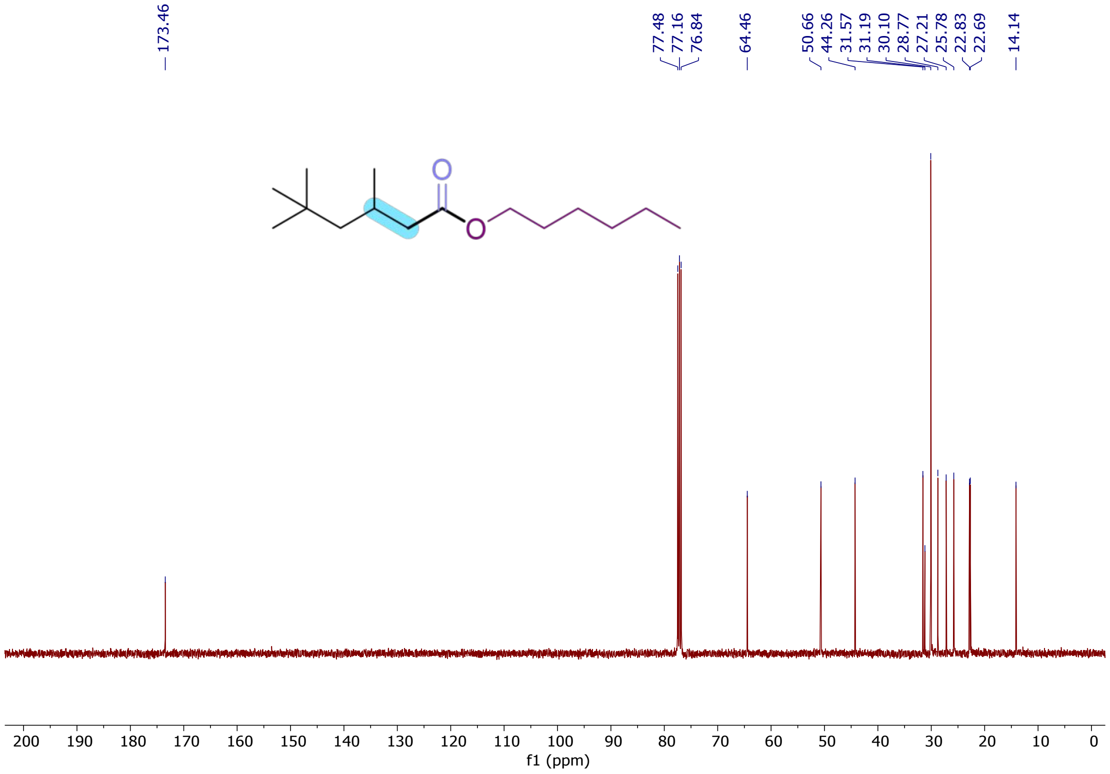

^{1}H NMR and ^{13}C NMR of **34**, (400 MHz, $CDCl_3$).

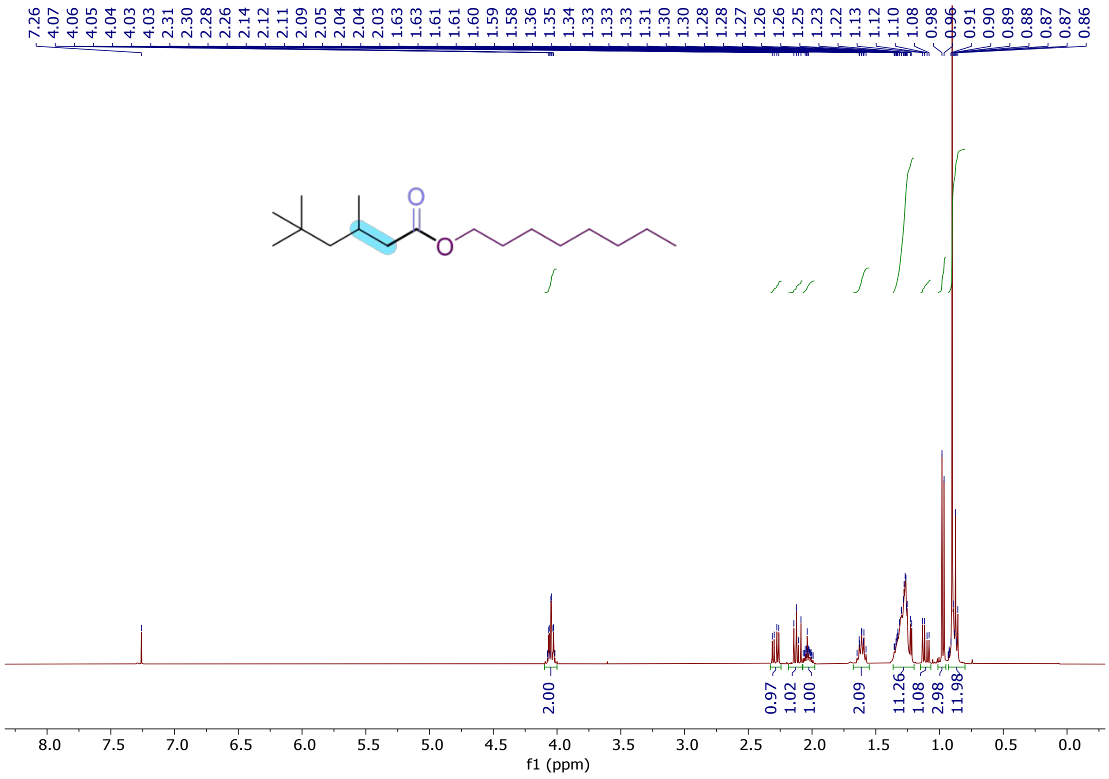

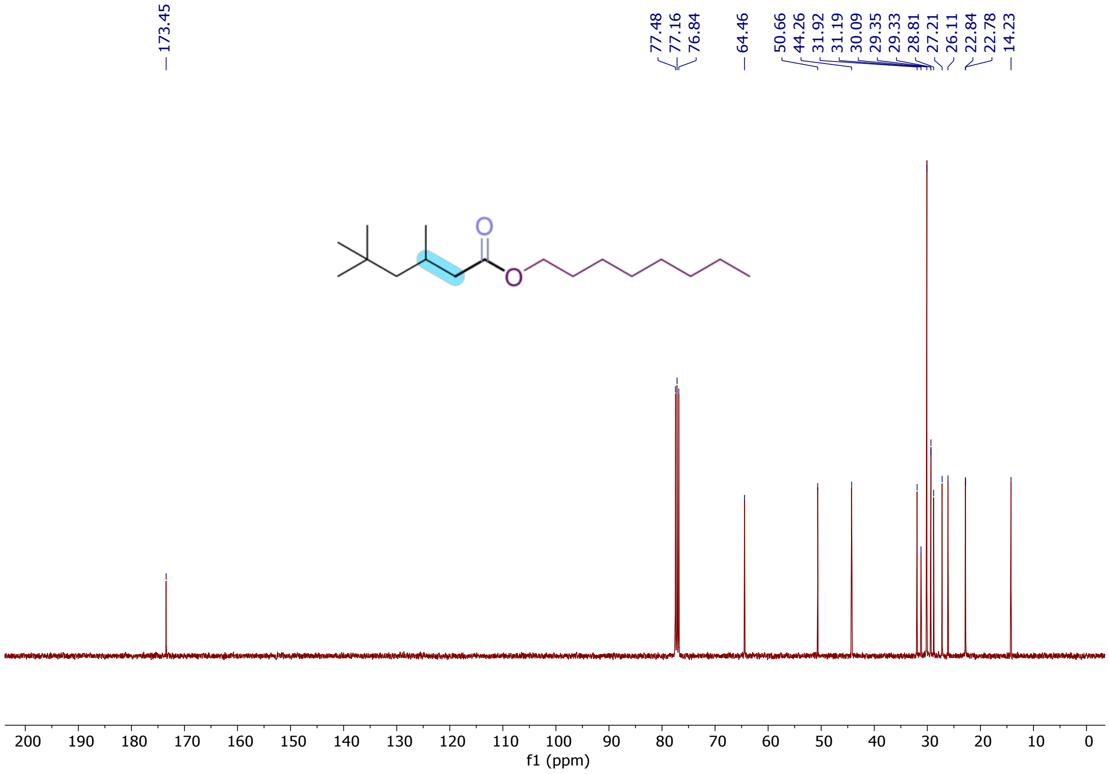

^1H NMR and ^{13}C NMR of **35**, (400 MHz, $CDCl_3$).

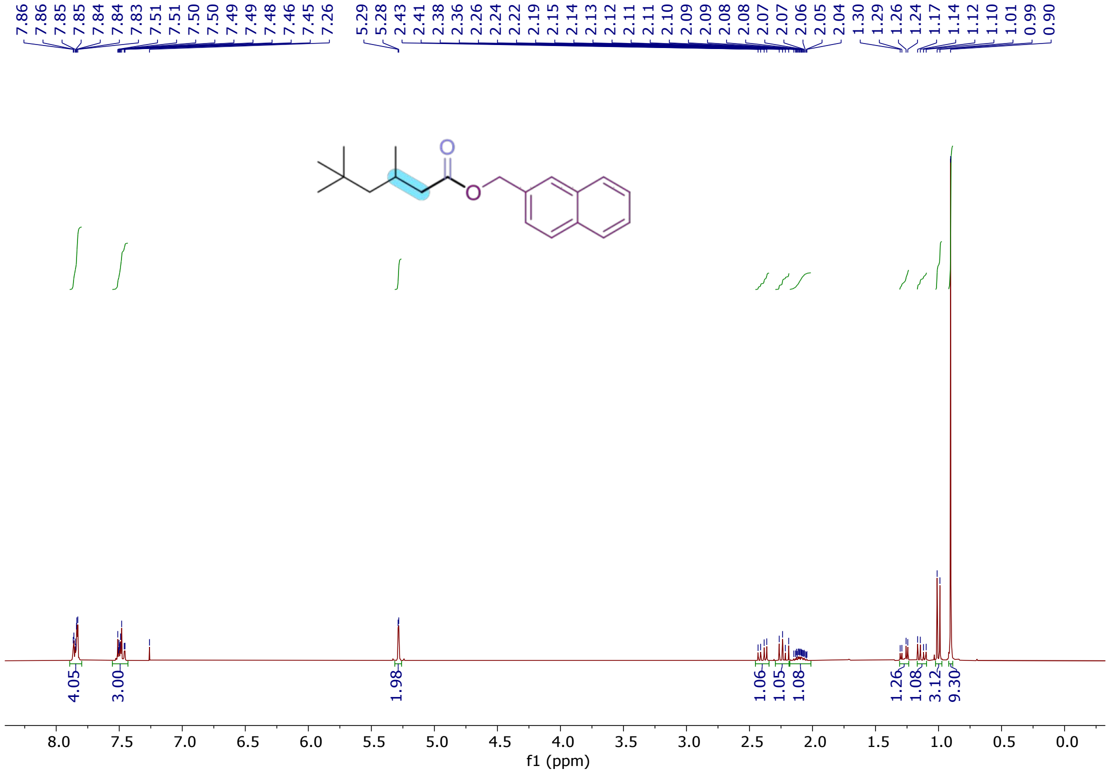

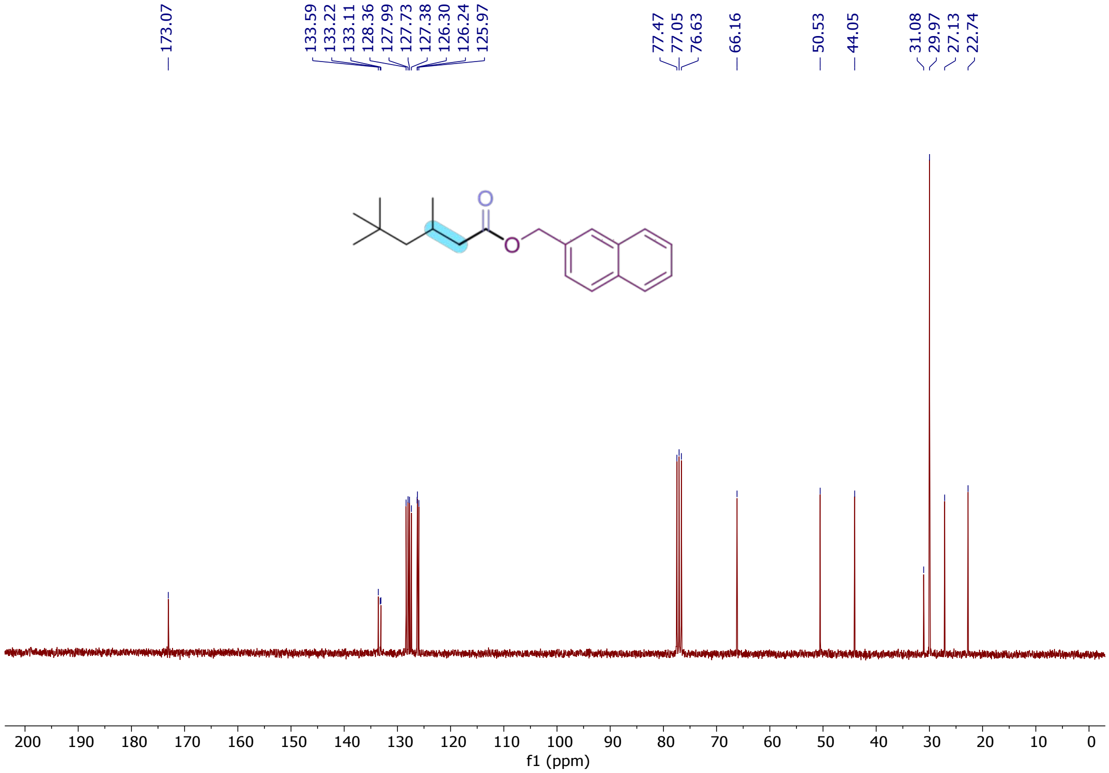

^{1}H NMR and ^{13}C NMR of **36**, (300 MHz, $CDCl_3$).

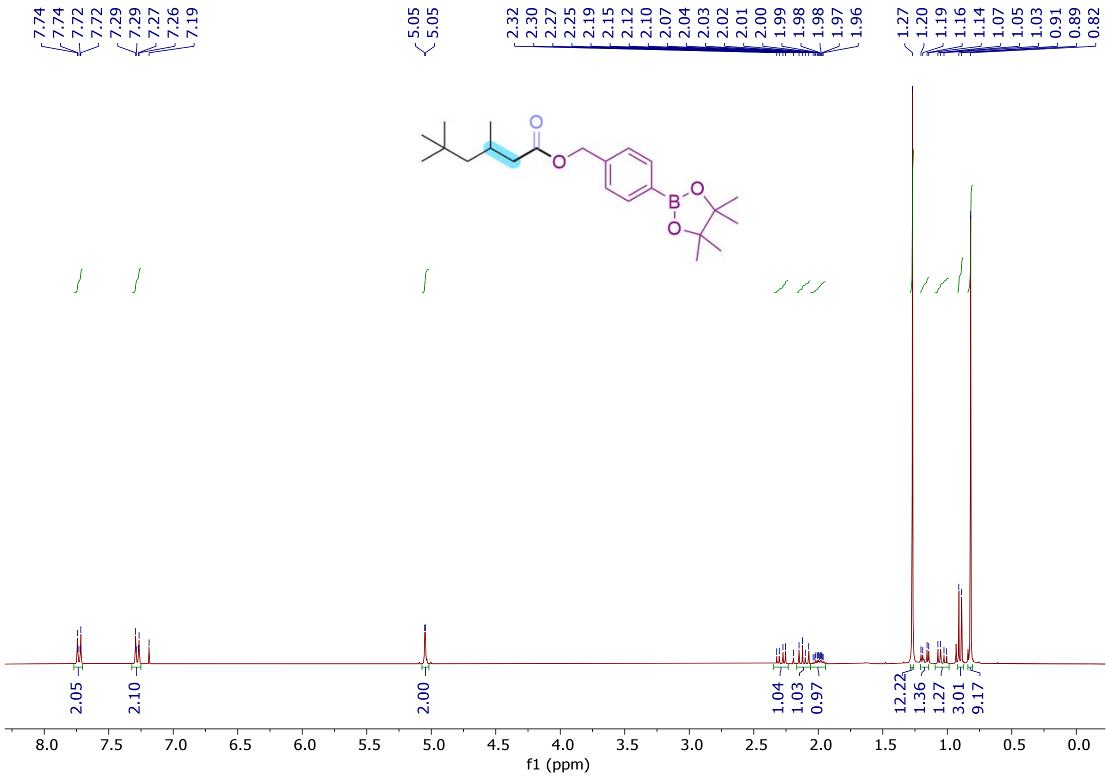

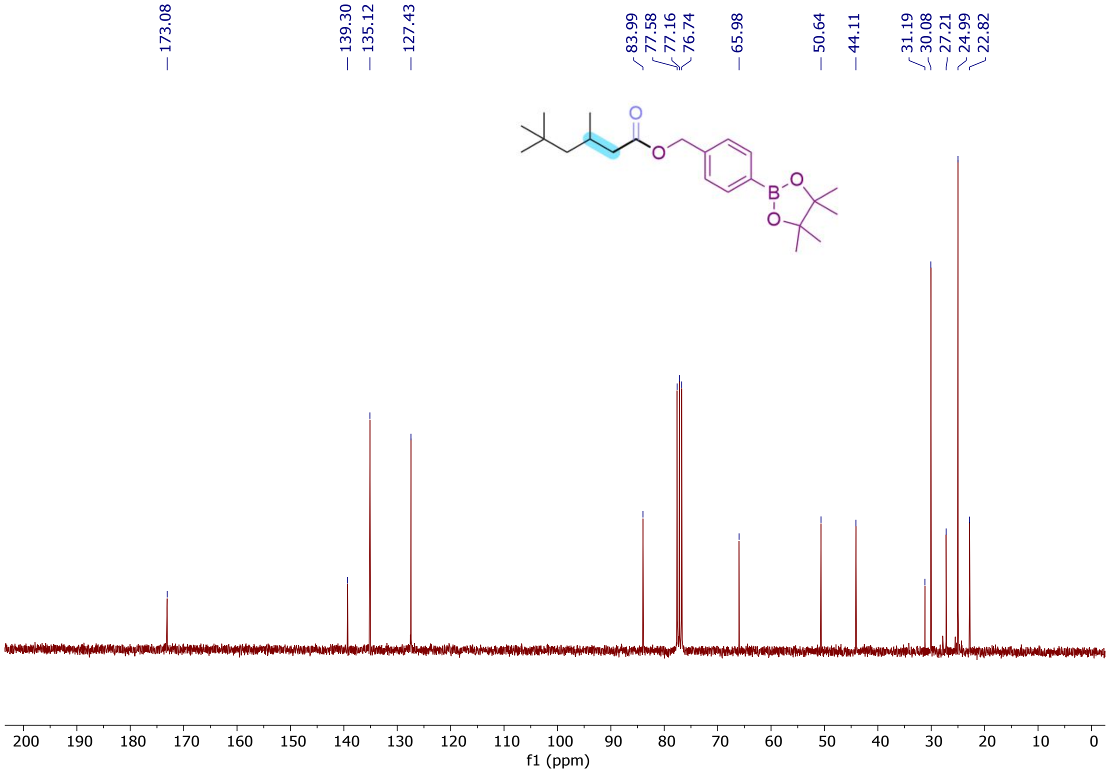

^1H NMR and ^{13}C NMR of **37**, (300 MHz, $CDCl_3$).

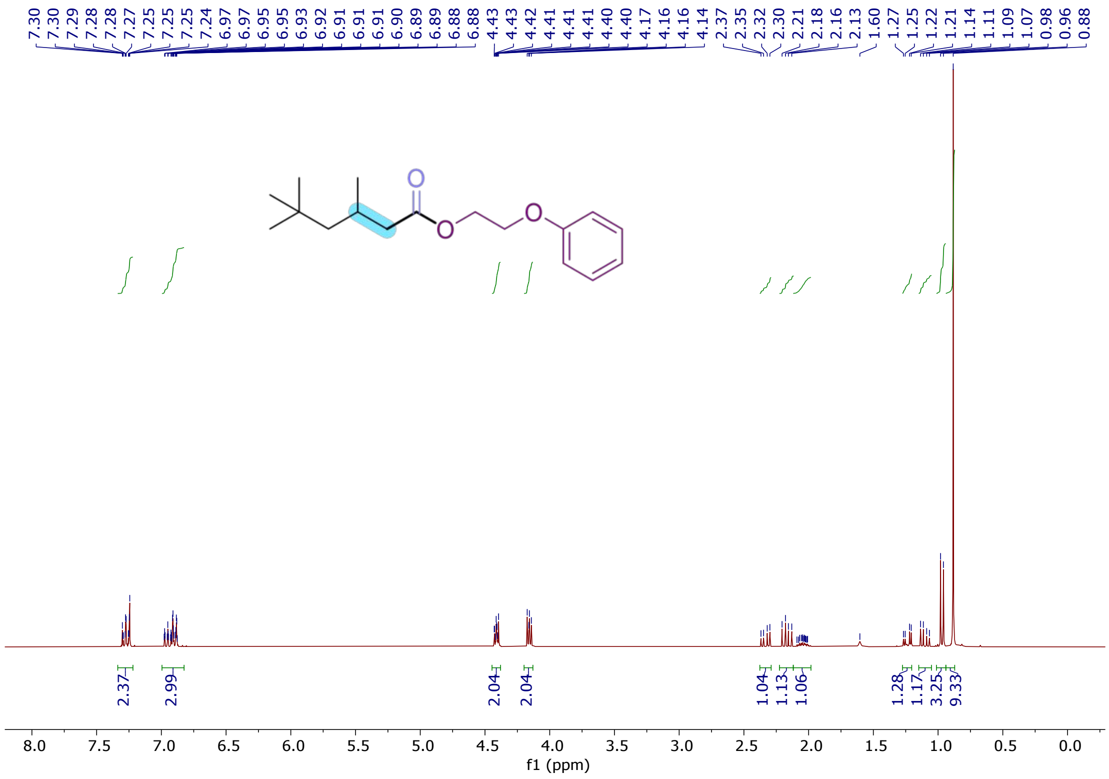

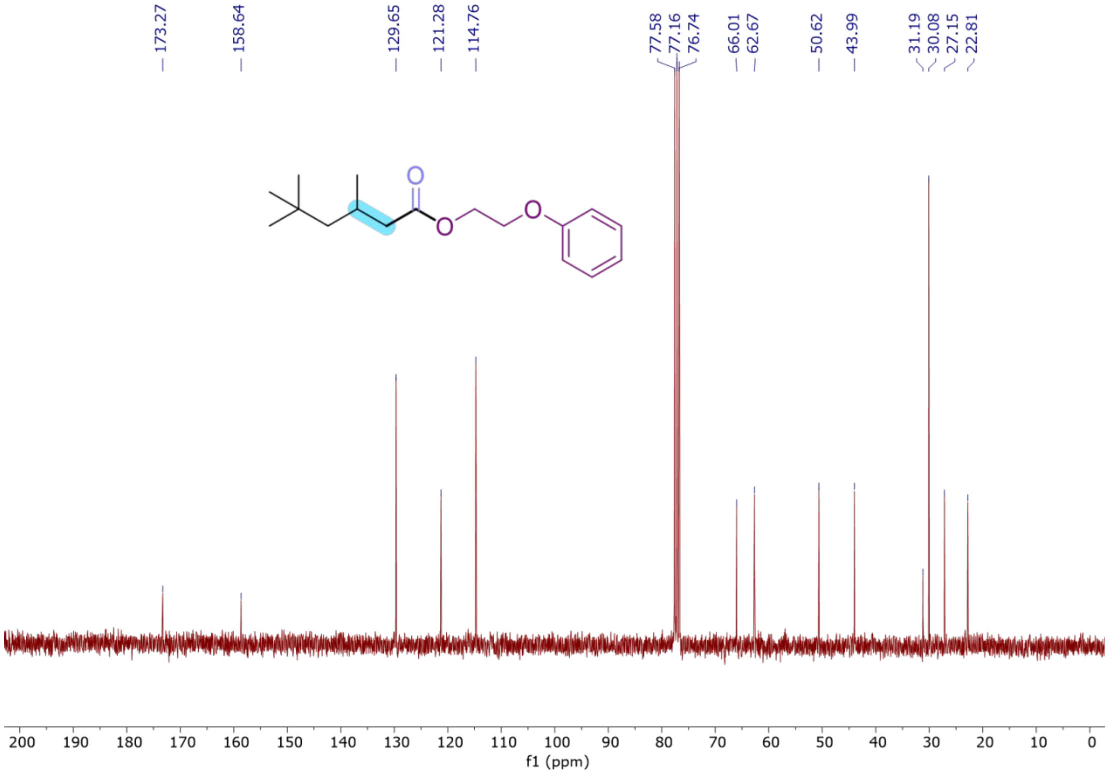

^1H NMR and ^{13}C NMR of **38**, (300 MHz, $CDCl_3$).

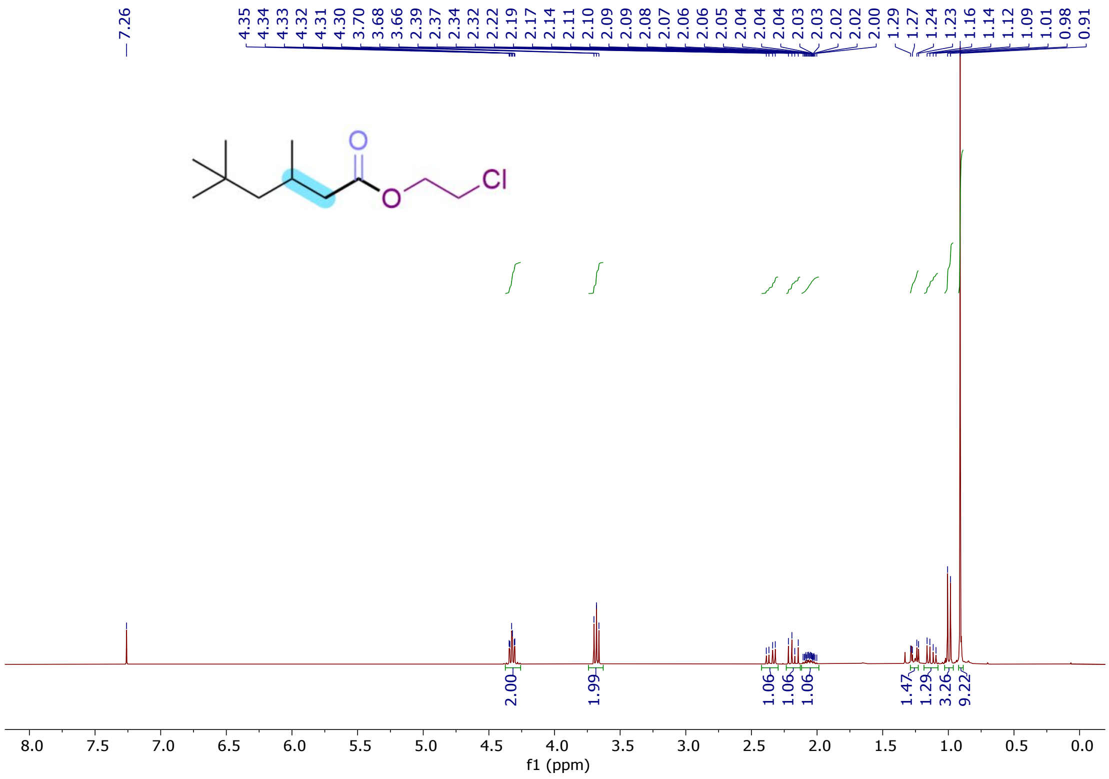

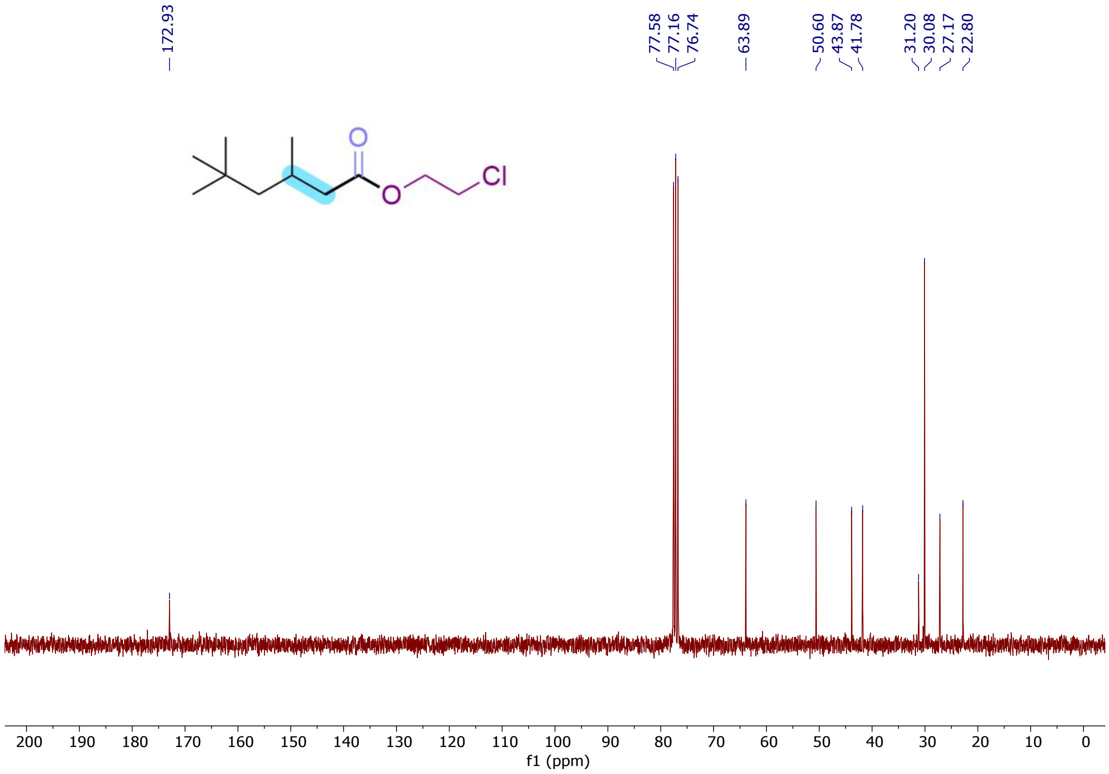

^{1}H NMR and ^{13}C NMR of **39**, (300 MHz, $CDCl_3$).

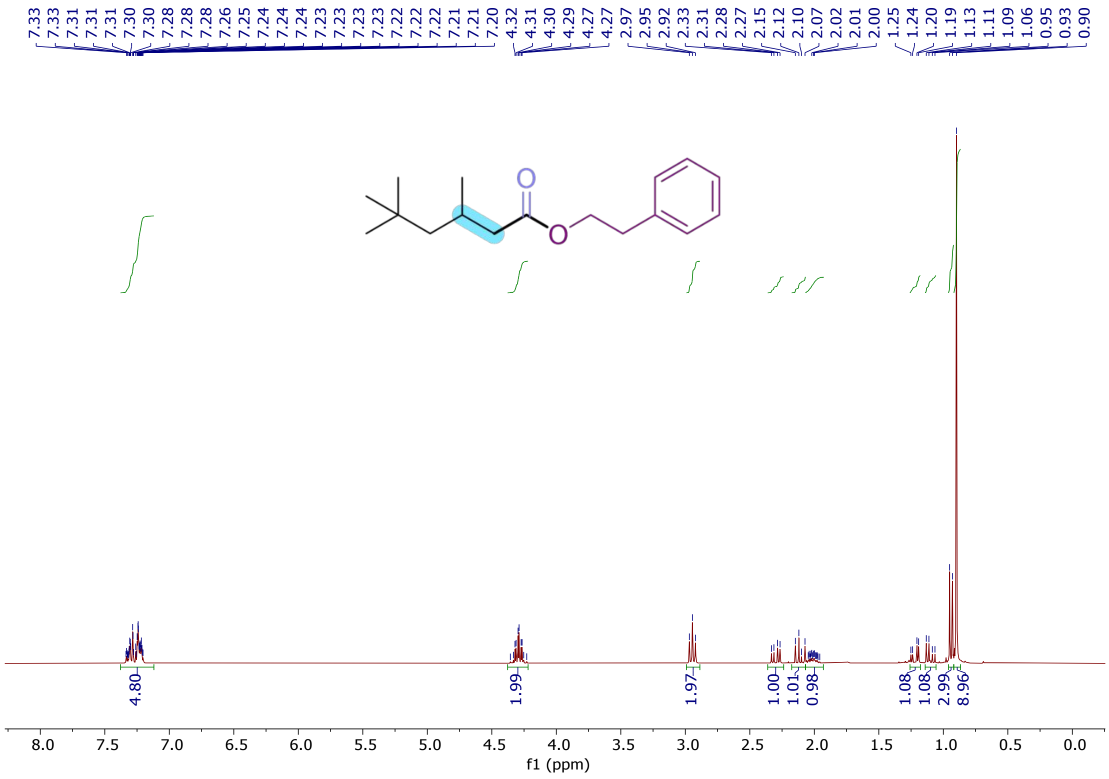

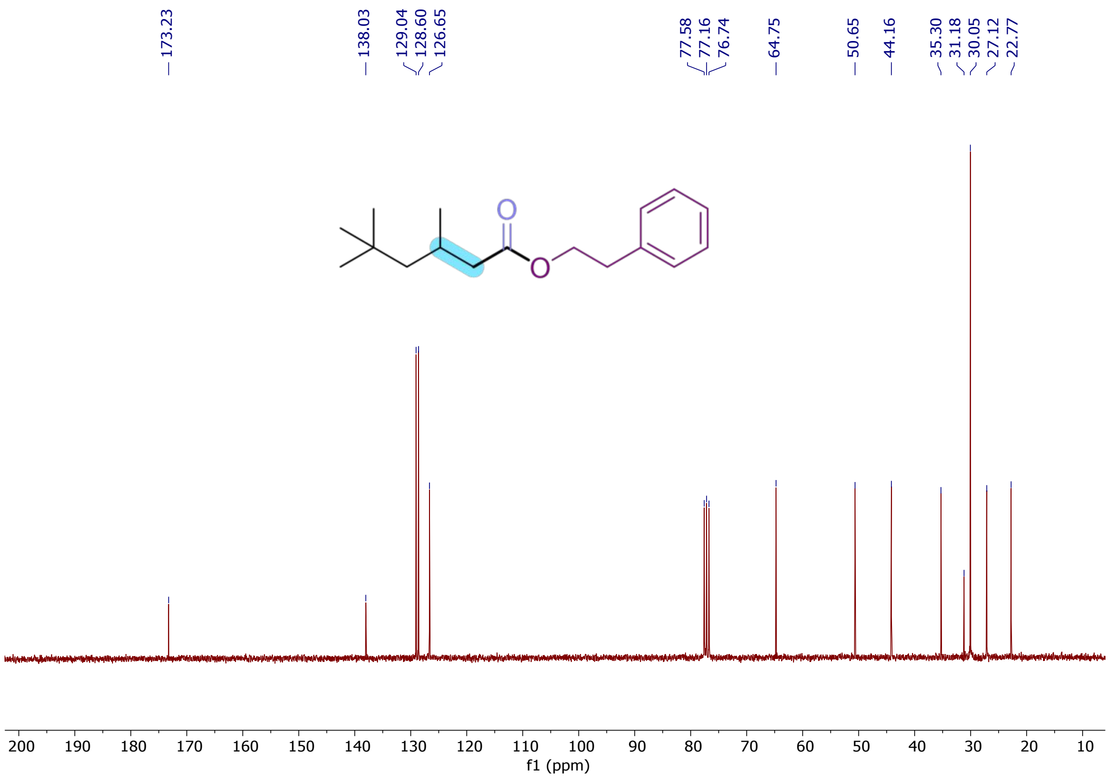

^{1}H NMR and ^{13}C NMR of **40**, (300 MHz, $CDCl_3$).

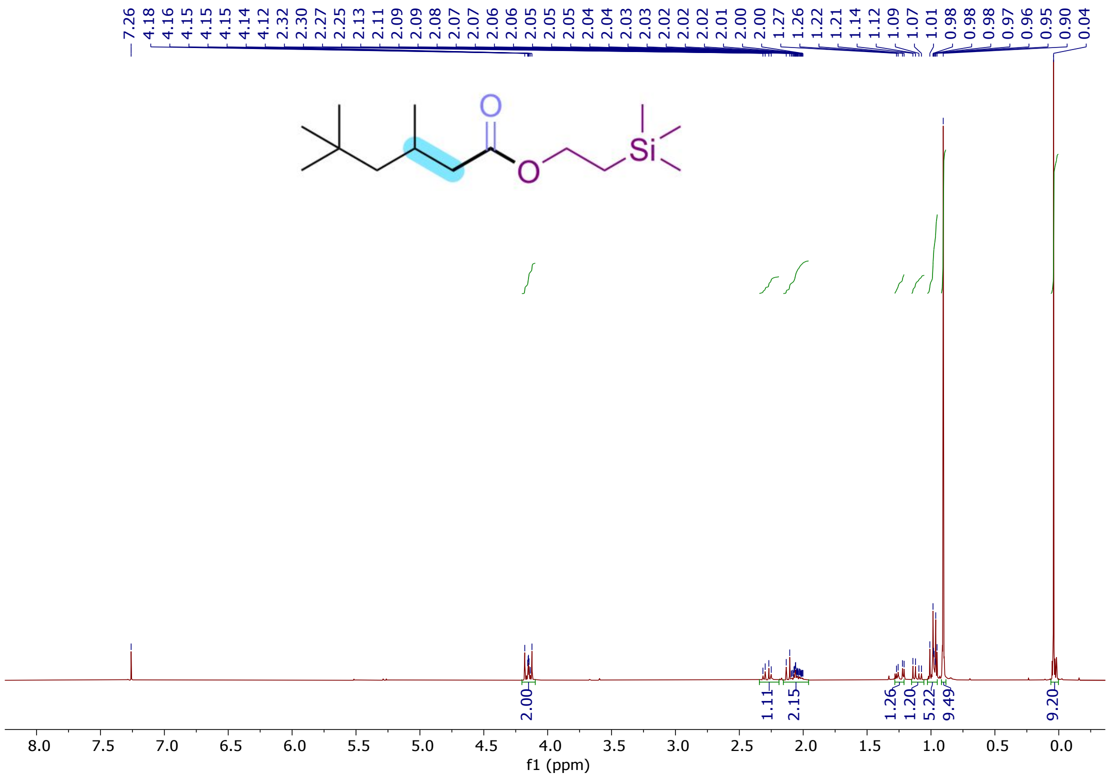

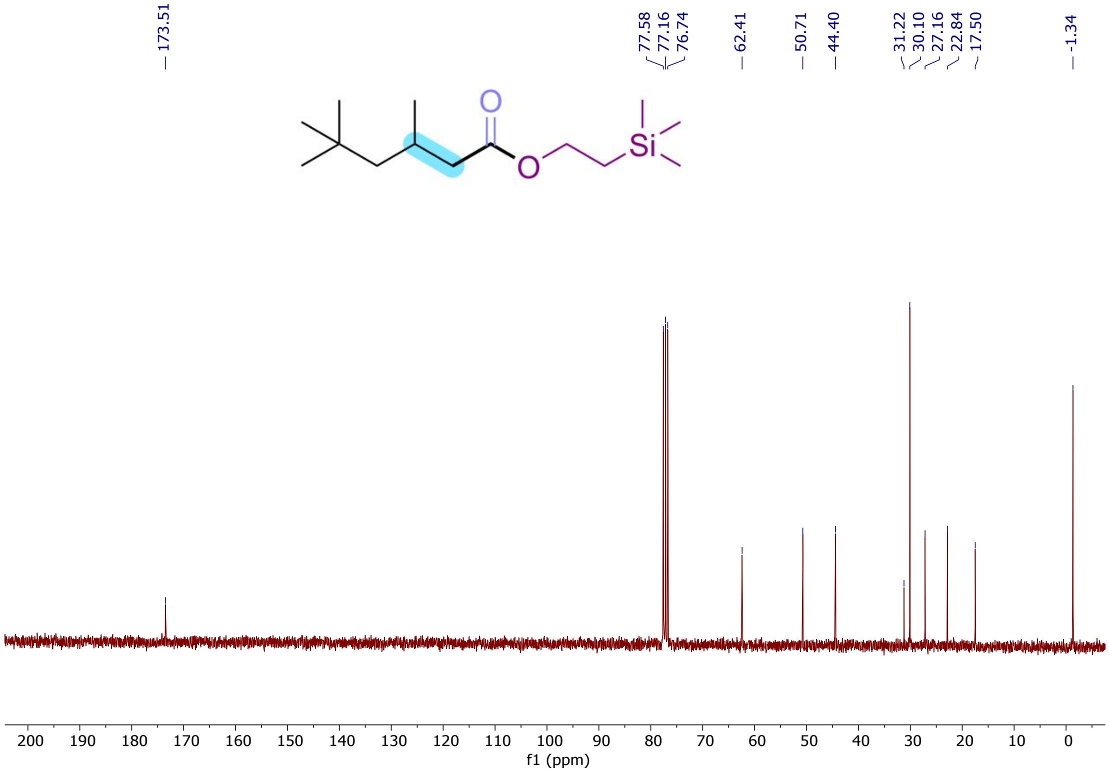

^{1}H NMR and ^{13}C NMR of **41**, (300 MHz, $CDCl_3$).

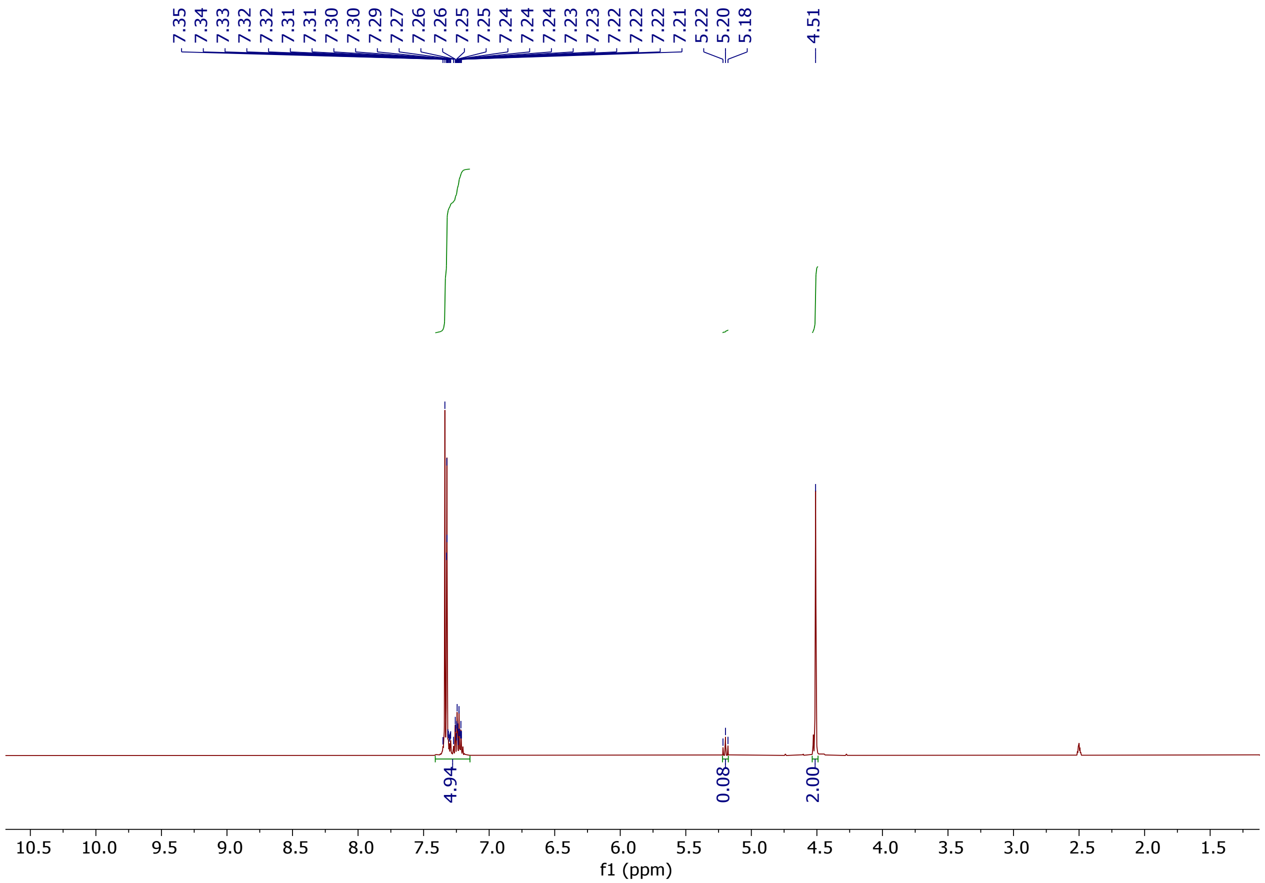

^{1}H NMR of BnOD (300 MHz, DMSO).

Reference

(1) Edgell, W. F.; Lyford IV, J. Preparation of sodium cobalt tetracarbonyl. *Inorg. Chem.* **1970**, *9* (8), 1932-1933.

(2) Utsunomiya, M.; Kondo, R.; Oshima, T.; Safumi, M.; Suzuki, T.; Obora, Y. Cross β-arylmethylation of alcohols catalysed by recyclable Ti–Pd alloys not requiring pre-activation. *Chem. Commun.* **2021**, *57* (42), 5139-5142.

(3) Tian, Z.; Pawlow, A.; Poutsma, J. C.; Kass, S. R. Are Carboxyl Groups the Most Acidic Sites in Amino Acids? Gas-Phase Acidity, H/D Exchange Experiments, and Computations on Cysteine and Its Conjugate Base. *J. Am. Chem. Soc.* **2007**, *129* (17), 5403-5407.

(4) Frisch, M.; Trucks, G.; Schlegel, H.; Scuseria, G.; Robb, M.; Cheeseman, J.; Scalmani, G.; Barone, V.; Petersson, G.; Nakatsuji, H. Gaussian Inc 16, revision A. 03. *Gaussian Inc.: Wallingford, CT* **2016**.

(5) Huo, C.-F.; Li, Y.-W.; Wu, G.-S.; Beller, M.; Jiao, H. Structures and Energies of [Co $(CO)_n]^m$ (m= 0, 1+, 1−) and HCo $(CO)_n$: Density Functional Studies. *J. of Phys. Chem. A* **2002**, *106* (50), 12161-12169.

(6) Becke, A. D. Density-functional thermochemistry. III. The role of exact exchange. *J. Chem. Phys.* **1993**, *98* (7), 5648-5652.

(7) Marenich, A. V.; Cramer, C. J.; Truhlar, D. G. Universal solvation model based on solute electron density and on a continuum model of the solvent defined by the bulk dielectric constant and atomic surface tensions. *J. Phys.Chem. B* **2009**, *113* (18), 6378-6396.
(8) Neese, F. The ORCA program system. *WIREs Comput. Mo.l Sci.* **2012**, *2* (1), 73-78.
(9) Lerda, S.; Altun, A.; Faculak, M. S.; Alexanian, E. J.; Bistoni, G. Shedding Light on Photochemical Activation and Catalytic Mechanism of Cobalt-Catalyzed Alkene Hydroaminocarbonylation. *ACS Catal.* **2025**, *15* (19), 16506-16512.
(10) Ungváry, F.; Markó, L. Kinetics and mechanism of the decomposition of cobalt tetracarbonyl hydride to dicobalt octacarbonyl and hydrogen. *J. Organomet. Chem.* **1969**, *20* (1), 205-209.
(11) Zhao, Y.; Truhlar, D. G. The M06 suite of density functionals for main group thermochemistry, thermochemical kinetics, noncovalent interactions, excited states, and transition elements: two new functionals and systematic testing of four M06-class functionals and 12 other functionals. *Theor. Chem. Acc.* **2008**, *120* (1), 215-241.
(12) Tuba, R.; Mika, L. T.; Bodor, A.; Pusztai, Z.; Tóth, I.; Horváth, I. T. Mechanism of the pyridine-modified cobalt-catalyzed hydromethoxycarbonylation of 1, 3-butadiene. *Organometallics* **2003**, *22* (8), 1582-1584.
